# Physiologically Based Pharmacokinetic Models of Probenecid and Furosemide to Predict Transporter Mediated Drug-Drug Interactions

**DOI:** 10.1007/s11095-020-02964-z

**Published:** 2020-11-25

**Authors:** Hannah Britz, Nina Hanke, Mitchell E. Taub, Ting Wang, Bhagwat Prasad, Éric Fernandez, Peter Stopfer, Valerie Nock, Thorsten Lehr

**Affiliations:** 1 Clinical Pharmacy, Saarland University, Campus C2 2, 66123 Saarbrücken, Germany; 2 Drug Metabolism and Pharmacokinetics, Boehringer Ingelheim Pharmaceuticals Inc., Ridgefield, Connecticut USA; 3 Department of Pharmaceutical Sciences, Washington State University, Spokane, Washington USA; 4 Translational Medicine and Clinical Pharmacology, Boehringer Ingelheim Pharma GmbH & Co. KG, Biberach, Germany

**Keywords:** drug-drug interaction (DDI), furosemide, organic anion transporter (OAT), physiologically based pharmacokinetic modeling (PBPK), probenecid

## Abstract

**Purpose:**

To provide whole-body physiologically based pharmacokinetic (PBPK) models of the potent clinical organic anion transporter (OAT) inhibitor probenecid and the clinical OAT victim drug furosemide for their application in transporter-based drug-drug interaction (DDI) modeling.

**Methods:**

PBPK models of probenecid and furosemide were developed in PK-Sim®. Drug-dependent parameters and plasma concentration-time profiles following intravenous and oral probenecid and furosemide administration were gathered from literature and used for model development. For model evaluation, plasma concentration-time profiles, areas under the plasma concentration–time curve (AUC) and peak plasma concentrations (C_max_) were predicted and compared to observed data. In addition, the models were applied to predict the outcome of clinical DDI studies.

**Results:**

The developed models accurately describe the reported plasma concentrations of 27 clinical probenecid studies and of 42 studies using furosemide. Furthermore, application of these models to predict the probenecid-furosemide and probenecid-rifampicin DDIs demonstrates their good performance, with 6/7 of the predicted DDI AUC ratios and 4/5 of the predicted DDI C_max_ ratios within 1.25-fold of the observed values, and all predicted DDI AUC and C_max_ ratios within 2.0-fold.

**Conclusions:**

Whole-body PBPK models of probenecid and furosemide were built and evaluated, providing useful tools to support the investigation of transporter mediated DDIs.

**Supplementary Information:**

The online version contains supplementary material available at 10.1007/s11095-020-02964-z.

## Introduction

Many important drug transporters are members of the solute carrier (SLC) family, which is widely expressed throughout the human body and mediates influx or efflux of endogenous and exogenous substrates (1). For the approval of new drugs, the U.S. Food and Drug Administration (FDA) and the European Medicines Agency (EMA) require various *in vitro*, and in many cases, clinical studies to characterize the transporter mediated drug-drug interaction (DDI) potential of investigational drugs. Based on the outcome of these investigations, recommendations for dose adjustments are given in the accompanying label of a new drug (2,3).

Organic anion transporter (OAT) 1 and OAT3 are members of the SLC transporter family (*SLC22A6, SLC22A8*) and recognized as important drug transporters from the perspective of their potential to be involved in clinically relevant DDIs. OAT1 and OAT3 are predominantly expressed in the kidney at the basolateral membrane of proximal tubule cells (4), where they facilitate the uptake of endogenous (e.g. p-aminohippurate, estrone sulfate) and exogenous (e.g. diuretic drugs) organic anions from the blood into the proximal tubule cells (5), from where they can be secreted into the nephron lumen for excretion with the urine. Several polymorphisms have been identified and investigated; however, variants of OAT1 or OAT3 have not been shown to significantly impact the renal clearance of OAT substrates in clinical studies (6,7). To characterize the OAT mediated DDI potential, the FDA recommends furosemide as clinical OAT1/OAT3 substrate and probenecid as clinical OAT1/OAT3 inhibitor (8). In addition, probenecid can also be used to investigate organic anion transporting polypeptide (OATP) 1B1 mediated DDIs (9).

OATP1B1, another clinically relevant member of the SLC transporter family (*SLCO1B1*), is exclusively expressed at the sinusoidal membrane of hepatocytes, where it is responsible for the uptake of endogenous (e.g. bile acids) and exogenous (e.g. statins, rifampicin) organic anions from the blood into the hepatocytes (10,11). As probenecid also inhibits OATP1B1, the prediction of the probenecid-rifampicin DDI was included into the presented study, to further evaluate the performance of the probenecid model as a DDI perpetrator.

Physiologically based pharmacokinetic (PBPK) modeling is encouraged and supported by the FDA and EMA as a valuable tool to quantitatively describe and predict the pharmacokinetics of drugs, to evaluate DDI potential and to support clinical study design, dose selection and labeling during drug development (2,3,12–14). The objectives of this study were to provide whole-body PBPK models of probenecid and furosemide, incorporating the transporters and enzymes involved in the pharmacokinetics of these drugs. *In vitro* measurements were used to parametrize the respective incorporated processes. The models were built and evaluated to adequately predict the plasma concentration-time profiles and the fractions excreted unchanged in urine. Furthermore, the models were used to predict probenecid DDIs, with probenecid as potent clinical OAT1/OAT3 inhibitor (8,15), moderate OATP1B1 inhibitor (9), weak inhibitor of multidrug resistance-associated protein (MRP) 4 (16) and weak inhibitor of uridine 5′-diphospho-glucuronosyltransferase (UGT) 1A9 (in-house measurement), furosemide as clinical OAT1/OAT3 substrate and rifampicin as OATP1B1 substrate. The comprehensive Electronic Supplementary Material (ESM) to this manuscript provides detailed information on the developed PBPK models, including all model parameters and a complete documentation of the extensive model evaluation. The model files will be shared in the Open Systems Pharmacology PBPK model library (www.open-systems-pharmacology.org).

## Methods

### Software

PBPK modeling was performed with the open source PK-Sim® and MoBi® modeling software (version 8.0, part of the Open Systems Pharmacology Suite (17,18), www.open-systems-pharmacology.org). Published plasma concentration-time profiles were digitized using GetData Graph Digitizer (version 2.26.0.20, S. Fedorov) (19). Parameter optimizations were accomplished with the Monte Carlo algorithm as well as the Levenberg-Marquardt algorithm using the “multiple random starting values” function implemented in PK-Sim®. The final optimizations were run using the Levenberg-Marquardt algorithm. PK parameter analysis and calculation of model performance measures was performed with R (version 3.6.2, The R Foundation for Statistical Computing) and graphics were compiled with R and RStudio (version 1.2.5033, RStudio, Inc., Boston, MA, USA). Sensitivity analysis was performed using the implemented Sensitivity Analysis tool in PK-Sim® (20).

### PBPK Model Building

PBPK model building was started with an extensive literature search to collect physicochemical parameters, information on absorption, distribution, metabolism and excretion (ADME) processes and clinical studies of intravenous and oral administration of probenecid and furosemide in single- and multiple dose regimens.

To build the datasets for PBPK model development, the reported observed plasma concentration-time profiles were digitized and divided into a training dataset for model building and a test dataset for model evaluation. Model input parameters that could not be informed from experimental reports were optimized by fitting the model simultaneously to the observed data of all studies assigned to the training dataset. To limit the parameters to be optimized during model building, the minimal number of processes necessary to mechanistically describe the pharmacokinetics and DDIs of the modeled drugs were implemented into the models. If two transporters show very similar expression patterns and affinity for the same compound, optimizing the transport rate constants of both transporters would lead to identifiability issues. Therefore, only the transporter with the higher affinity for the respective substrate was implemented, to describe a transport that probably is accomplished by both transporters *in vivo*.

Model evaluation was carried out based on the clinical data of the test dataset. Descriptive (training dataset) and predictive (test dataset) performance of the model for all analyzed clinical studies is transparently documented in the ESM.

### Virtual Individuals

The PBPK models were built based on data from healthy individuals, using the reported sex, ethnicity and mean values for age, weight and height from each study protocol. If no demographic information was provided, the following default values were substituted: male, European, 30 years of age, 73 kg body weight and 176 cm body height (characteristics from the PK-Sim® population database (21,22)). ADME transporters and enzymes were implemented in accordance with literature, using the PK-Sim® expression database to define their relative expression in the different organs of the body (23). Table S7.0.1 summarizes all system-dependent parameters on the implemented transporters and enzymes.

### Virtual Population Characteristics

To predict the variability of the simulated plasma concentration-time profiles, virtual populations of 100 individuals were generated, consisting of either European or Asian individuals. The percentage of female individuals and the ranges of age, weight and height were set according to the reported demographics. If not specified, virtual populations containing 100 male subjects 20–50 years of age were used, with body weight and height restrictions from the PK-Sim® population database (22). For details on the study populations see Tables S2.2.1, S3.2.1, S5.2.1 and S6.2.1.

In the generated virtual populations, organ volumes, tissue compositions, blood flow rates, etc. were varied by an implemented algorithm within the limits of the International Commission on Radiological Protection (ICRP) (21,22) or Tanaka (24) databases. In addition, the reference concentrations of the implemented transporters and enzymes were log-normally distributed around their mean values, using reported variabilities for their expression from the PK-Sim® database (25) or from literature. Table S7.0.1 summarizes the modeled transporters and enzymes with their reference concentrations and variabilities.

As the clinical plasma concentration data from literature is mostly reported as arithmetic means ± standard deviation, population prediction arithmetic means and 68% prediction intervals were plotted, that correspond to a range of ±1 standard deviation around the mean assuming normal distribution.

### PBPK Model Evaluation

Model performance was evaluated using various methods. The population predicted plasma concentration-time profiles were compared to the data observed in the clinical studies. Furthermore, predicted plasma concentrations of all studies were compared to the observed plasma concentrations in goodness-of-fit plots. In addition, the model performance was evaluated by comparison of predicted to observed areas under the plasma concentration-time curve (AUC) from the time of drug administration to the time of the last concentration measurement (AUC_last_) and peak plasma concentration (C_max_) values. As quantitative measures of the model performance, the mean relative deviation (MRD) of all predicted plasma concentrations (Eq. 1) and the geometric mean fold error (GMFE) of all predicted AUC_last_ and C_max_ values (Eq. 2) were calculated. MRD and GMFE values ≤2 characterize an adequate model performance.

1 $$ \mathrm{MRD}={10}^{\mathrm{x}};\kern0.5em \mathrm{x}=\sqrt{\frac{1}{\mathrm{k}}\ \sum \limits_{\mathrm{i}=1}^{\mathrm{k}}{\left({\log}_{10}{\mathrm{c}}_{\mathrm{predicted},\mathrm{i}}-{\log}_{10}{\mathrm{c}}_{\mathrm{observed},\mathrm{i}}\right)}^2} $$

where c_predicted, i_ = predicted plasma concentration, c_observed, i_ = corresponding observed plasma concentration and k = number of observed values.

2 $$ \mathrm{GMFE}={10}^{\mathrm{x}};\kern0.5em \mathrm{x}=\frac{1}{\mathrm{m}}\ \sum \limits_{\mathrm{i}=1}^{\mathrm{m}}\left|{\log}_{10}\left(\frac{\mathrm{predicted}\ \mathrm{PK}\ {\mathrm{parameter}}_{\mathrm{i}}}{\mathrm{observed}\ \mathrm{PK}\ {\mathrm{parameter}}_{\mathrm{i}}}\right)\right| $$

where predicted PK parmeter_i_ = predicted AUC_last_ or C_max_, observed PK parameter_i_ = corresponding observed AUC_last_ or C_max_ and m = number of studies.

Furthermore, sensitivity analysis results were assessed. A detailed description of the sensitivity calculation is provided in the ESM.

### PBPK DDI Modeling

**Fig. 1:**
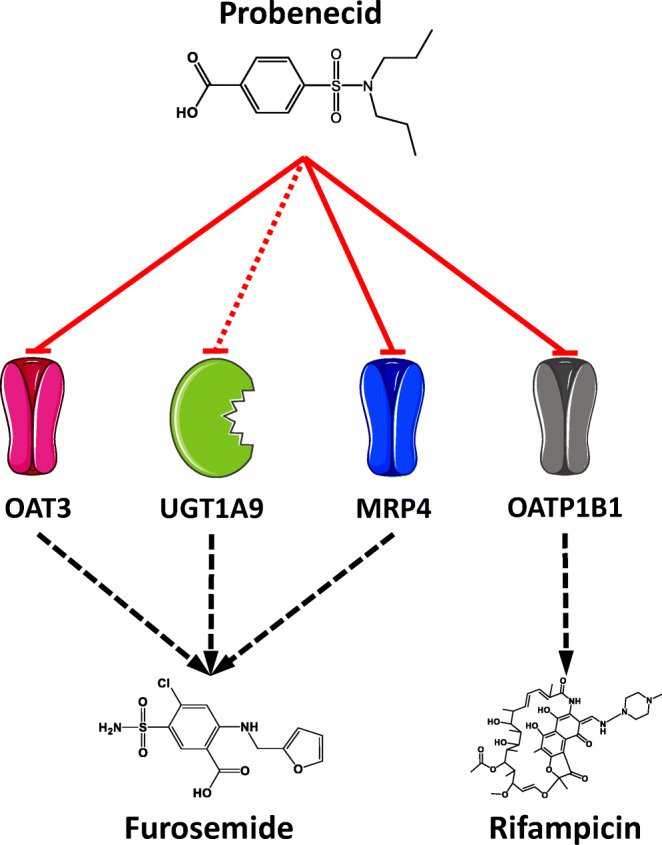
**Probenecid DDIs.** Schematic illustration of the modeled DDIs with probenecid as OAT3, UGT1A9, MRP4 and OATP1B1 perpetrator drug, furosemide as OAT3, UGT1A9 and MRP4 victim drug and rifampicin as OATP1B1 victim drug. The red solid lines indicate competitive inhibition, the red dotted line indicates non-competitive inhibition by probenecid. The black dashed lines indicate transport or metabolism. Drawings by Servier Medical Art, licensed under CC BY 3.0. **MRP4:** multidrug resistance-associated protein 4, **OAT3:** organic anion transporter 3, **OATP1B1:** organic anion transporting polypeptide 1B1, **UGT1A9:** uridine 5′-diphospho-glucuronosyltransferase 1A9.

As an additional means of model evaluation, the DDI performance of the developed models was assessed. To model the probenecid-furosemide DDI, inhibition of OAT3, UGT1A9 and MRP4 by probenecid was implemented. To predict the probenecid-rifampicin DDI, inhibition of OATP1B1 by probenecid was incorporated (Fig. 1). The rifampicin model applied was developed by Hanke *et al*. (26) and is freely available in the Open Systems Pharmacology repository on GitHub (27). The model includes rifampicin transport via OATP1B1 and P-glycoprotein (Pgp), metabolism via the arylacetamide deacetylase (AADAC), as well as auto-induction of OATP1B1, Pgp and AADAC (26). The good DDI performance of the model was demonstrated in many different applications (26,28–31). Mathematical implementation of the DDI processes is specified in Section 1 of the ESM.

Inhibition constants characterizing the inhibition of OAT3, UGT1A9 (in-house measurement) and OATP1B1 by probenecid were taken from *in vitro* experimental reports (9,15). Short descriptions of the respective *in vitro* assay conditions are provided in Sections 5.1 and 6.1 of the ESM. To describe the competitive inhibition of MRP4 by probenecid, the corresponding inhibition constant was optimized during the furosemide PBPK model parameter identification, using the clinical data of one of the probenecid-furosemide interaction studies (32) (see Table S5.2.1). The DDI parameter values are listed in the probenecid drug-dependent parameter table (Table S2.3.1).

### PBPK DDI Performance Evaluation

All DDI predictions were evaluated by comparison of predicted *versus* observed victim drug plasma concentration-time profiles alone and during co-administration, DDI AUC_last_ ratios and DDI C_max_ ratios (Eq. 3).

3 $$ \mathrm{DDI}\ \mathrm{PK}\ \mathrm{parameter}\ \mathrm{ratio}=\frac{\mathrm{PK}\ {\mathrm{parameter}}_{\mathrm{victim}\ \mathrm{drug}\ \mathrm{during}\ \mathrm{co}-\mathrm{administration}}}{\mathrm{PK}\ {\mathrm{parameter}}_{\mathrm{victim}\ \mathrm{drug}\ \mathrm{alone}}} $$

where PK parameter = AUC_last_ or C_max_.

As quantitative measure of the DDI prediction accuracy, GMFEs of the predicted DDI AUC_last_ ratios and DDI C_max_ ratios were calculated according to Eq. 2.

## Results

### PBPK Model Building and Evaluation

The probenecid PBPK model was developed using 27 different clinical studies, including intravenous (single dose) and oral (single- and multiple dose) administration. A complete list of the clinical studies used in the presented analysis is provided in Table S2.2.1. In addition, five studies reported fraction excreted unchanged in urine profiles following oral administration. In the intravenous studies, probenecid was administered in doses of 464–1860 mg. In the oral studies, probenecid was administered in doses of 250–2000 mg. The training dataset included 11 probenecid plasma concentration-time profiles and one fraction excreted unchanged in urine profile. The final probenecid model applies uptake into kidney cells via OAT3, glucuronidation mainly in the renal cells by UGT1A9, glomerular filtration and tubular reabsorption, which was modeled as a reduction of the glomerular filtration rate (GFR fraction <1). The drug-dependent parameters are summarized in Table S2.3.1.

**Fig. 2:**
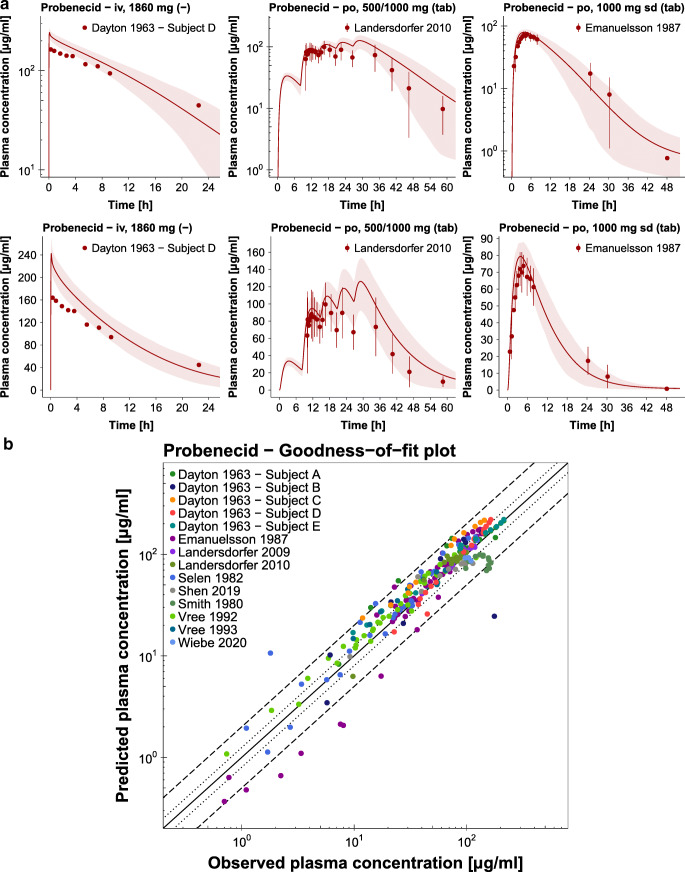
**Probenecid plasma concentrations.** (**a**) Selected population predictions of probenecid plasma concentration-time profiles compared to observed data in semilogarithmic (upper panel) and linear plots (lower panel). Observed data are shown as dots ± standard deviation (42,54,55). Population simulation arithmetic means are shown as lines; the shaded areas illustrate the predicted population variation (Q_16_ – Q_84_). (**b**) Predicted compared to observed probenecid plasma concentration values of all analyzed clinical studies. The solid line marks the line of identity. The dotted lines indicate 1.25-fold, the dashed lines indicate 2-fold deviation. Details on dosing regimens, study populations and literature references are summarized in Table S2.2.1. **iv:** intravenous, **po:** oral, **sd:** single dose, **tab:** tablet.

Population predicted compared to observed plasma concentration-time profiles of selected studies as well as the probenecid goodness-of-fit plot with the predicted plasma concentrations of all studies are presented in Fig. 2. Semilogarithmic and linear plots of the plasma profiles of all 27 clinical studies included in the analysis are shown in the ESM. Population predicted compared to observed fraction excreted unchanged in urine profiles are also presented in the ESM. Table S2.5.1 lists the MRD values of all 27 studies.

**Fig. 3:**
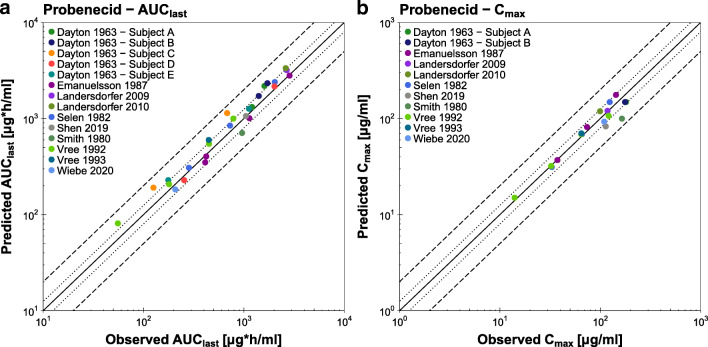
**Probenecid AUC**_**last**_ **and C**_**max**_ **values.** Predicted compared to observed probenecid (**a**) AUC_last_ and (**b**) C_max_ values of all analyzed clinical studies. The solid line marks the line of identity. The dotted lines indicate 1.25-fold, the dashed lines indicate 2-fold deviation. Details on dosing regimens, study populations and literature references are summarized in Table S2.2.1. The individual AUC_last_ and C_max_ values, mean GMFE values and ranges are listed in Table S2.5.2. **AUC**_**last**_**:** area under the plasma concentration-time curve from the time of drug administration to the time of the last concentration measurement, **C**_**max**_**:** peak plasma concentration.

The correlation of predicted and observed probenecid AUC_last_ and C_max_ values is presented in Fig. 3, further demonstrating the good model performance with 27/27 predicted AUC_last_ and 18/18 predicted C_max_ values within 2-fold of the observed data. The individual values, mean GMFE values and ranges are listed in Table S2.5.2.

The sensitivity analysis results of a simulation of 500 mg probenecid twice daily as a tablet are illustrated in Fig. S2.5.3. Applying a threshold of 0.5, the probenecid model is sensitive to the values of the UGT1A9 catalytic rate constant (optimized) and Michaelis-Menten constant (literature value), the probenecid fraction unbound in plasma (literature value), the OAT3 catalytic rate constant (optimized) and the probenecid lipophilicity (optimized).

The furosemide PBPK model was developed using 42 different clinical studies, including intravenous (single dose) and oral (single- and multiple dose) administration. A complete list of the clinical studies used in the presented analysis is provided in Table S3.2.1. In addition, 27 studies reported fraction excreted unchanged in urine profiles following intravenous and oral administration. In the intravenous studies, furosemide was administered in doses of 20–80 mg. In the oral studies, furosemide was administered in doses of 1–80 mg. The training dataset included 14 furosemide plasma concentration-time profiles, 11 fraction excreted unchanged in urine profiles and one plasma concentration-time profile with corresponding fraction excreted unchanged in urine data of furosemide during co-administration of probenecid (32). The final furosemide model applies uptake into kidney cells via OAT3, glucuronidation by UGT1A9, secretion into urine via MRP4 and glomerular filtration. The drug-dependent parameters are summarized in Table S3.3.1.

**Fig. 4:**
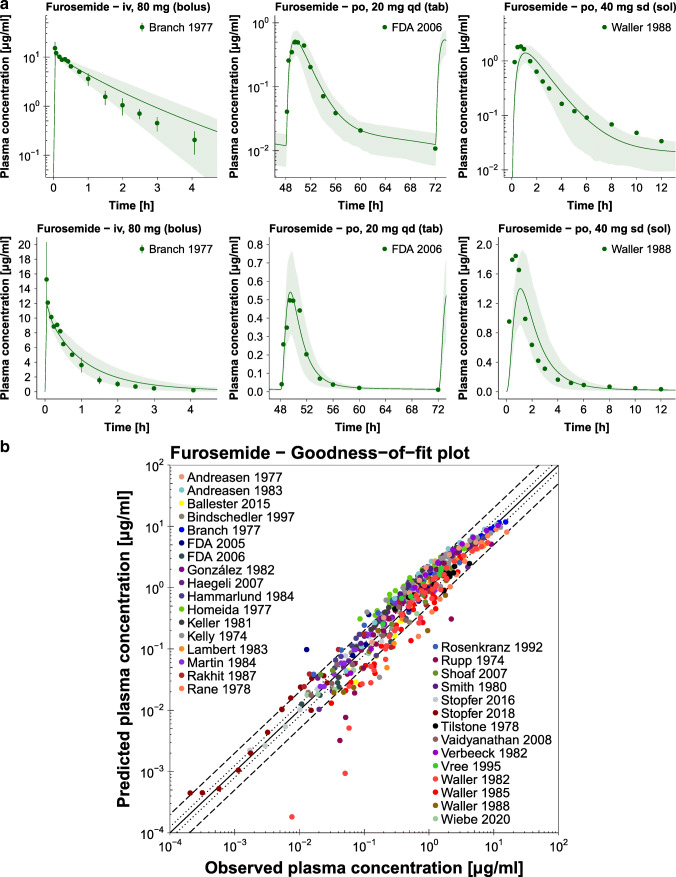
**Furosemide plasma concentrations.** (**a**) Selected population predictions of furosemide plasma concentration-time profiles compared to observed data in semilogarithmic (upper panel) and linear plots (lower panel). Observed data are shown as dots ± standard deviation (56–58). Population simulation arithmetic means are shown as lines; the shaded areas illustrate the predicted population variation (Q_16_ – Q_84_). (**b**) Predicted compared to observed furosemide plasma concentration values of all analyzed clinical studies. The solid line marks the line of identity. The dotted lines indicate 1.25-fold, the dashed lines indicate 2-fold deviation. Details on dosing regimens, study populations and literature references are summarized in Table S3.2.1. **iv:** intravenous, **po:** oral, **qd:** once daily, **sd:** single dose, **sol:** solution, **tab:** tablet.

Population predicted compared to observed plasma concentration-time profiles of selected studies as well as the furosemide goodness-of-fit plot with the predicted plasma concentrations of all studies are presented in Fig. 4. Semilogarithmic and linear plots of the plasma profiles of all 42 clinical studies included in the analysis are shown in the ESM. Population predicted compared to observed fraction excreted unchanged in urine profiles are also presented in the ESM. Table S3.5.1 lists the MRD values of all 42 studies.

**Fig. 5:**
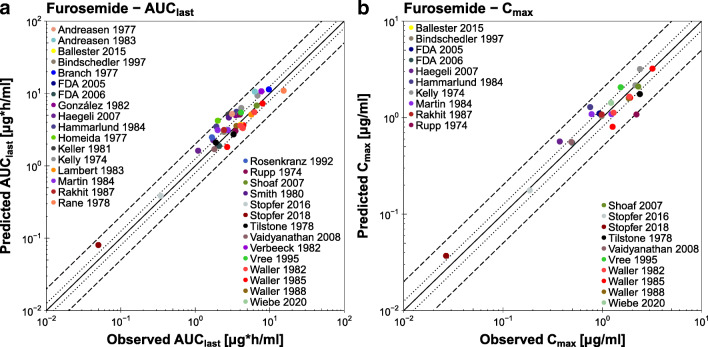
**Furosemide AUC**_**last**_ **and C**_**max**_ **values.** Predicted compared to observed furosemide (**a**) AUC_last_ and (**b**) C_max_ values of all analyzed clinical studies. The solid line marks the line of identity. The dotted lines indicate 1.25-fold, the dashed lines indicate 2-fold deviation. Details on dosing regimens, study populations and literature references are summarized in Table S3.2.1. The individual AUC_last_ and C_max_ values, mean GMFE values and ranges are listed in Table S3.5.2. **AUC**_**last**_**:** area under the plasma concentration-time curve from the time of drug administration to the time of the last concentration measurement, **C**_**max**_**:** peak plasma concentration.

The correlation of predicted and observed furosemide AUC_last_ and C_max_ values is presented in Fig. 5, further demonstrating the good model performance with 41/42 predicted AUC_last_ and 24/25 predicted C_max_ values within 2-fold of the observed data. The individual values, mean GMFE values and ranges are listed in Table S3.5.2.

The sensitivity analysis results of a simulation of 80 mg furosemide once daily as a tablet are illustrated in Fig. S3.5.3. Applying a threshold of 0.5, the furosemide model is sensitive to the values of furosemide fraction unbound in plasma (literature value) and the OAT3 catalytic rate constant (optimized).

### PBPK DDI Modeling and Evaluation

The developed PBPK models were applied to model the probenecid-furosemide and probenecid-rifampicin DDIs and the DDI performance was evaluated using the clinical data of six studies investigating the probenecid-furosemide DDI and one study of the probenecid-rifampicin DDI. For all studies, plasma concentration-time profiles of the victim drugs, administered alone and during probenecid co-administration, were predicted and compared to observed data. In addition, four studies of the probenecid-furosemide DDI reported fraction excreted unchanged in urine profiles, allowing the comparison of predicted and observed urinary excretion under control and DDI conditions. Administration protocols, study population details and references of the clinical DDI studies are listed in Tables S5.2.1 and S6.2.1.

To predict the probenecid-furosemide DDI, competitive inhibition of OAT3 (K_i_ = 5.41 μmol/l) (15) and non-competitive inhibition of UGT1A9 (K_i_ = 242.0 μmol/l) (in-house measurement) by probenecid were implemented using interaction parameter values measured *in vitro*. As no information regarding the inhibition of MRP4 could be obtained, the K_i_ to describe the competitive inhibition of MRP4 (K_i_ = 87.4 μmol/l) by probenecid was optimized during the parameter identification of the furosemide model. To predict the probenecid-rifampicin DDI, competitive inhibition of OATP1B1 (K_i_ = 39.8 μmol/l) (9) was implemented using an interaction parameter value measured *in vitro*.

**Fig. 6:**
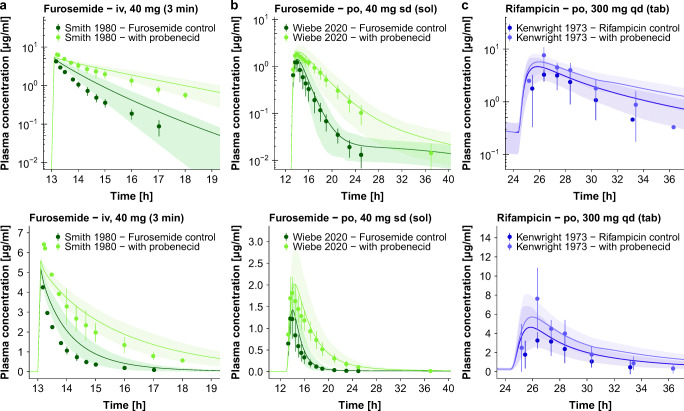
**DDI plasma concentration-time profiles.** Selected population predictions of the victim drug plasma concentration-time profiles compared to observed data for the (**a, b**) probenecid-furosemide and **(c)** probenecid-rifampicin DDIs in semilogarithmic (upper panel) and linear plots (lower panel). Observed data are shown as dots ± standard deviation (32,59,60). Population simulation arithmetic means are shown as lines; the shaded areas illustrate the corresponding predicted population variation (Q_16_ – Q_84_). Details on dosing regimens, study populations and literature references are summarized in Tables S5.2.1 and S6.2.1. **iv:** intravenous, **po:** oral, **qd:** once daily, **sd:** single dose, **tab:** tablet.

The coupled models adequately describe and predict all furosemide and rifampicin plasma concentration-time profiles, as well as fraction excreted unchanged in urine profiles of furosemide, under control conditions and during probenecid co-administration, over the full range of reported DDI administration protocols. Population predicted compared to observed plasma profiles of furosemide and rifampicin, administered alone and during the DDIs, are presented in Fig. 6 (selected DDI studies). Semilogarithmic and linear plots of all 7 clinical DDI studies included in the analysis are shown in Sections 5.3 and 6.3 of the ESM. Predicted compared to observed furosemide fraction excreted unchanged in urine profiles, administered alone and during probenecid co-administration, are presented in the ESM.

**Fig. 7:**
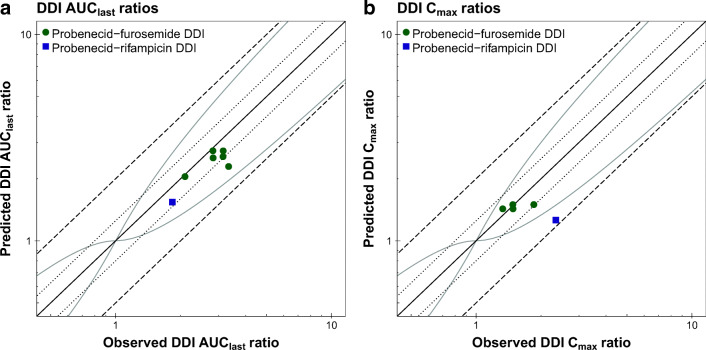
**DDI AUC**_**last**_ **and C**_**max**_ **ratios.** Predicted compared to observed (**a**) DDI AUC_last_ ratios and (**b**) DDI C_max_ ratios of the probenecid-furosemide and probenecid-rifampicin DDIs. The solid line marks the line of identity. The dotted lines indicate 1.25-fold, the dashed lines indicate 2-fold deviation. The curved grey lines show the prediction acceptance limits proposed by Guest *et al*. (61). Details on dosing regimens, the individual DDI AUC_last_ and DDI C_max_ ratios, mean GMFE values and ranges are listed in Table I. **AUC**_**last**_**:** area under the plasma concentration-time curve from the time of drug administration to the time of the last concentration measurement, **C**_**max**_**:** peak plasma concentration, **DDI:** drug-drug interaction.

The correlation of predicted and observed DDI AUC_last_ ratios and DDI C_max_ ratios of all analyzed clinical DDI studies is shown in Fig. 7, further demonstrating the good DDI performance with all predicted DDI ratios within 2-fold of the observed data. The individual ratios, mean GMFE values and ranges for both DDI combinations are listed in Table I.

**Table 1:** Predicted and Observed DDI AUC_last_ Ratios and DDI C_max_ Ratios with Mean GMFE Values and Ranges

Probenecid administration		Victim drug administration			DDI AUC_last_ ratio			DDI C_max_ ratio				Reference
Dose [mg]	Route	Dose [mg]	Route	Dosegap [h]	Pred	Obs	Pred/obs	Pred	Obs	Pred/obs	T_last_ [h]	
Intravenous furosemide												
500	po (−), qid (D1-D3)	40	iv (bolus), sd (D4)	2	2.52	2.83	0.89	–	–	–	3.0	Homeida 1977 (62)
1000	po (−), sd (D4)											
1000	po (tab), bid (D1)	40	iv (3 min), sd (D1)	1	2.29	3.34	0.69	–	–	–	4.0	Smith 1980 (59)
**GMFE**					**1.29 (1.12–1.45)**					–		
					**2/2 with GMFE ≤ 2**					–		
Oral furosemide												
1000	po (tab), bid (D1)	1	po (sol), sd (D1)	1	2.73	2.82	0.97	1.43	1.33	1.08	12.0	Wiebe 2020 (32)
1000	po (tab), bid (D1)	40	po (sol), sd (D1)	1	2.73	3.15	0.87	1.43	1.48	0.97	12.0	Wiebe 2020 (32)
1000	po (tab), sd (D1)	40	po (tab), sd (D1)	1	2.56	3.14	0.82	1.50	1.85	0.81	12.0	Shen 2019 (63)
1000	po (tab), sd (D1)	80	po (tab), sd (D1)	1	2.05	2.10	0.98	1.50	1.48	1.01	5.0	Vree 1995 (64)
**GMFE**					**1.11 (1.02–1.22)**			**1.09 (1.01–1.23)**				
					**4/4 with GMFE ≤ 2**			**4/4 with GMFE ≤ 2**				
**Overall GMFE of the** **probenecid-furosemide DDI**					**1.17 (1.02–1.45)**			**1.09 (1.01–1.23)**				
					**6/6 with GMFE ≤ 2**			**4/4 with GMFE ≤ 2**				
**DDI ratios within in the prediction** **success limits of Guest** ***et al***. (61)					**6/6 DDI AUC**_**last**_ **ratios**			**4/4 DDI C**_**max**_ **ratios**				
Oral rifampicin												
2000	po (−), sd (D2)	300	po (tab), qd (D1-D2)	0.33	1.54	1.83	0.84	1.26	2.34	0.54	11.0	Kenwright 1973 (60)
1500	po (−), sd (D2)^a^											
**Overall GMFE of the probenecid-rifampicin DDI**					**1.19**			**1.85**				
					**1/1 with GMFE ≤ 2**			**1/1 with GMFE ≤ 2**				
**DDI ratios within in the predictionsuccess limits of Guest** ***et al***. (61)					**1/1 DDI AUC**_**last**_ **ratio**			**0/1 DDI C**_**max**_ **ratio**				

^a^2000 mg probenecid 0.33 h before and 1500 mg 6 h after rifampicin administration, **AUC**_**last**_**:** area under the plasma concentration-time curve from the time of drug administration to the time of the last concentration measurement, **bid:** twice daily, **C**_**max**_**:** peak plasma concentration, **D:** day of administration, **DDI:** drug-drug interaction, **GMFE:** geometric mean fold error, **iv:** intravenous, **obs:** observed, **po:** oral, **pred:** predicted, **qid:** four times daily, **qd:** once daily**, route:** route of administration, **sd:** single dose, **sol:** solution, **tab:** tablet, **T**_**last**_**:** time of the last concentration measurement. GMFE values are means and ranges

Bold text marks the main results

## Discussion

The newly developed whole-body PBPK models for probenecid and furosemide accurately describe the observed plasma concentration-time profiles and fraction excreted unchanged in urine data over the full range of reported doses and administration protocols. Furthermore, these models adequately describe the available clinical data from probenecid-furosemide and probenecid-rifampicin clinical DDI studies.

Various other PBPK models of probenecid and furosemide with different applications have been published previously (33–38). For probenecid, three PBPK models are available describing DDIs with drugs that are OAT substrates. However, none of these analyses considered the probenecid-furosemide DDI or extended the model to include UGT1A9, MRP4 or OATP1B1 inhibition (33–35). The previously developed furosemide PBPK models were not built or evaluated for use in DDI prediction (36–38).

Development of the probenecid model was particularly challenging. The number of published clinical studies is low and the quality of the available data varies considerably, as probenecid was approved in the late 1940s. Hence, careful consideration of the study protocols and the presented data was required, and studies in patients or elderly volunteers, studies with co-medication and studies using others than the marketed formulation were excluded for probenecid model development. In addition, information on the ADME processes that govern the pharmacokinetics of probenecid is very limited.

Probenecid shows a low solubility and permeability, indicating an important role of transporters in its absorption and distribution. However, neither *in vitro* nor *in vivo* studies describing transporters involved in probenecid absorption, organ uptake, secretion or reabsorption are available in literature. Therefore, probenecid absorption was described by optimization of the passive transcellular intestinal permeability (optimized value: 3.97 · 10^−4^ cm/min, lipophilicity based calculated value: 3.12 · 10^−6^ cm/min). The uptake of probenecid into the kidney, which is its site of action and metabolism (39–41), was assumed to be mediated by OAT3. The parameters (K_M_, k_cat_) to describe this transport were optimized during parameter identification (see Table S2.3.1). Furthermore, a low renal clearance (42) and fraction excreted unchanged in urine of only 0.3% to 5% (43,44) are reported, indicating tubular reabsorption. Due to our current lack of knowledge regarding transporters that may contribute to probenecid reabsorption, the GFR fraction was optimized to 0.03. This reduced GFR fraction substitutes for the implementation of active reabsorption processes of probenecid (20) and correctly captures the low probenecid fraction excreted unchanged in urine.

In the clinical studies conducted by Vree *et al*. (43,44), probenecid tablets were broken in half prior to oral administration. The corresponding plasma concentration-time profiles display an earlier time to peak plasma concentration (T_max_) of 1.6 h compared to the other clinical studies with a T_max_ of 3.3 h. Given the low solubility of probenecid it is possible that the broken tablets show a different dissolution behavior, resulting in faster release and absorption. Therefore, a different dissolution profile was used to describe the studies by Vree *et al*. (43,44). The parameters to model the two different dissolution profiles are listed in Table S2.3.1.

Similar to probenecid, furosemide also demonstrates low solubility and permeability and is classified as a BCS class IV drug (45). Therefore, transporters play an essential role in furosemide absorption, distribution and elimination. Furosemide bioavailability is highly variable (37%–83%) (46), and influenced by dosage form and fasted/fed state of the patient (46). For the absorption of furosemide, Flanagan* et al*. postulate a saturable active transport process and passive diffusion with paracellular contribution in Caco-2 cells (47). As no further information on transporters that may contribute to the absorption of furosemide is available, absorption was modeled as passive transcellular (5.06 · 10^−7^ cm/min) and paracellular (2.32 · 10^−6^ cm/min) intestinal permeability. These processes together allow a rapid absorption in the small intestine to describe the early furosemide T_max_ of 1.0–1.5 h (46), while limiting the furosemide absorption in the large intestine. Other crucial transport processes take place in the kidney, which is the main organ for furosemide metabolism and excretion (fraction excreted unchanged in urine: 20%–80% (46)).

Uptake of furosemide into renal cells *in vivo* is probably facilitated via OAT1 and OAT3. Both transporters are predominantly expressed in the kidney (48) and show a similar affinity to furosemide (OAT1 furosemide K_M_ = 38.9 μmol/l, OAT3 furosemide K_M_ = 21.5 μmol/l (49)). Without additional information to distinguish the furosemide transport via these two transporters, the furosemide OAT1 and OAT3 transport rate constants would be highly correlated in a model parameter optimization. To avoid indentifiability issues, renal uptake of furosemide was incorporated via OAT3 only, as a substitute for transport by both, OAT1 and OAT3. The probenecid inhibition potency towards these transporters is also similar (probenecid OAT1 K_i_ = 11.4 μmol/l, probenecid OAT3 K_i_ = 5.41 μmol/l (15) and the DDI was predicted via inhibition of OAT3. Furosemide metabolism was modeled using UGT1A9 (50). The pronounced urinary excretion is accomplished by glomerular filtration and active tubular secretion. Renal secretion was incorporated via MRP4 (51,52), based on *in vitro* furosemide transport measurements.

In the goodness-of-fit plot, there seems to be an underprediction of the lower furosemide plasma concentrations for two of the intravenous studies (Rupp 1974 and Waller 1982). These are the only two studies that published furosemide plasma concentrations later than 6 h after intravenous dosing, and they did not report their lower limits of quantification. The model is therefore not qualified for the prediction of plasma concentrations later than 6 h after intravenous administration of furosemide. However, this does not affect the prediction of higher plasma concentrations and AUC_last_ values, and the developed model shows a good performance, with 41/42 predicted AUC_last_ and 24/25 predicted C_max_ values within 2-fold of the observed data.

To model the probenecid-furosemide DDI, inhibition of OAT3 alone, using published *in vitro* inhibition parameters, was not sufficient to describe the clinically observed data. Therefore, inhibition of UGT1A9 and MRP4 were added to adequately capture the impact of probenecid on the furosemide pharmacokinetics. Inhibition parameter values for OAT3 (15) and UGT1A9 (in-house measurement) were available from *in vitro* studies. Van Aubel *et al*. reported MRP4 inhibition by probenecid (16), but so far, no inhibition parameter values have been published. Therefore, competitive MRP4 inhibition was assumed and the K_i_ value was optimized during parameter identification of the furosemide model. Applying the inhibition of OAT3, UGT1A9 and MRP4, all reported plasma concentration time-profiles and fraction excreted unchanged in urine profiles of furosemide during probenecid co-administration are well described by the presented models.

The probenecid-rifampicin DDI was predicted using a probenecid OATP1B1 inhibition value measured with 2′,7′-dichlorofluorescein as the substrate (9). For OATP1B1, it has been demonstrated that its inhibition can strongly depend on the employed substrate (53). However, since there are no *in vitro* reports of probenecid OATP1B1 inhibition using rifampicin, the DDI was predicted applying the probenecid OATP1B1 K_i_ = 39.8 μmol/l measured with 2′,7′-dichlorofluorescein (9). Rifampicin is typically not used as OATP1B1 victim drug, but rather as OATP1B1/OATP1B3 inhibitor. However, as the clinical data of this probenecid-rifampicin trial were published, we wanted to utilize them to test our probenecid model. In addition to OATP1B1/OATP1B3 inhibition, rifampicin is also inhibiting and inducing further enzymes and transporters (13). Therefore, an impact of rifampicin on the perpetrator drug probenecid may have influenced the results of this particular DDI administration protocol. Taking into account that the only available clinical study has been published in 1973 and that the reported rifampicin plasma concentrations show considerable standard deviations, this DDI is also well described (see Fig. 6 and Table I).

## Conclusions

The presented whole-body PBPK models of probenecid and furosemide have been carefully built and evaluated for their ability to predict the pharmacokinetics of these drugs, using a multitude of clinical studies. In addition, the models adequately describe the available clinical data of the probenecid-furosemide and probenecid-rifampicin DDIs and will be shared in the Open Systems Pharmacology PBPK model library (www.open-systems-pharmacology.org) as tools to support the investigation of the DDI potential of new compounds during drug development. The ESM to this paper has been compiled to serve as transparent and comprehensive documentation of the probenecid and furosemide model development and evaluation.

## Supplementary Information

ESM 1 (PDF 3866 kb)

## Abbreviations

AADAC: Arylacetamide deacetylase
ADME: Absorption, distribution, metabolism and excretion
AUC_last_: Area under the plasma concentration-time curve (AUC) from the time of drug administration to the time of the last concentration measurement
C_max_: Peak plasma concentration
DDI: Drug-drug interaction
EMA: European Medicines Agency
FDA: U.S. Food and Drug Administration
GFR: Glomerular filtration rate
GMFE: Geometric mean fold error
MRD: Mean relative deviation
MRP: Multidrug resistance-associated protein
OAT: Organic anion transporter
OATP: Organic anion transporting polypeptide
PBPK: Physiologically based pharmacokinetics
Pgp: P-glycoprotein
SLC: Solute carrier
T_max_: Time to peak plasma concentration
UGT: Uridine 5′-diphospho-glucuronosyltransferase
