# Supplementary material for: Physiologically Based Pharmacokinetic Models of Probenecid and Furosemide to Predict Transporter Mediated Drug-Drug Interactions
Source: Pharm Res. 2020 Nov 25;37(12):250. doi: 10.1007/s11095-020-02964-z (PMC7688195; doi:10.1007/s11095-020-02964-z)
Supplement: Supplementary file 1 — (PDF 3866 kb) [file 11095_2020_2964_MOESM1_ESM.pdf]

Pharmaceutical Research

Physiologically based pharmacokinetic models of probenecid and furosemide to predict transporter mediated drug-drug interactions

Electronic Supplementary Material

Hannah Britz[1], Nina Hanke[1], Mitchell E. Taub[2], Ting Wang[2], Bhagwat Prasad[3], Éric Fernandez[4], Peter Stopfer[4], Valerie Nock[4] and Thorsten Lehr[1]

[1] Clinical Pharmacy, Saarland University, Saarbrücken, Germany
[2] Drug Metabolism and Pharmacokinetics, Boehringer Ingelheim Pharmaceuticals Inc., Ridgefield, Connecticut, USA
[3] Department of Pharmaceutical Sciences, Washington State University, Spokane, Washington, USA
[4] Translational Medicine and Clinical Pharmacology, Boehringer Ingelheim Pharma GmbH & Co. KG, Biberach, Germany

Funding:
This project has received funding from Boehringer Ingelheim Pharma GmbH & Co. KG.

Conflict of Interest:
Mitchell E. Taub, Ting Wang, Éric Fernandez, Peter Stopfer and Valerie Nock are employees of Boehringer Ingelheim. Thorsten Lehr has received research grants from Boehringer Ingelheim Pharma GmbH & Co. KG. Hannah Britz, Nina Hanke and Bhagwat Prasad declare that they have no conflict of interest.

Acknowledgements:
We would like to thank Solvo Biotechnology (Budapest, Hungary) for providing MRP4 vesicles used in the transporter studies. In vitro work on MRP4 mediated furosemide transport kinetics was conducted by Revathi Chapa and Bhagwat Prasad at the Department of Pharmaceutics, University of Washington, Seattle, WA, USA.

Corresponding Author:
Prof. Dr. Thorsten Lehr
Clinical Pharmacy, Saarland University
Campus C2 2, 66123 Saarbrücken, Germany
ORCID: 0000 0002 8372 1465
Phone: +49 681 302 70255
thorsten.lehr@mx.uni-saarland.de

Contents

1 Physiologically based pharmacokinetic (PBPK) modeling 4
1.1 PBPK model building . . . 4
1.1.1 PBPK model building . . . 4
1.1.2 Virtual individuals . . . 4
1.1.3 Virtual population characteristics . . . 5
1.2 PBPK model evaluation . . . 5
1.2.1 PBPK model evaluation . . . 5
1.2.2 PBPK model sensitivity analysis . . . 6
1.3 Drug-drug interaction (DDI) modeling . . . 6
1.3.1 PBPK DDI modeling . . . 6
1.3.2 PBPK DDI performance evaluation . . . 8
1.3.3 Mathematical implementation of DDIs . . . 8

2 PBPK modeling of probenecid 10
2.1 PBPK model development . . . 10
2.2 Clinical studies . . . 12
2.3 Drug-dependent parameters . . . 13
2.4 Profiles . . . 14
2.4.1 Semilogarithmic plots - Plasma - Population predictions . . . 14
2.4.2 Linear plots - Plasma - Population predictions . . . 17
2.4.3 Semilogarithmic plots - Plasma - Individual predictions . . . 20
2.4.4 Linear plots - Plasma - Individual predictions . . . 23
2.4.5 Linear plots - Fraction excreted unchanged in urine - Population predictions . 26
2.4.6 Linear plots - Fraction excreted unchanged in urine - Individual predictions . 27
2.5 Probenecid PBPK model evaluation . . . 28
2.5.1 Plasma concentration goodness-of-fit plot . . . 28
2.5.2 Mean relative deviation (MRD) of predicted plasma concentrations . . . 29
2.5.3 AUC_{last} and C_{max} goodness-of-fit plots . . . 30
2.5.4 Predicted and observed AUC_{last} and C_{max} values with mean GMFE values and ranges . . . 31
2.5.5 Sensitivity analysis . . . 33

3 PBPK modeling of furosemide 34
3.1 PBPK model development . . . 34
3.2 Clinical studies . . . 35
3.3 Drug-dependent parameters . . . 37
3.4 Profiles . . . 38
3.4.1 Semilogarithmic plots - Plasma - Population predictions . . . 38
3.4.2 Linear plots - Plasma - Population predictions . . . 43
3.4.3 Semilogarithmic plots - Plasma - Individual predictions . . . 48
3.4.4 Linear plots - Plasma - Individual predictions . . . 53
3.4.5 Linear plots - Fraction excreted unchanged in urine - Population predictions . 58
3.4.6 Linear plots - Fraction excreted unchanged in urine - Individual predictions . 61

3.5 Furosemide PBPK model evaluation 64
3.5.1 Plasma concentration goodness-of-fit plot 64
3.5.2 Mean relative deviation (MRD) of predicted plasma concentrations 65
3.5.3 AUC_{last} and C_{max} goodness-of-fit plots 66
3.5.4 Predicted and observed AUC_{last} and C_{max} values with mean GMFE values and ranges 67
3.5.5 Sensitivity analysis 69

4 PBPK modeling of rifampicin 70
4.1 Rifampicin PBPK model 70
4.2 Drug-dependent parameters 71

5 Probenecid-furosemide DDI 72
5.1 PBPK DDI modeling 72
5.2 Clinical studies 73
5.3 Profiles 74
5.3.1 Semilogarithmic plots - Plasma - Population predictions 74
5.3.2 Linear plots - Plasma - Population predictions 75
5.3.3 Semilogarithmic plots - Plasma - Individual predictions 76
5.3.4 Linear plots - Plasma - Individual predictions 77
5.3.5 Linear plots - Fraction excreted unchanged in urine - Population predictions . 78
5.3.6 Linear plots - Fraction excreted unchanged in urine - Individual predictions . 79
5.4 PBPK DDI performance evaluation 80
5.4.1 DDI AUC_{last} and DDI C_{max} ratio goodness-of-fit plots 80
5.4.2 Predicted and observed DDI AUC_{last} and DDI C_{max} ratios with mean GMFE values and ranges 81

6 Probenecid-rifampicin DDI 82
6.1 PBPK DDI modeling 82
6.2 Clinical studies 83
6.3 Profiles 84
6.3.1 Semilogarithmic and linear plots - Plasma - Population predictions 84
6.3.2 Semilogarithmic and linear plots - Plasma - Individual predictions 85
6.4 PBPK DDI performance evaluation 86
6.4.1 DDI AUC_{last} and DDI C_{max} ratio goodness-of-fit plots 86
6.4.2 Predicted and observed DDI AUC_{last} and DDI C_{max} ratios with GMFE values 87

7 System-dependent parameters 88

8 Abbreviations 89

References 91

1 Physiologically based pharmacokinetic (PBPK) modeling

1.1 PBPK model building

1.1.1 PBPK model building

Physiologically based pharmacokinetic (PBPK) modeling was performed with the open source PK-Sim® and MoBi® modeling software (version 8.0, part of the Open Systems Pharmacology Suite [1, 2], www.open-systems-pharmacology.org). Published plasma concentration-time profiles were digitized using GetData Graph Digitizer (version 2.26.0.20, S.Fedorov) [3]. Parameter optimizations were accomplished with the Monte Carlo algorithm as well as the Levenberg-Marquardt algorithm using the "multiple random starting values" function implemented in PK-Sim®. The final optimizations were run using the Levenberg-Marquardt algorithm. Pharmacokinetic (PK) parameter analysis and calculation of model performance measures was performed with R (version 3.6.2, The R Foundation for Statistical Computing) and graphics were compiled with R and RStudio (version 1.2.5033, RStudio, Inc., Boston, MA, USA). Sensitivity analysis was performed using the implemented Sensitivity Analysis tool in PK-Sim® [4].
PBPK model building began with an extensive literature search to collect physicochemical parameters, information on absorption, distribution, metabolism and excretion (ADME) processes and clinical studies of intravenous (iv) and oral (po) administration of the drugs in single- and multiple dose regimens. In addition to drug plasma concentration-time profiles, further clinical data on fraction excreted in urine or feces were integrated whenever available. To build the datasets for PBPK modeling, the reported observed plasma concentration-time profiles were digitized and divided into a training dataset for model building and a test dataset for model evaluation (see Tables S2.2.1, S3.2.1, S5.2.1 and S6.2.1). Model input parameters that could not be informed from experimental reports were optimized by fitting the model simultaneously to the observed data of all studies assigned to the training dataset. To limit the parameters to be optimized during model building, the minimal number of processes necessary to mechanistically describe the pharmacokinetics and drug-drug interactions (DDIs) of the modeled drugs were implemented into the models. If two transporters show very similar expression patterns and affinity for the same compound, optimizing the transport rate constants of both transporters would lead to identifiability issues. Therefore, only the transporter with the higher affinity for the respective substrate was implemented, to describe a transport that probably is accomplished by both transporters in vivo.

1.1.2 Virtual individuals

The PBPK models were built based on data from healthy individuals, using the reported sex, ethnicity, and mean values for age, weight and height from each study protocol. If no demographic information was provided, the following default values were substituted: male, European, 30 years of age, 73 kg body weight and 176 cm body height (characteristics from the PK-Sim® population database [5, 6]). ADME transporters and enzymes were implemented in accordance with literature, using the PK-Sim® expression database to define their relative expression in the different organs of the body [7]. Table S7.0.1 summarizes all system-dependent parameters on the implemented

transporters and enzymes.

1.1.3 Virtual population characteristics

To predict the variability of the simulated plasma concentration-time profiles, virtual populations of 100 individuals were generated, consisting of either European or Asian individuals. The percentage of female individuals and the ranges of age, weight and height were set according to the reported demographics. If not specified, virtual populations containing 100 male subjects with 20–50 years of age were used, with body weight and height restrictions from the PK-Sim® population database [6]. Details on the respective study populations are provided in the Clinical Study Tables.
In the generated virtual populations, organ volumes, tissue compositions, blood flow rates etc. were varied by an implemented algorithm within the limits of the International Commission on Radiological Protection (ICRP) [5, 6] or Tanaka [8] databases. In addition, the reference concentrations of the implemented transporters and enzymes were log-normally distributed around their mean values, using reported variabilities for their expression from the PK-Sim® database [4] or from literature. The System-dependent parameter Table (Table S7.0.1) summarizes the modeled transporters and enzymes with their reference concentrations and variabilities. As the clinical plasma concentration data from literature is mostly reported as arithmetic means $\pm$ standard deviations, population prediction arithmetic means and 68% prediction intervals were plotted, that correspond to a range of $\pm$ 1 standard deviation around the mean assuming normal distribution.

1.2 PBPK model evaluation

1.2.1 PBPK model evaluation

Model evaluation was carried out with different methods based on the clinical data of the test dataset. Descriptive (training dataset) and predictive (test dataset) performance of the model for all analyzed clinical studies is transparently documented in this document. The population predicted plasma concentration-time profiles were compared to the data observed in the clinical studies. Furthermore, predicted plasma concentration values of all studies were compared to the observed plasma concentrations in goodness-of-fit plots. In addition, the model performance was evaluated by comparison of predicted to observed areas under the plasma concentration-time curve (AUC) from the time of drug administration to the time of the last concentration measurement (AUC_{last}) and peak plasma concentration (C_{max}) values. As quantitative measures of the model performance, the mean relative deviation (MRD) of all predicted plasma concentrations (Equation S1) and the geometric mean fold error (GMFE) of all predicted AUC_{last} and C_{max} values (Equation S2) were calculated. MRD and GMFE values ≤ 2 characterize an adequate model performance.

$$MRD = 10^x \text{ with } x = \sqrt{\frac{1}{k}\sum_{i=1}^{k}(\log_{10} c_{\text{predicted},i} - \log_{10} c_{\text{observed},i})^2} \quad \text{(S1)}$$

where $c_{predicted,i}$ = predicted plasma concentration, $c_{observed,i}$ = corresponding observed plasma concentration, k = number of observed values.

$$GMFE = 10^x \text{ with } x = \frac{1}{m}\sum_{i=1}^{m} |\log_{10}\left(\frac{\text{predicted PK parameter}_i}{\text{observed PK parameter}_i}\right)| \tag{S2}$$

where predicted PK parameter$_i$ = predicted AUC_{last} or C_{max} value, observed PK parameter$_i$ = corresponding observed AUC_{last} or C_{max} value, m = number of studies.

1.2.2 PBPK model sensitivity analysis

Sensitivity of the final PBPK models to single parameters (local sensitivity analysis) was analyzed, measured as relative change of the area under the plasma concentration-time curve from 0 to 12 h ($AUC_{0\text{-}12}$) (probenecid) or area under the plasma concentration-time curve from 0 to 24 h ($AUC_{0\text{-}24}$) (furosemide) of the last administration interval for simulations of the highest recommended doses of probenecid (250 mg twice daily for 7 days, followed by 500 mg twice daily for 14 days) and furosemide (80 mg once daily for 14 days).
Sensitivity analysis was carried out with the implemented Sensitivity Analysis tool in PK-Sim® [4] using a relative perturbation of 1000% (variation range 10.0, maximum number of 9 steps). Parameters were included into the analysis if they had been optimized, are associated with optimized parameters or might have a strong impact due to calculation methods used in the model. Sensitivity to a parameter was calculated as the ratio of the relative change of the simulated $AUC_{0\text{-}12}$ or $AUC_{0\text{-}24}$ of the last administration interval to the relative variation of the parameter around its value used in the final model according to Equation S3:

$$S = \frac{\Delta AUC}{AUC} \cdot \frac{p}{\Delta p} \tag{S3}$$

where S = sensitivity of the $AUC_{0\text{-}12}$ or $AUC_{0\text{-}24}$ to the examined model parameter, ΔAUC = change of the simulated $AUC_{0\text{-}12}$ or $AUC_{0\text{-}24}$, AUC = simulated $AUC_{0\text{-}12}$ or $AUC_{0\text{-}24}$ with the original parameter value, Δp = change of the examined parameter value, p = original parameter value.
A sensitivity value of +0.5 signifies that a 100% increase of the examined parameter value causes a 50% increase of the simulated $AUC_{0\text{-}12}$ or $AUC_{0\text{-}24}$.

1.3 Drug-drug interaction (DDI) modeling

1.3.1 PBPK DDI modeling

As an additional means of model evaluation, the DDI performance of the developed models was assessed. To model the probenecid-furosemide DDI, inhibition of organic anion transporter 3 (OAT3), uridine 5'-diphospho-glucuronosyltransferase 1A9 (UGT1A9) and multidrug resistance-associated protein 4 (MRP4) by probenecid was implemented. To predict the probenecid-rifampicin DDI, inhibition of organic anion transporting polypeptide 1B1 (OATP1B1) by probenecid was incorporated (Figure S1.3.1). Mathematical implementation of the DDI processes is specified in Section 1.3.3. Inhibition constants characterizing the inhibition of OAT3, UGT1A9 (in-house measurement) and

OATP1B1 by probenecid were taken from in vitro experimental reports [9, 10]. To describe the competitive inhibition of MRP4 by probenecid, the corresponding inhibition constant was optimized during the furosemide PBPK model parameter identification using the clinical data of one of the probenecid-furosemide interaction studies [11] (see Table S5.2.1). The DDI parameter values are listed in the probenecid drug-dependent parameter table (Table S2.3.1).

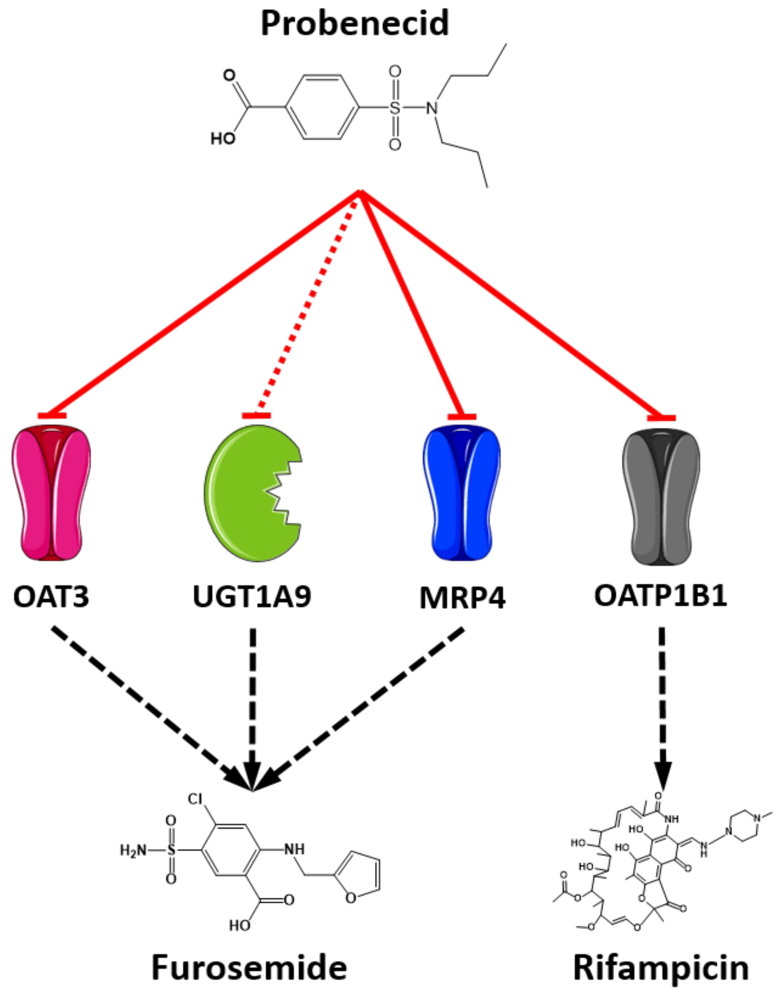

Figure S1.3.1: Probenecid DDIs. Schematic illustration of the modeled DDIs with probenecid as OAT3, UGT1A9, MRP4 and OATP1B1 perpetrator drug, furosemide as OAT3, UGT1A9 and MRP4 victim drug and rifampicin as OATP1B1 victim drug. The red solid lines indicate competitive inhibition, the red dotted line indicates non-competitive inhibition by probenecid. The black dashed lines indicate transport or metabolism. Drawings by Servier Medical Art, licensed under CC BY 3.0. **MRP4:** multidrug resistance-asociated protein 4, **OAT3:** organic anion transporter 3, **OATP1B1:** organic anion transporting polypeptide 1B1, **UGT1A9:** uridine 5'-diphospho-glucuronosyltransferase 1A9.

1.3.2 PBPK DDI performance evaluation

All DDI predictions were evaluated by comparison of predicted versus observed victim drug plasma concentration-time profiles alone and during co-administration, DDI AUC_{last} ratios and DDI C_{max} ratios (Equation S4).

$$\text{DDI PK parameter ratio} = \frac{\text{PK parameter}_{\text{victim drug during co-administration}}}{\text{PK parameter}_{\text{victim drug alone}}} \tag{S4}$$

where PK parameter = AUC_{last} or C_{max}.

As a quantitative measure of the DDI prediction accuracy, GMFEs of the predicted DDI AUC_{last} ratios and DDI C_{max} ratios were calculated according to S2. Predicted and observed DDI AUC_{last} ratios and DDI C_{max} ratios and corresponding GMFEs are summarized in Tables S5.4.1 and S6.4.1.

1.3.3 Mathematical implementation of DDIs

The mathematical implementation of DDIs is described in the following section. All presented interaction processes and the corresponding formulas are implemented in PK-Sim® and are documented in the Open Systems Pharmacology Suite Manual [4].

Competitive inhibition

Competitive inhibitors reversibly bind to the active site of an enzyme or transporter and compete with the substrate for binding. Competitive inhibition can be overcome by high substrate concentrations (concentration-dependency); therefore, the maximum reaction velocity (v_{max}) remains unaffected, while the Michaelis-Menten constant (K_M) is increased (Michaelis-Menten constant in the presence of inhibitor ($K_{M,app}$), Equation S5). The reaction velocity (v) during co-administration of substrate and competitive inhibitor is described by Equation S6 [4]:

$$K_{M,app} = K_M \cdot \left(1 + \frac{[I]}{K_i}\right) \tag{S5}$$

$$v = \frac{v_{max} \cdot [S]}{K_{M,app} + [S]} \tag{S6}$$

where $K_{M,app}$ = Michaelis-Menten constant in the presence of inhibitor, K_M = Michaelis-Menten constant, $[I]$ = free inhibitor concentration, K_i = dissociation constant of the inhibitor-enzyme/transporter complex, v = reaction velocity, v_{max} = maximum reaction velocity, $[S]$ = free substrate concentration.

Non-competitive inhibition

Non-competitive inhibitors reversibly bind to a site different from the active site. This reduces the activity of the enzyme or transporter, but does not affect the substrate binding. The inhibitor binds to the free enzyme or to the enzyme-substrate complex with the same inhibition constant (K_i) and the substrate can still bind to the enzyme-inhibitor complex. In the case of non-competitive inhibition, the v_{max} is reduced (maximum reaction velocity in the presence of inhibitor ($v_{max,app}$), Equation S7), while the K_M remains unaffected. The reaction velocity (v) during co-administration of substrate and non-competitive inhibitor is described by Equation S8 [4]:

$$v_{max,app} = \frac{v_{max}}{1 + \frac{[I]}{K_i}} \tag{S7}$$

$$v = \frac{v_{max,app} \cdot [S]}{K_M + [S]} \tag{S8}$$

where $v_{max,app}$ = maximum reaction velocity in the presence of inhibitor, v_{max} = maximum reaction velocity, $[I]$ = free inhibitor concentration, K_i = dissociation constant of the enzyme/transporter-substrate-inhibitor complex, v = reaction velocity, $[S]$ = free substrate concentration, K_M = Michaelis-Menten constant.

2 PBPK modeling of probenecid

2.1 PBPK model development

Probenecid is a uricosuric agent to treat gout or hyperuricemia and is also used to increase plasma concentrations of antibiotics [12]. The recommended dose to treat hyperuricemia is 250 mg twice daily for one week followed by 500 mg twice daily as maintenance dose [13]. Probenecid is highly bound to plasma proteins (fraction unbound in plasma (f_u) = 12%) [14]. The volume of distribution (V_D) after oral administration is 9.5 to 11.4 l [15]. Metabolic pathways include glucuronidation, hydroxylation and demethylation [16, 17]. The pharmacokinetics (PK) of orally administered single doses are nonlinear, due to one or more saturable metabolic pathways [15, 18]. About 72% to 86% of an orally administered dose are excreted in the urine. Out of this fraction, only 0.3% to 5% are excreted unchanged [14]. The FDA lists probenecid as a potent clinical OAT1/OAT3 inhibitor [19].
The probenecid PBPK model was developed using 27 different clinical studies including intravenous (single dose) and oral (single- and multiple dose) administration. Please refer to Table S2.2.1 for the complete list of clinical studies used in the presented analysis. In addition, five studies reported fraction excreted unchanged in urine profiles following oral administration.
The final probenecid PBPK model applies uptake into kidney cells via OAT3, glucuronidation mainly in the renal cells by UGT1A9, glomerular filtration and tubular reabsorption, which was modeled as a reduction of the glomerular filtration rate (GFR fraction < 1). Due to our current lack of knowledge regarding transporters that may contribute to probenecid reabsorption, the GFR fraction was optimized to 0.03. This reduced GFR fraction substitutes for the implementation of active reabsorption processes of probenecid [4] and correctly captures the low probenecid fraction excreted unchanged in urine. In the clinical studies conducted by Vree et al. [14, 20], the probenecid tablets were broken in half prior to oral administration. The corresponding plasma concentration-time profiles display an earlier time to peak plasma concentration (T_{max}) of 1.6 h compared to the other clinical studies with a T_{max} of 3.3 h. Given the low solubility of probenecid it is possible that the broken tablets show a different dissolution behavior, resulting in faster release and absorption. Therefore, a different dissolution profile was used to describe the studies by Vree et al. [14, 20]. The parameters to model the two different dissolution profiles and the drug-dependent parameters of the final probenecid PBPK model are summarized in Table S2.3.1.
Population predicted compared to observed plasma concentration-time profiles of all 27 clinical studies included in this analysis are shown in semilogarithmic (Figure S2.4.1) and linear plots (Figure S2.4.2). Individual predicted compared to observed plasma concentration-time profiles of all 27 clinical studies included in this analysis are shown in semilogarithmic (Figure S2.4.3) and linear plots (Figure S2.4.4). Population predicted compared to observed fraction excreted unchanged in urine profiles are shown in Figure S2.4.5. Individual predicted compared to observed fraction excreted unchanged in urine profiles are shown in Figure S2.4.6. Figure S2.5.1 shows predicted compared to observed plasma concentration values in a goodness-of-fit plot. Table S2.5.1 lists the MRD values of all 27 studies. The correlation of predicted compared to observed probenecid AUC_{last} and C_{max} values is presented in Figure S2.5.2, further demonstrating the good model performance with 27/27 predicted AUC_{last} and 18/18 predicted C_{max} values within 2-fold of the observed data. The individual values and mean GMFE values and ranges are listed in Table S2.5.2.
The sensitivity analysis results of a simulation of 500 mg probenecid twice daily are illustrated in Figure S2.5.3. Applying a threshold of 0.5, the probenecid model is sensitive to the values of the

UGT1A9 catalytic rate constant (optimized) and Michaelis-Menten constant (literature value), the probenecid fraction unbound in plasma (literature value), the OAT3 catalytic rate constant (optimized) and the probenecid lipophilicity (optimized).

2.2 Clinical studies

Table S2.2.1: Probenecid study table

Dose [mg]	Route	n	Age [years]	Weight [kg]	Height [cm]	Females [%]	Dataset	Reference
464.20[a]	iv (-), sd	3[b]	-	-	-	-	test/training[c]	Dayton 1963 [21]
500	iv (15 min), sd	6	23-36	52-67	-	100	training	Emanuelsson 1987 [15]
1860[a]	iv (-), sd	5[b]	-	-	-	-	test/training[d]	Dayton 1963 [21]
250	po (tab), sd	1	40	-	-	0	training[e]	Vree 1992 [14]
500	po (tab), sd	6	23-36	52-67	-	100	test	Emanuelsson 1987 [15]
500	po (tab), sd	5	20-39	53-86	-	0	training	Selen 1982 [18]
500	po (tab), sd	1	40	-	-	0	test	Vree 1992 [14]
500/1000	po (tab), md[f]	12	29 (21-38)	67 ± 12	175	50	test	Landersdorfer 2010 [22]
1000	po (tab), sd	6	23-36	52-67	-	100	test	Emanuelsson 1987 [15]
1000	po (tab), sd	5	20-39	53-86	-	0	training	Selen 1982 [18]
1000	po (tab), sd	14	26 (18-45)	63 ± 7	-	0	test	Shen 2019 [23]
1000	po (tab), sd	1	40	-	-	0	test	Vree 1992 [14]
1000	po (tab), sd	1	-	-	-	-	test	Vree 1993 [20]
1000	po (-), bid	4	21-33	65-77	-	0	test	Smith 1980 [24]
1000	po (tab), bid	14	33 (21-51)	79 (62-95)	180 (169-191)	0	training	Wiebe 2020 [11]
1000/500/250	po (tab), md[g]	17	-	69 ± 13	173 ± 10	47	training	Landersdorfer 2009 [25]
1500	po (tab), sd	1	40	-	-	0	training[e]	Vree 1992 [14]
2000	po (tab), sd	2[b]	-	-	-	-	test	Dayton 1963 [21]
2000	po (tab), sd	6	23-36	52-67	-	100	training	Emanuelsson 1987 [15]
2000	po (tab), sd	5	20-39	53-86	-	0	training	Selen 1982 [18]

bid: twice daily, **iv:** intravenous, **md:** multiple dose, **n:** number of individuals studied, **po:** oral, **route:** route of administration, **sd:** single dose, **tab:** tablet, **test:** test dataset (model evaluation), **training:** training dataset (parameter optimization). Values are means ± standard deviation or ranges.

[a] administration as 500 mg or 2000 mg sodium probenecid

[b] individual profiles reported

[c] plasma concentration-time profile of Subject D was assigned to training dataset

[d] plasma concentration-time profile of Subject E was assigned to training dataset

[e] plasma concentration-time profiles were used to optimize Weibull parameters for studies of Vree 1992 and Vree 1993 [14, 20]

[f] probenecid administration: 500 mg (0 h), 1000 mg (8 h) and 500 mg (14.5, 20.5, 26.5 h)

[g] probenecid administration: 1000 mg (0, 8 h), 250 mg (16, 24 h) and 500 mg (34, 46, 58, 70 h)

2.3 Drug-dependent parameters

Table S2.3.1: Drug-dependent parameters of the probenecid PBPK model

Parameter	Value	Unit	Source	Literature	Reference	Description
MW	285.36	g/mol	Literature	285.36	[26]	Molecular weight
pKa (acid)	3.70	-	Literature	3.01, 3.70	[27, 28]	Acid dissociation constant
Solubility (HIF)	0.74	mg/ml	Literature	0.74 (HIF), 1.29 (pH 6.50), 1.63 (FaSSIF)	[29]	Solubility
logP	1.34	-	Optimized	- 0.52, -0.23, 0.13, 3.21, 3.70	[21, 27, 28, 30]	Lipophilicity
fu	11.70	%	Literature	6.20, 7.56, 11.70	[14, 20, 23]	Fraction unbound in plasma
OAT3 K_M	12.18	µmol/l	Optimized	-	-	OAT3 Michaelis-Menten constant
OAT3 k_{cat}	1966.57	1/min	Optimized	-	-	OAT3 transport rate constant
UGT1A9 K_M	198.30	µmol/l	Literature	198.30	[16]	UGT1A9 Michaelis-Menten constant
UGT1A9 k_{cat}	74.92	1/min	Optimized	-	-	UGT1A9 catalytic rate constant
GFR fraction	0.03	-	Optimized	-	[14, 20]	Fraction of filtered drug in the urine
EHC continuous fraction	1.00	-	Assumed	-	-	Fraction of bile continually released
K_i OAT3	5.41	µmol/l	Literature	5.41	[9]	Conc. for half-maximal competitive inhibition
K_i UGT1A9	242.00	µmol/l	Measured[a]	-	-	Conc. for half-maximal non-competitive inhibition
K_i MRP4	87.40	µmol/l	Optimized	-	[11]	Conc. for half-maximal competitive inhibition
K_i OATP1B1	39.80	µmol/l	Literature	39.80	[10]	Conc. for half-maximal competitive inhibition
Partition coefficients	Diverse	-	Calculated	PK-Sim	[4]	Cell to plasma partition coefficients
Cellular permeability	1.17E-3	cm/min	Calculated	CdS norm	[4]	Permeability into the cellular space
Intestinal permeability (trans.)	3.97E-4	cm/min	Optimized	3.12E-6	Calculated	Transcellular intestinal permeability
Tablet fasted Weibull shape	0.58	-	Optimized	-	[11, 15, 18, 25]	Dissolution profile shape
Tablet fasted Weibull time	44.25	min	Optimized	-	[11, 15, 18, 25]	Dissolution time (50% dissolved)
Tablet fasted Weibull shape[b]	0.15	-	Optimized	-	[14]	Dissolution profile shape
Tablet fasted Weibull time[b]	0.08	min	Optimized	-	[14]	Dissolution time (50% dissolved)

CdS norm: charge-dependent Schmitt normalized to PK-Sim calculation method, **conc.:** concentration, **EHC:** enterohepatic circulation, **FaSSIF:** fasted state simulated intestinal fluid, **GFR:** glomerular filtration rate, **HIF:** human intestinal fluid, **MRP4:** multidrug resistance-associated protein 4, **OAT3:** organic anion transporter 3, **PK-Sim:** PK-Sim standard calculation method, **trans.:** transcellular, **UGT1A9:** uridine 5'-diphospho-glucuronosyltransferase 1A9.

[a] The probenecid UGT1A9 K_i was determined using Corning® Supersomes™ Human UGT1A9 with furosemide as the substrate (18-72 µmol/l) and varied probenecid (0-3000 µmol/l) concentrations

[b] Weibull function used for clinical studies conducted by Vree 1992 and Vree 1993 [14, 20]

2.4 Profiles

2.4.1 Semilogarithmic plots - Plasma - Population predictions

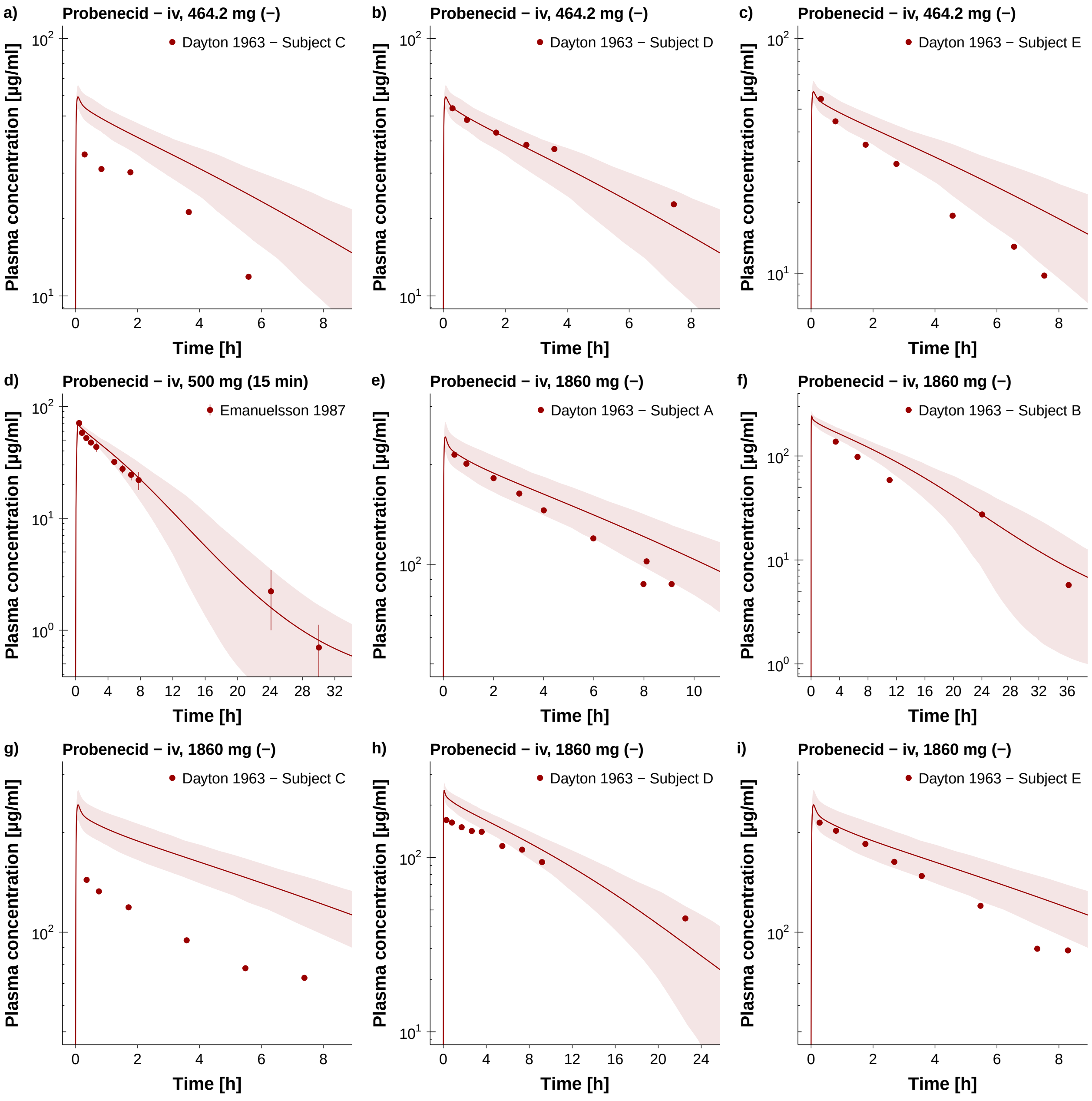

Figure S2.4.1: Probenecid plasma concentration-time profiles. Population predictions of probenecid plasma concentration-time profiles compared to observed data. Observed data are shown as dots $\pm$ standard deviation. Population simulation arithmetic means are shown as lines; the shaded areas illustrate the predicted population variation ($Q_{16} - Q_{84}$). Details on dosing regimens, study populations and literature references are summarized in Table S2.2.1.

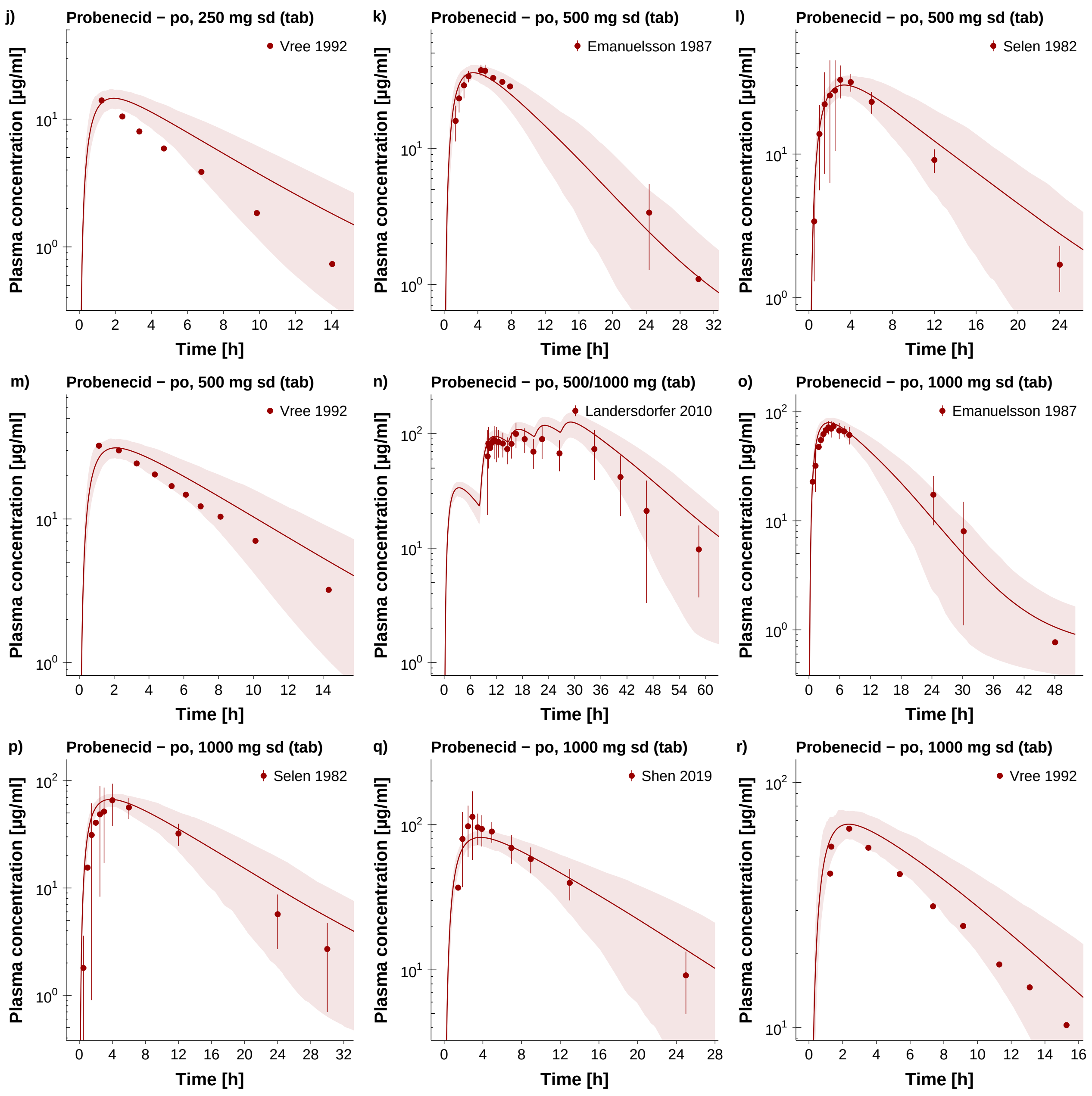

Figure S2.4.1: Probenecid plasma concentration-time profiles. Population predictions of probenecid plasma concentration-time profiles compared to observed data. Observed data are shown as dots ± standard deviation. Population simulation arithmetic means are shown as lines; the shaded areas illustrate the predicted population variation ($Q_{16} - Q_{84}$). Details on dosing regimens, study populations and literature references are summarized in Table S2.2.1.

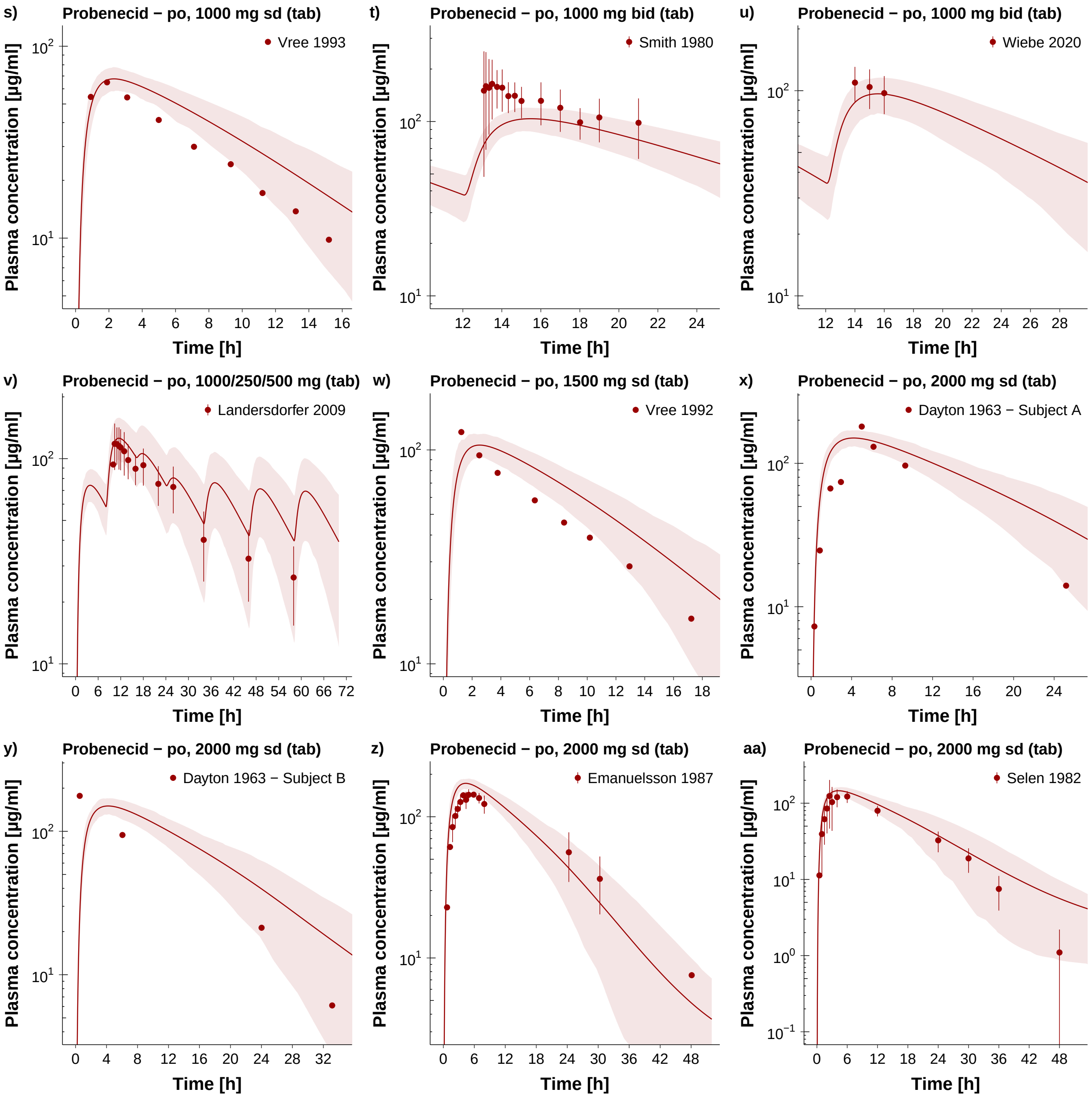

Figure S2.4.1: Probenecid plasma concentration-time profiles. Population predictions of probenecid plasma concentration-time profiles compared to observed data. Observed data are shown as dots $\pm$ standard deviation. Population simulation arithmetic means are shown as lines; the shaded areas illustrate the predicted population variation ($Q_{16} - Q_{84}$). Details on dosing regimens, study populations and literature references are summarized in Table S2.2.1.

2.4.2 Linear plots - Plasma - Population predictions

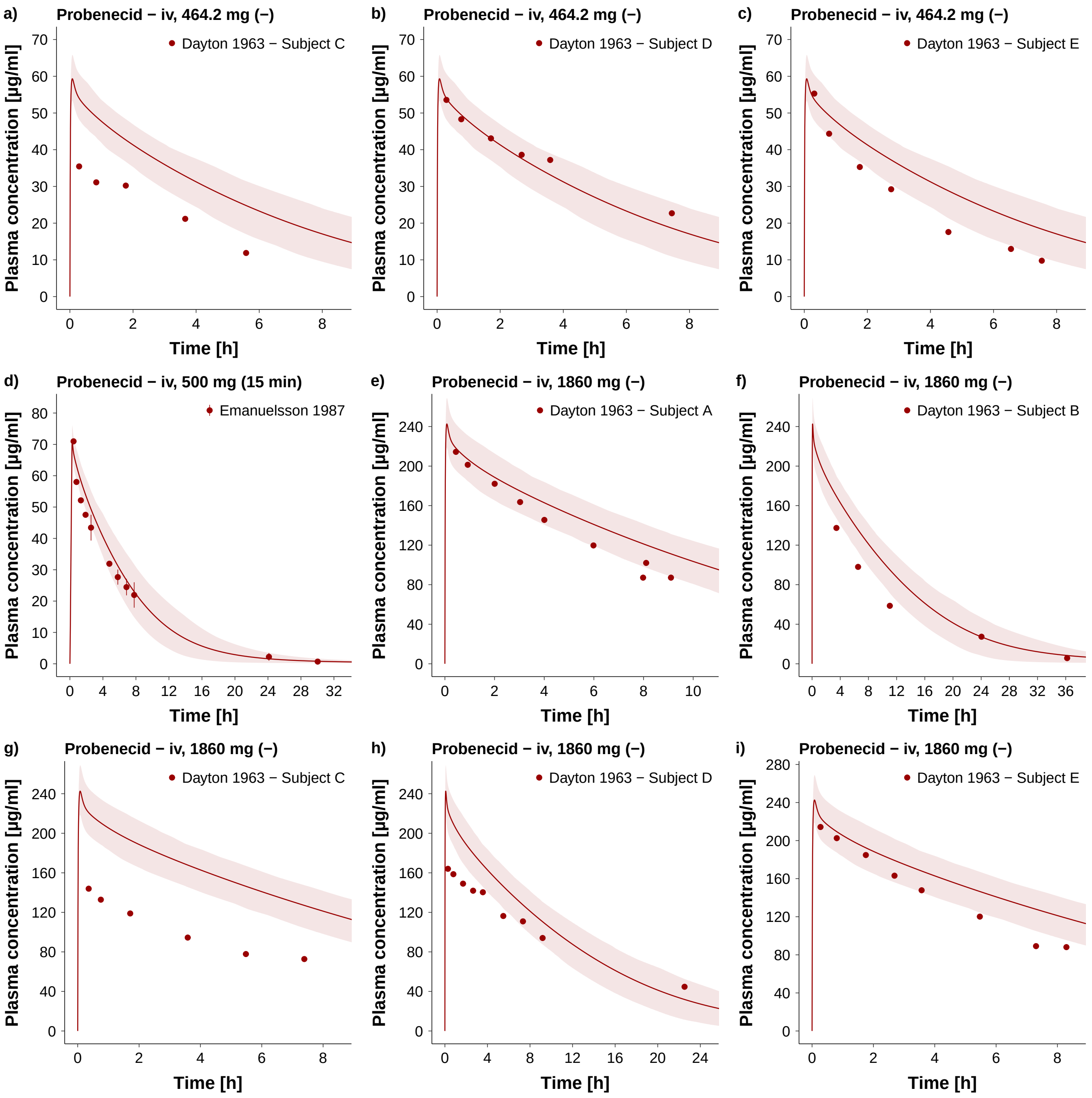

Figure S2.4.2: Probenecid plasma concentration-time profiles. Population predictions of probenecid plasma concentration-time profiles compared to observed data. Observed data are shown as dots $\pm$ standard deviation. Population simulation arithmetic means are shown as lines; the shaded areas illustrate the predicted population variation ($Q_{16} - Q_{84}$). Details on dosing regimens, study populations and literature references are summarized in Table S2.2.1.

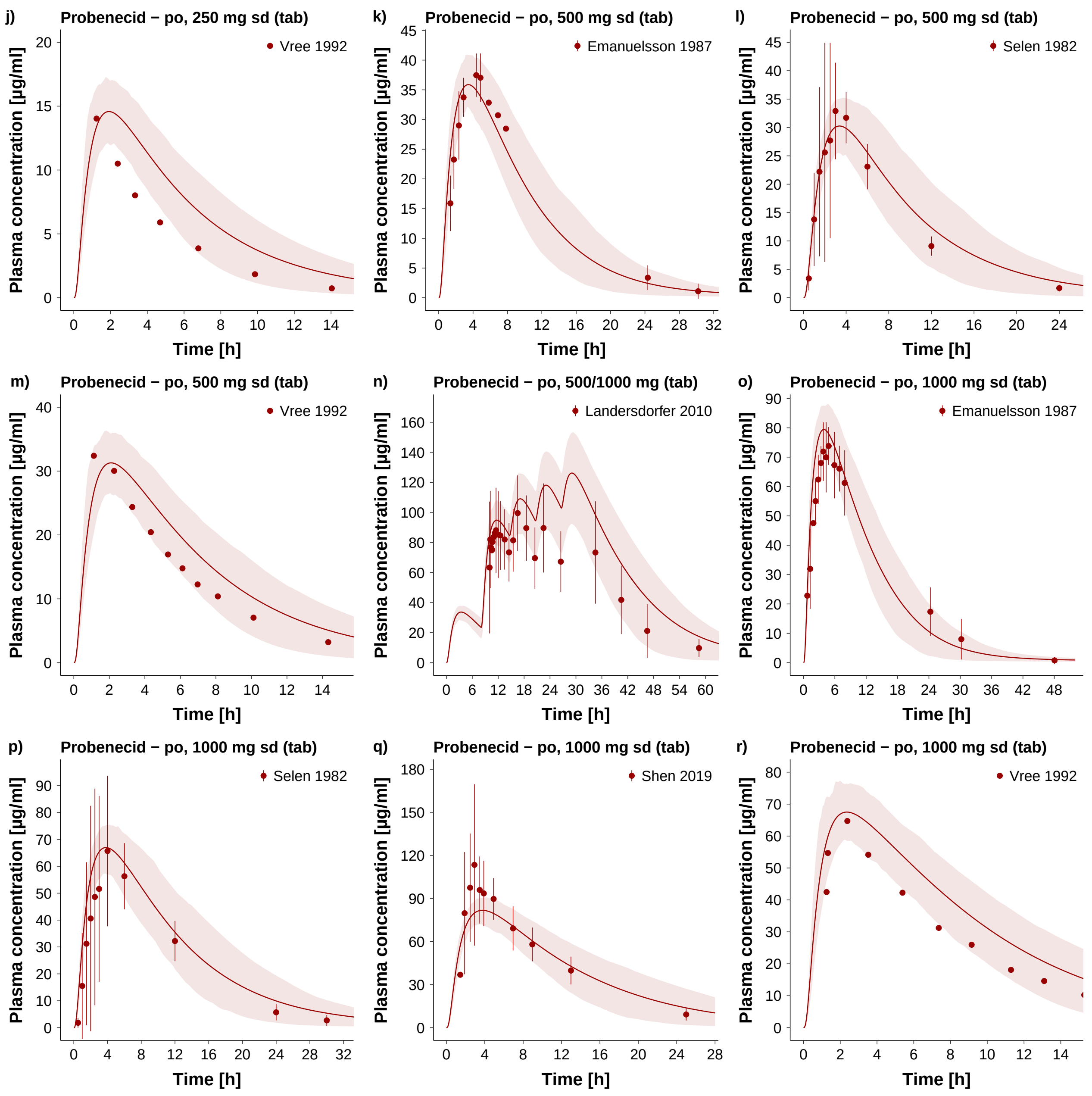

Figure S2.4.2: Probenecid plasma concentration-time profiles. Population predictions of probenecid plasma concentration-time profiles compared to observed data. Observed data are shown as dots $\pm$ standard deviation. Population simulation arithmetic means are shown as lines; the shaded areas illustrate the predicted population variation ($Q_{16} - Q_{84}$). Details on dosing regimens, study populations and literature references are summarized in Table S2.2.1.

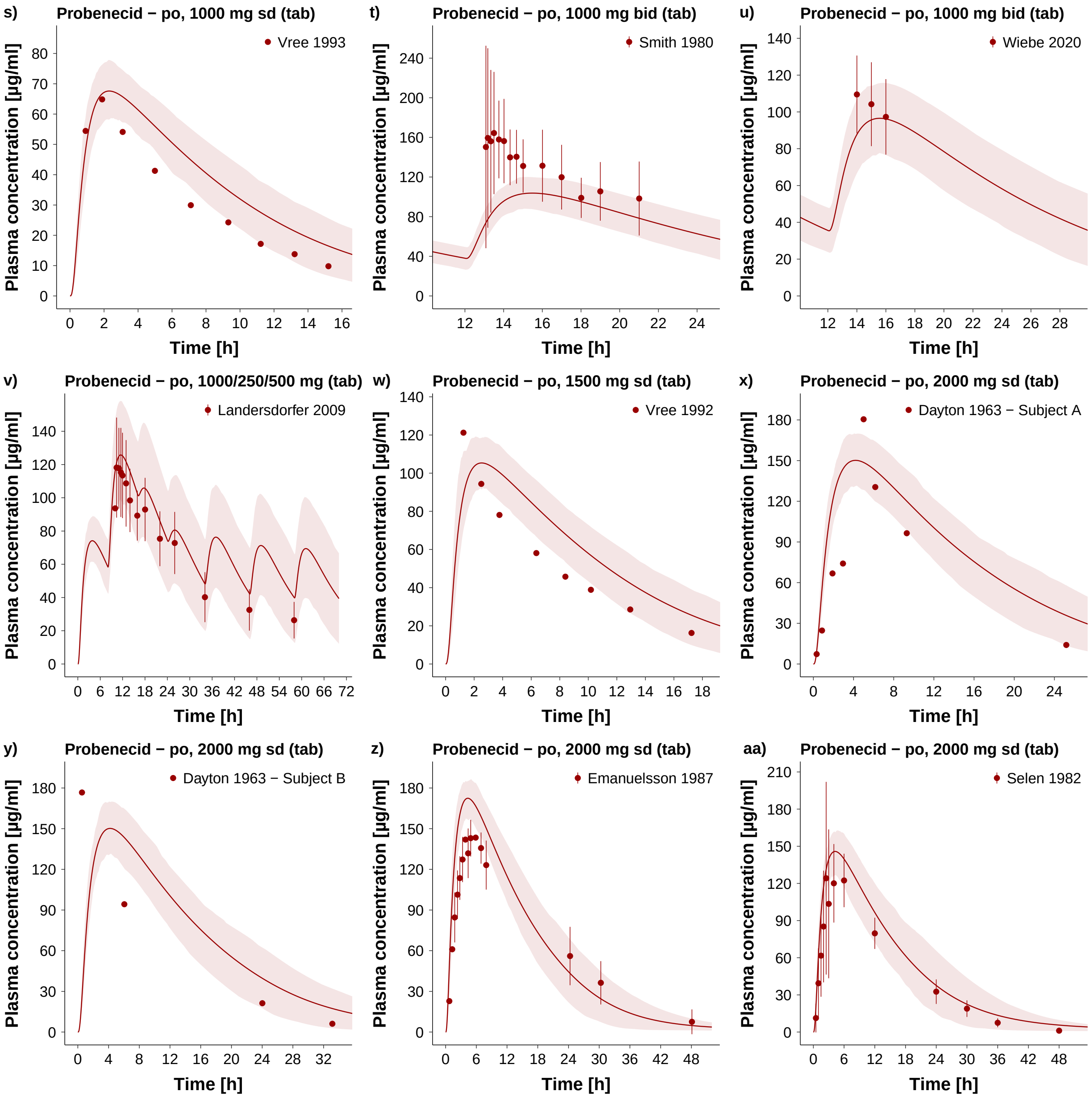

Figure S2.4.2: Probenecid plasma concentration-time profiles. Population predictions of probenecid plasma concentration-time profiles compared to observed data. Observed data are shown as dots ± standard deviation. Population simulation arithmetic means are shown as lines; the shaded areas illustrate the predicted population variation ($Q_{16} - Q_{84}$). Details on dosing regimens, study populations and literature references are summarized in Table S2.2.1.

2.4.3 Semilogarithmic plots - Plasma - Individual predictions

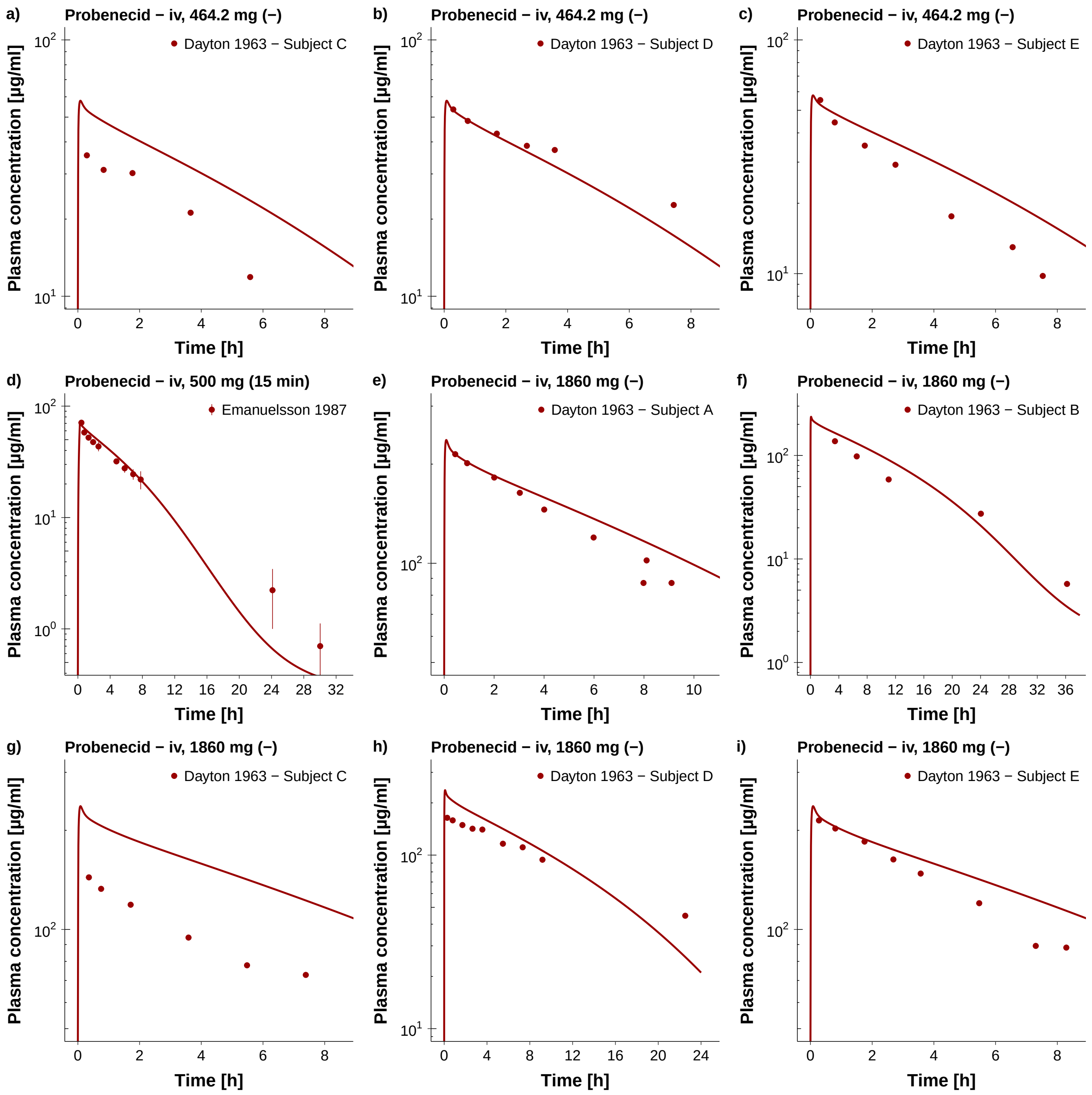

Figure S2.4.3: Probenecid plasma concentration-time profiles. Individual predictions of probenecid plasma concentration-time profiles compared to observed data. Observed data are shown as dots ± standard deviation. Simulations are shown as lines. Details on dosing regimens, study populations and literature references are summarized in Table S2.2.1.

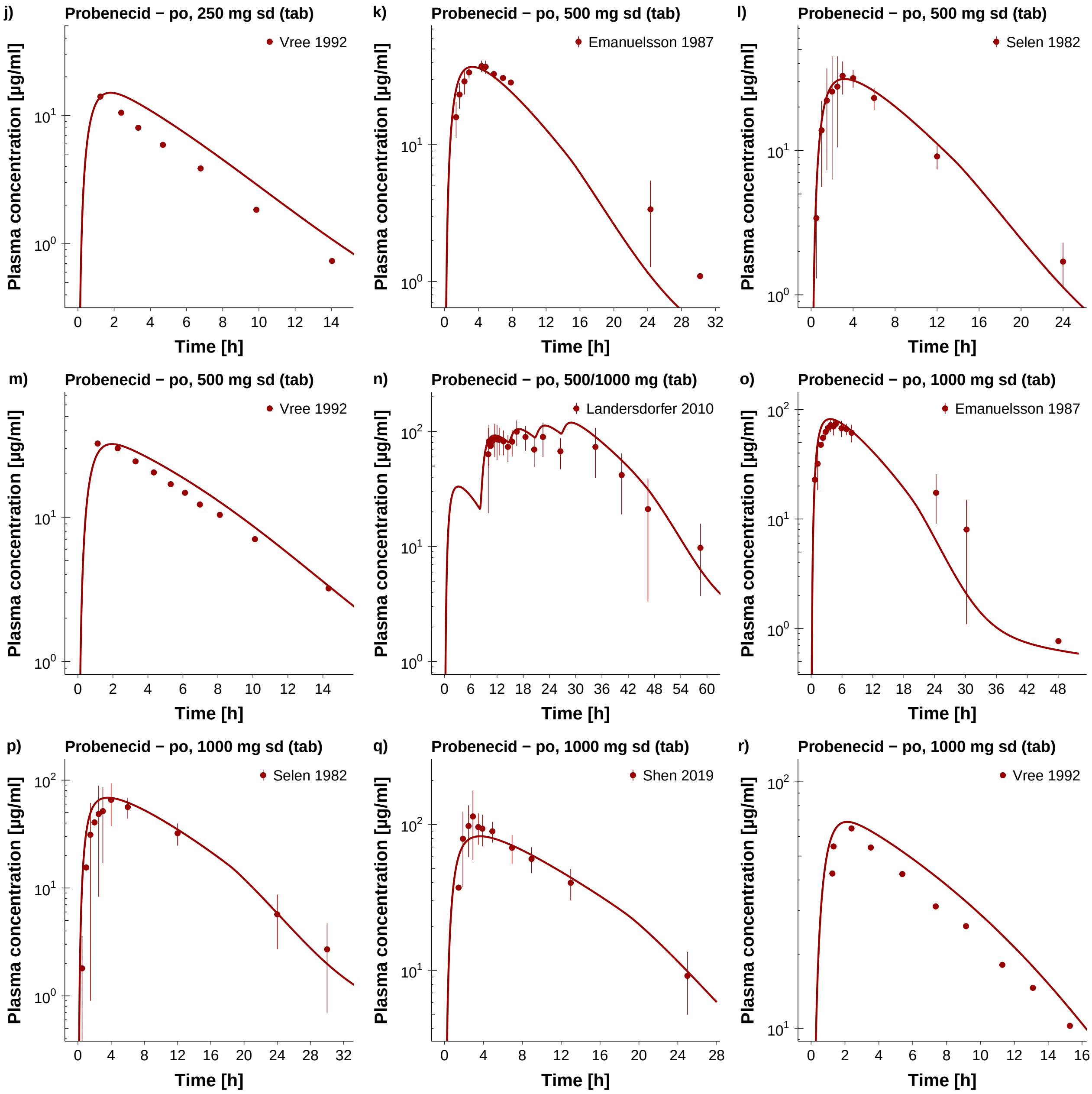

Figure S2.4.3: Probenecid plasma concentration-time profiles. Individual predictions of probenecid plasma concentration-time profiles compared to observed data. Observed data are shown as dots ± standard deviation. Simulations are shown as lines. Details on dosing regimens, study populations and literature references are summarized in Table S2.2.1.

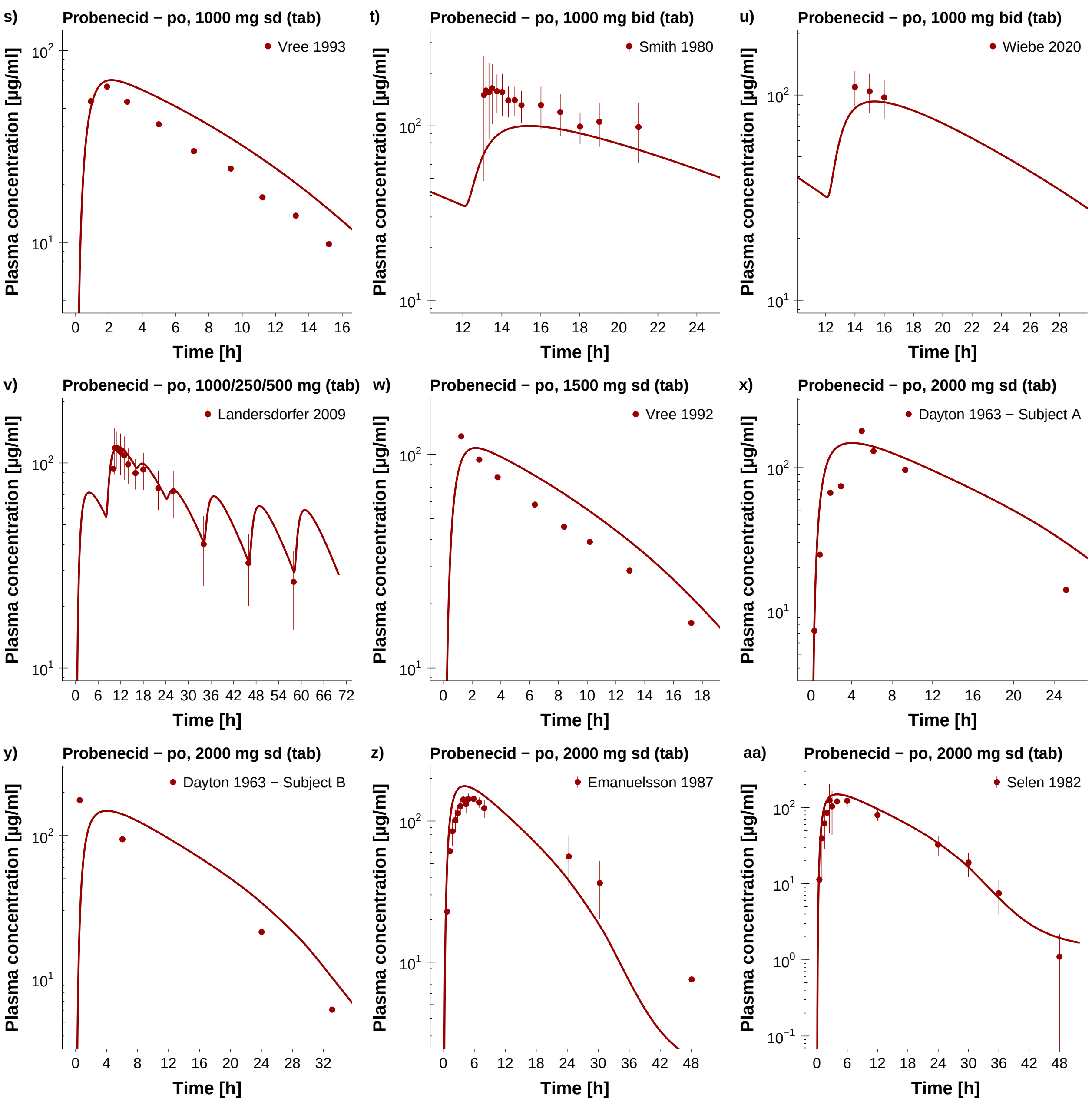

Figure S2.4.3: Probenecid plasma concentration-time profiles. Individual predictions of probenecid plasma concentration-time profiles compared to observed data. Observed data are shown as dots $\pm$ standard deviation. Simulations are shown as lines. Details on dosing regimens, study populations and literature references are summarized in Table S2.2.1.

2.4.4 Linear plots - Plasma - Individual predictions

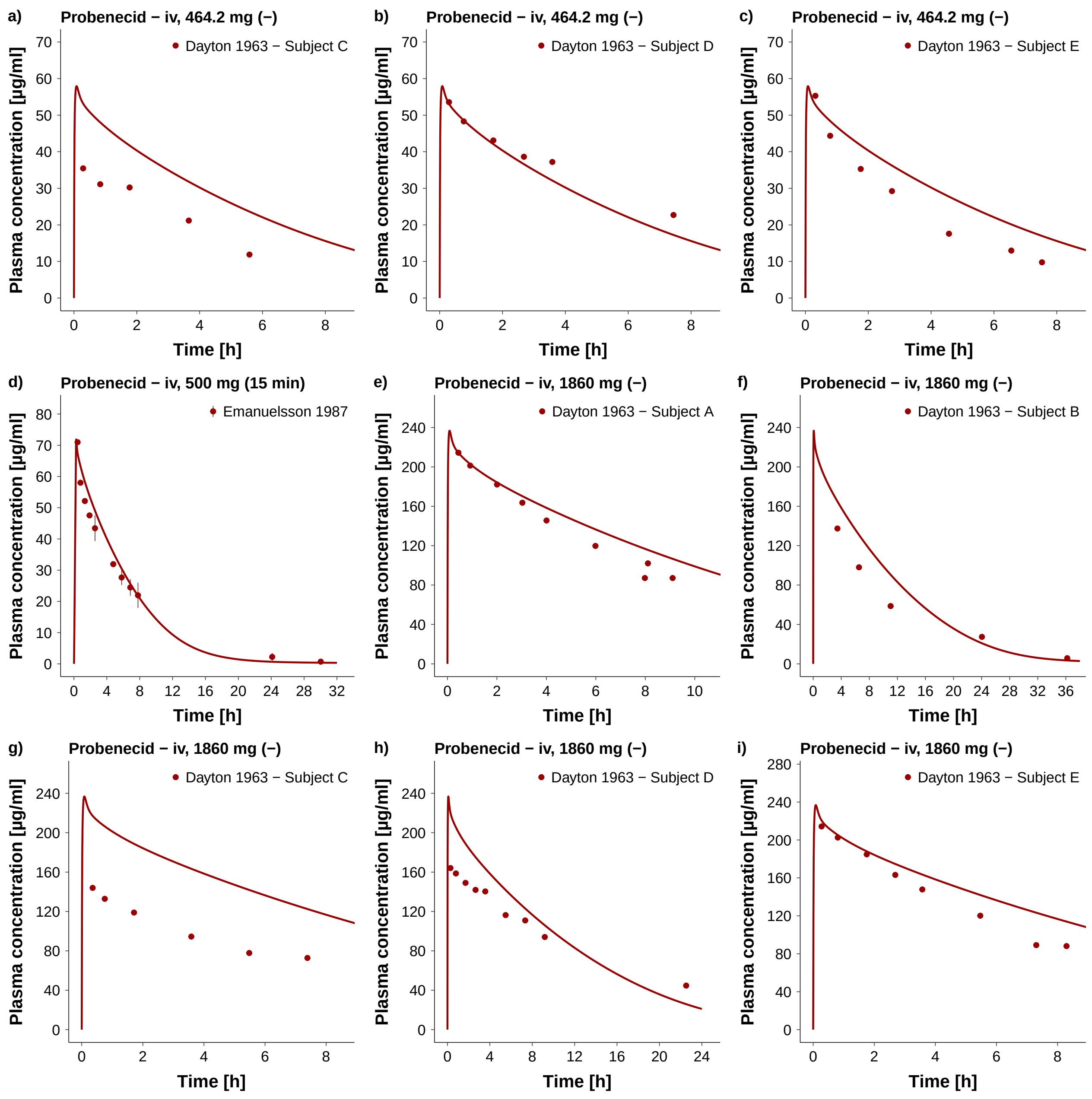

Figure S2.4.4: Probenecid plasma concentration-time profiles. Individual predictions of probenecid plasma concentration-time profiles compared to observed data. Observed data are shown as dots $\pm$ standard deviation. Simulations are shown as lines. Details on dosing regimens, study populations and literature references are summarized in Table S2.2.1..

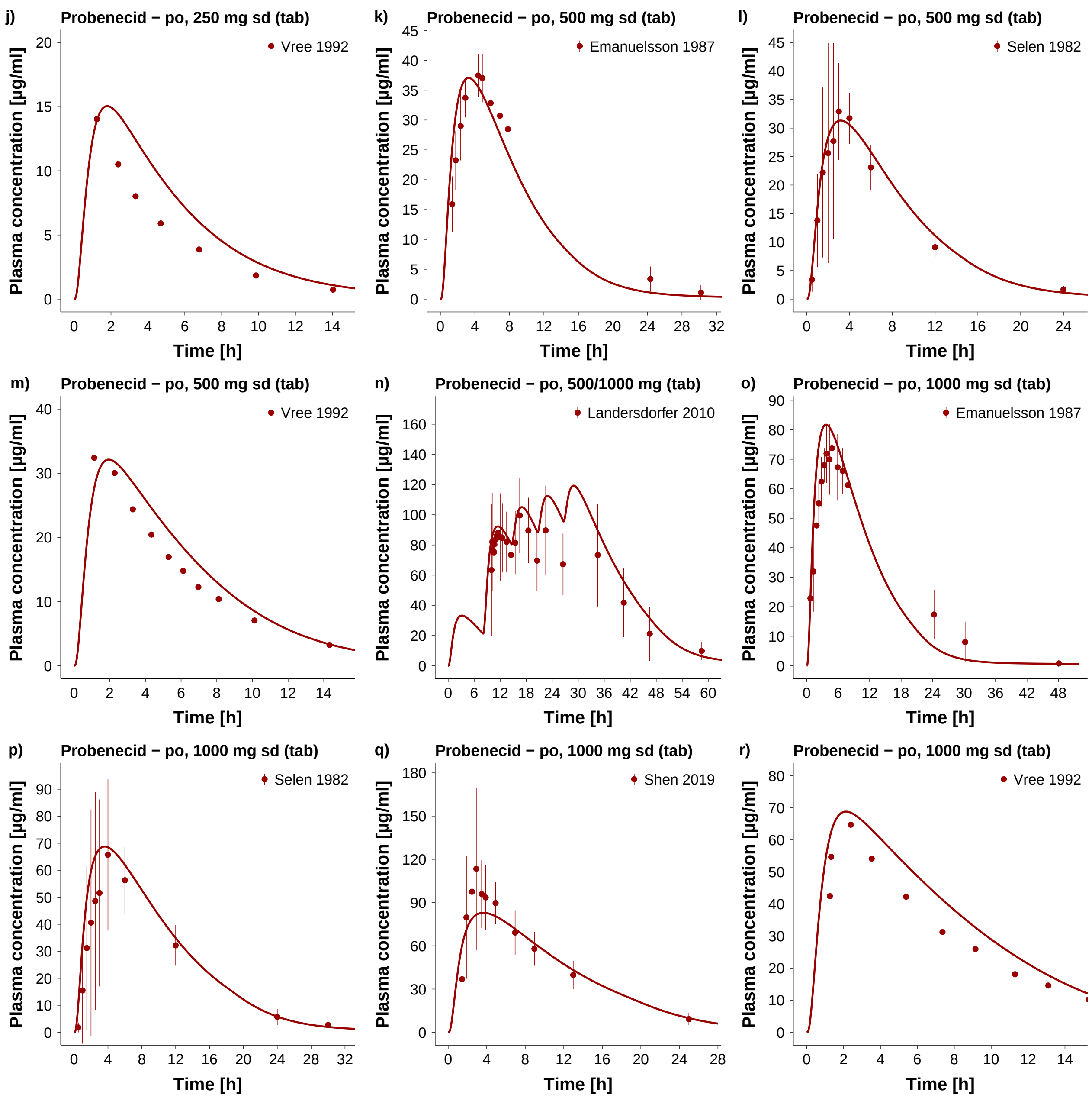

Figure S2.4.4: Probenecid plasma concentration-time profiles. Individual predictions of probenecid plasma concentration-time profiles compared to observed data. Observed data are shown as dots ± standard deviation. Simulations are shown as lines. Details on dosing regimens, study populations and literature references are summarized in Table S2.2.1..

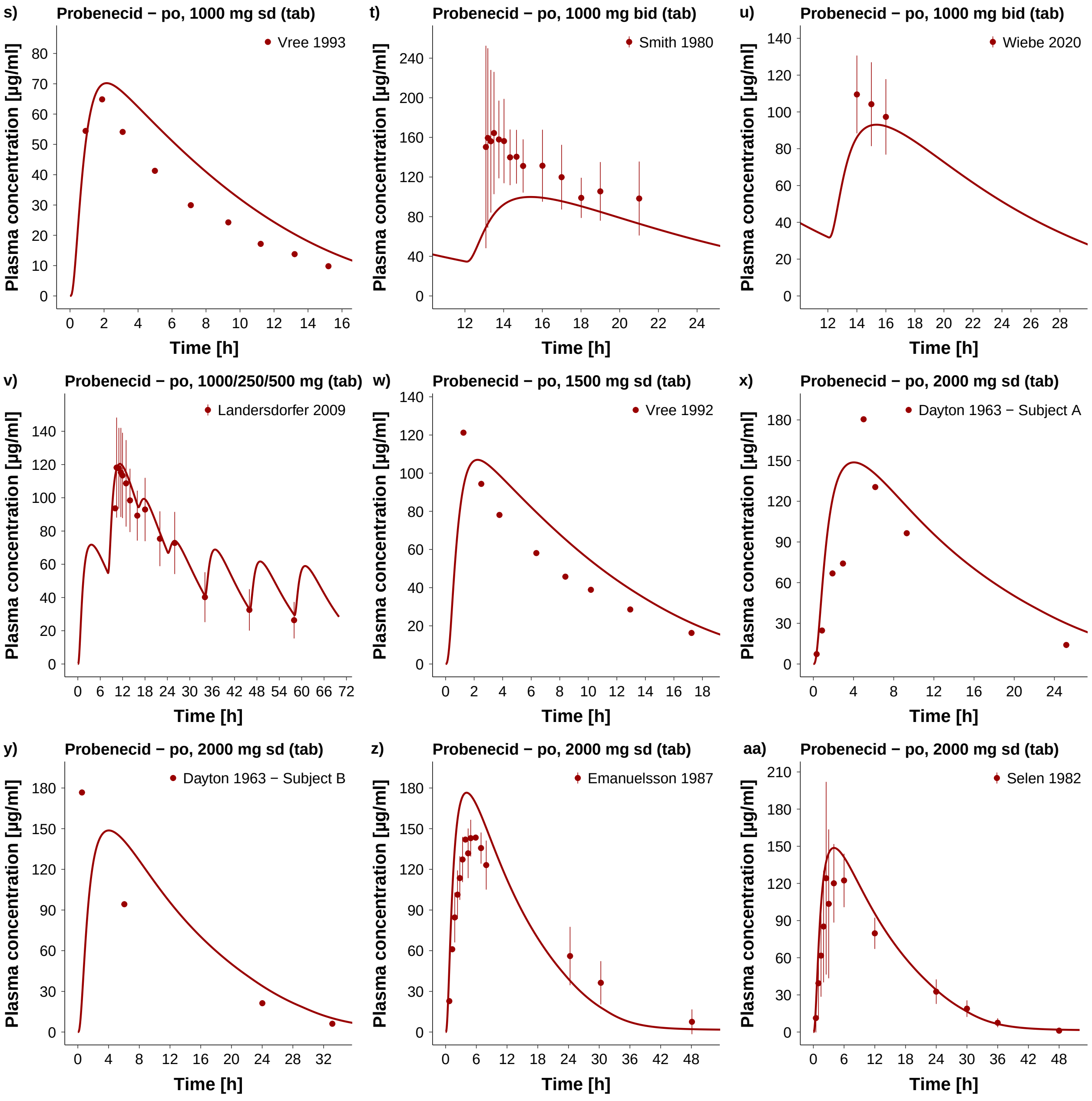

Figure S2.4.4: Probenecid plasma concentration-time profiles. Individual predictions of probenecid plasma concentration-time profiles compared to observed data. Observed data are shown as dots ± standard deviation. Simulations are shown as lines. Details on dosing regimens, study populations and literature references are summarized in Table S2.2.1.

2.4.5 Linear plots - Fraction excreted unchanged in urine - Population predictions

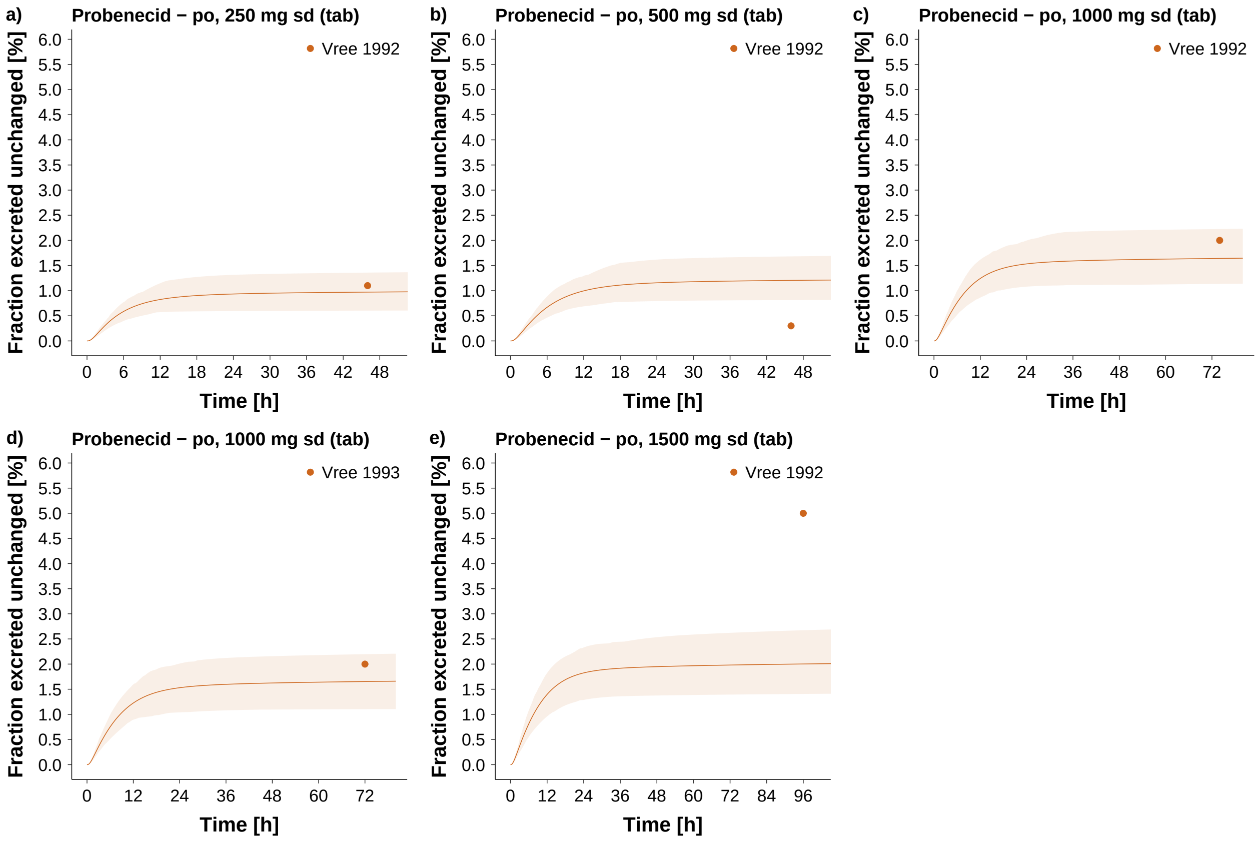

Figure S2.4.5: Probenecid fraction excreted unchanged in urine profiles. Population predictions of probenecid fraction excreted unchanged in urine profiles compared to observed data. Observed data are shown as dots. Population simulation arithmetic means are shown as lines; the shaded areas illustrate the corresponding predicted population variation ($Q_{16} - Q_{84}$). Details on dosing regimens, study populations and literature references are summarized in Table S2.2.1.

2.4.6 Linear plots - Fraction excreted unchanged in urine - Individual predictions

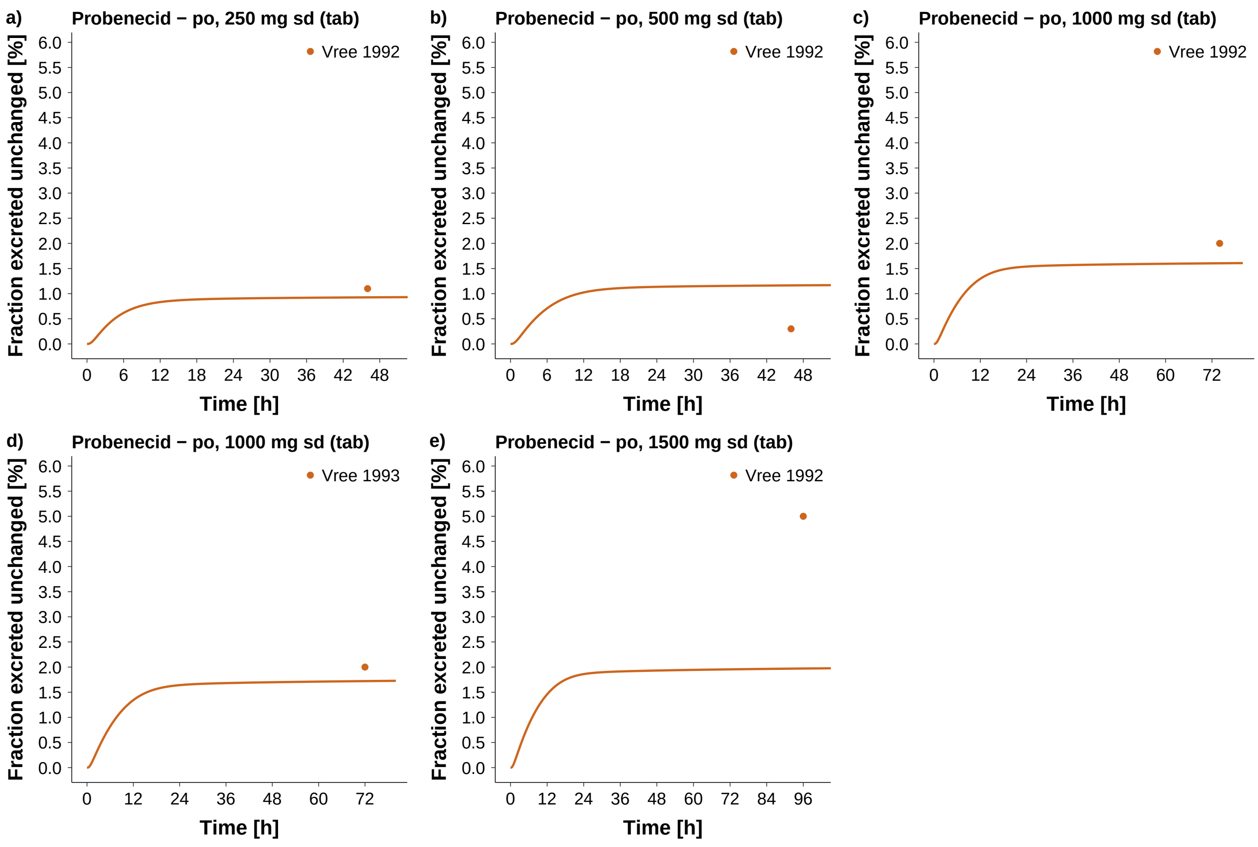

Figure S2.4.6: Probenecid fraction excreted unchanged in urine profiles. Individual predictions of probenecid fraction excreted unchanged in urine profiles compared to observed data. Observed data are shown as dots. Simulations are shown as lines. Details on dosing regimens, study populations and literature references are summarized in Table S2.2.1.

2.5 Probenecid PBPK model evaluation

2.5.1 Plasma concentration goodness-of-fit plot

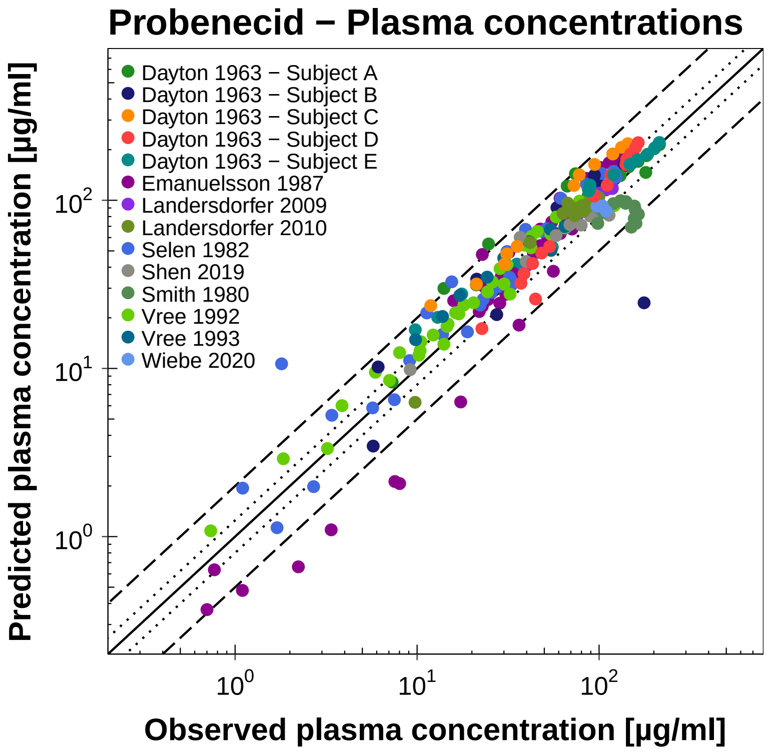

Figure S2.5.1: Probenecid plasma concentrations. Predicted compared to observed probenecid plasma concentration values of all analyzed clinical studies. The solid line marks the line of identity. The dotted lines indicate 1.25-fold, the dashed lines indicate 2-fold deviation. Details on dosing regimens, study populations and literature references are summarized in Table S2.2.1.

2.5.2 Mean relative deviation (MRD) of predicted plasma concentrations

Table S2.5.1: MRD values of predicted probenecid plasma concentrations

Route	Dose [mg]	MRD	Reference
Intravenous			
iv (-), sd	464.2	1.60	Dayton 1963 – Subject C [21]
iv (-), sd	464.2	1.14	Dayton 1963 – Subject D [21]
iv (-), sd	464.2	1.40	Dayton 1963 – Subject E [21]
iv (15 min), sd	500	1.53	Emanuelsson 1987 [15]
iv (-), sd	1860	1.15	Dayton 1963 – Subject A [21]
iv (-), sd	1860	1.43	Dayton 1963 – Subject B [21]
iv (-), sd	1860	1.65	Dayton 1963 –Subject C [21]
iv (-), sd	1860	1.31	Dayton 1963 – Subject D [21]
iv (-), sd	1860	1.18	Dayton 1963 – Subject E [21]
**MRD**		**1.38 (1.14 - 1.65)**<br>**9/9 with MRD $\leq$ 2**	
Oral			
po (tab), sd	250	1.48	Vree 1992 [14]
po (tab), sd	500	1.58	Emanuelsson 1987 [15]
po (tab), sd	500	1.24	Selen 1982 [18]
po (tab), sd	500	1.20	Vree 1992 [14]
po (tab), md[a]	500/1000	1.23	Landersdorfer 2010 [22]
po (tab), sd	1000	1.61	Emanuelsson 1987 [15]
po (tab), sd	1000	1.88	Selen 1982 [18]
po (tab), sd	1000	1.23	Shen 2019 [23]
po (tab), sd	1000	1.25	Vree 1992 [14]
po (tab), sd	1000	1.40	Vree 1993 [20]
po (-), bid	1000	1.64	Smith 1980 [24]
po (tab), bid	1000	1.17	Wiebe 2020 [11]
po (tab), md[b]	1000/250/500	1.08	Landersdorfer 2009 [25]
po (tab), sd	1500	1.33	Vree 1992 [14]
po (tab), sd	2000	1.67	Dayton 1963 – Subject A [21]
po (tab), sd	2000	2.90	Dayton 1963 – Subject B [21]
po (tab), sd	2000	1.67	Emanuelsson 1987 [15]
po (tab), sd	2000	1.43	Selen 1982 [18]
**MRD**		**1.50 (1.08 - 2.90)**<br>**17/18 with MRD $\leq$ 2**	
**Overall MRD**		**1.46 (1.08 - 2.90)**<br>**26/27 with MRD $\leq$ 2**	

bid: twice daily, **iv:** intravenous, **md:** multiple dose, **MRD:** mean relative deviation, **po:** oral, **route:** route of administration, **sd:** single dose, **tab:** tablet.

[a] probenecid administration: 500 mg (0 h), 1000 mg (8 h) and 500 mg (14.5, 20.5, 26.5 h)

[b] probenecid administration: 1000 mg (0, 8 h), 250 mg (16, 24 h) and 500 mg (34, 46, 58, 70 h)

2.5.3 AUC_{last} and C_{max} goodness-of-fit plots

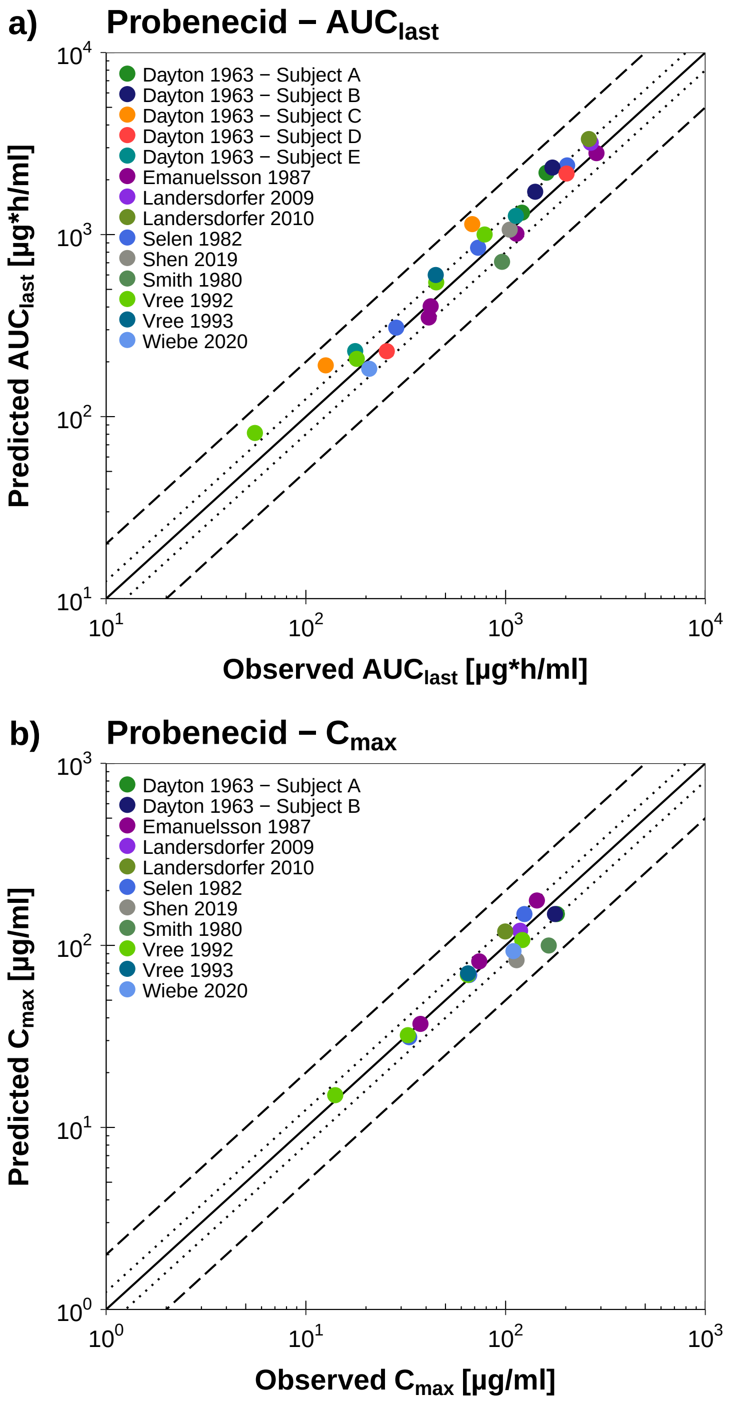

Figure S2.5.2: Probenecid AUC_{last} and C_{max} values. Predicted compared to observed probenecid (a) AUC_{last} and (b) C_{max} values of all analyzed clinical studies. The solid line marks the line of identity. The dotted lines indicate 1.25-fold, the dashed lines indicate 2-fold deviation. Details on dosing regimens, study populations and literature references are summarized in Table S2.2.1. The individual AUC_{last} and C_{max} values, mean GMFE values and ranges are listed in Table S2.5.2.

2.5.4 Predicted and observed AUC_{last} and C_{max} values with mean GMFE values and ranges

Table S2.5.2: Predicted and observed probenecid AUC_{last} and C_{max} values

		AUC_{last}			C_{max}				
**Route**	**Dose [mg]**	**Pred [µg*h/ml]**	**Obs [µg*h/ml]**	**Pred/Obs**	**Pred [µg/ml]**	**Obs [µg/ml]**	**Pred/Obs**	**t_{last} [h]**	**Reference**
Intravenous									
iv (-), sd	464.2	191.28	125.74	1.52	-	-	-	5.5	Dayton 1963 - Subject C [21]
iv (-), sd	464.2	228.79	254.14	0.90	-	-	-	7.5	Dayton 1963 - Subject D [21]
iv (-), sd	464.2	229.15	176.58	1.30	-	-	-	7.5	Dayton 1963 - Subject E [21]
iv (15 min), sd	500	403.83	421.57	0.96	-	-	-	30	Emanuelsson 1987 [15]
iv (-), sd	1860	1319.73	1206.96	1.09	-	-	-	9.0	Dayton 1963 - Subject A [21]
iv (-), sd	1860	1719.79	1406.39	1.22	-	-	-	36.0	Dayton 1963 - Subject B [21]
iv (-), sd	1860	1141.85	680.72	1.68	-	-	-	7.5	Dayton 1963 -Subject C [21]
iv (-), sd	1860	2164.61	2019.01	1.07	-	-	-	22.5	Dayton 1963 - Subject D [21]
iv (-), sd	1860	1266.88	1125.37	1.13	-	-	-	8.5	Dayton 1963 - Subject E [21]
**GMFE**				**1.24 (1.04 - 1.68)**<br>**9/9 with GMFE $\leq$ 2**					
Oral									
po (tab), sd	250	81.36	55.57	1.46	15.04	14.03	1.07	14.0	Vree 1992 [14]
po (tab), sd	500	350.59	412.16	0.85	37.05	37.47	0.99	30.0	Emanuelsson 1987 [15]
po (tab), sd	500	308.18	283.47	1.09	31.31	32.90	0.95	24.0	Selen 1982 [18]
po (tab), sd	500	207.69	179.82	1.16	32.13	32.41	0.99	14.5	Vree 1992 [14]
po (tab), md[a]	500/1000	3350.31	2612.01	1.28	119.28	99.55	1.20	58.5	Landersdorfer 2010 [22]
po (tab), sd	1000	1011.30	1132.59	0.89	81.71	73.80	1.11	48.0	Emanuelsson 1987 [15]
po (tab), sd	1000	846.82	728.26	1.16	68.77	65.70	1.05	30.0	Selen 1982 [18]
po (tab), sd	1000	1064.28	1048.36	1.02	82.93	113.42	0.73	25.0	Shen 2019 [23]
po (tab), sd	1000	546.41	449.39	1.22	68.81	64.72	1.06	15.0	Vree 1992 [14]
po (tab), sd	1000	600.06	446.91	1.34	70.21	64.86	1.08	15.0	Vree 1993 [20]

AUC_{last}: area under the plasma concentration-time curve (AUC) from the time of drug administration to the time of the last concentration measurement, **bid:** twice daily, **C_{max}:** peak plasma concentration, **GMFE:** geometric mean fold error, **iv:** intravenous, **md:** multiple dose, **obs:** observed, **po:** oral, **pred:** predicted, **route:** route of administration, **sd:** single dose, **t_{last}:** time of the last concentration measurement. GMFE values are means and ranges.

[a] probenecid administration: 500 mg (0 h), 1000 mg (8 h) and 500 mg (14.5, 20.5, 26.5 h)

[b] probenecid administration: 1000 mg (0, 8 h), 250 mg (16, 24 h) and 500 mg (34, 46, 58, 70 h)

Table S2.5.2: Predicted and observed probenecid AUC_{last} and C_{max} values *(continued)*

		AUC_{last}			C_{max}				
**Route**	**Dose [mg]**	**Pred [µg*h/ml]**	**Obs [µg*h/ml]**	**Pred/Obs**	**Pred [µg/ml]**	**Obs [µg/ml]**	**Pred/Obs**	**t_{last} [h]**	**Reference**
po (-), bid	1000	708.92	959.74	0.74	99.88	164.40	0.61	21.0	Smith 1980 [24]
po (tab), bid	1000	183.17	207.54	0.88	93.05	109.50	0.85	4.0	Wiebe 2020 [11]
po (tab), md[b]	1000/250/500	3194.25	2668.85	1.20	120.42	118.17	1.02	58.0	Landersdorfer 2009 [25]
po (tab), sd	1500	1001.35	785.08	1.28	106.98	121.22	0.88	17.0	Vree 1992 [14]
po (tab), sd	2000	2187.34	1602.71	1.36	148.68	180.50	0.82	25.0	Dayton 1963 - Subject A [21]
po (tab), sd	2000	2335.17	1715.52	1.36	148.68	176.69	0.84	33.0	Dayton 1963 - Subject B [21]
po (tab), sd	2000	2801.92	2850.62	0.98	176.51	143.40	1.23	48.0	Emanuelsson 1987 [15]
po (tab), sd	2000	2399.30	2032.30	1.18	148.67	124.20	1.20	48.0	Selen 1982 [18]
**GMFE**				**1.22 (1.02 - 1.46)**			**1.16 (1.01 - 1.64)**		
				**18/18 with GMFE $\leq$ 2**			**18/18 with GMFE $\leq$ 2**		
**Overall GMFE**				**1.23 (1.02 - 1.68)**			**1.16 (1.01 - 1.64)**		
				**27/27 with GMFE $\leq$ 2**			**18/18 with GMFE $\leq$ 2**		

AUC_{last}: area under the plasma concentration-time curve (AUC) from the time of drug administration to the time of the last concentration measurement, **bid:** twice daily, **C_{max}:** peak plasma concentration, **GMFE:** geometric mean fold error, **iv:** intravenous, **md:** multiple dose, **obs:** observed, **po:** oral, **pred:** predicted, **route:** route of administration, **sd:** single dose, **t_{last}:** time of the last concentration measurement. GMFE values are means and ranges.

[a] probenecid administration: 500 mg (0 h), 1000 mg (8 h) and 500 mg (14.5, 20.5, 26.5 h)

[b] probenecid administration: 1000 mg (0, 8 h), 250 mg (16, 24 h) and 500 mg (34, 46, 58, 70 h)

2.5.5 Sensitivity analysis

Sensitivity of the final probenecid PBPK model to single parameters (local sensitivity analysis) was analyzed, measured as relative change of the $AUC_{0\text{-}12}$ of the last administration interval for simulation of the highest recommended probenecid dose (250 mg twice daily for 7 days, followed by 500 mg twice daily for 14 days).

The sensitivity analysis results are illustrated in Figure S2.5.3. Parameters were included into the analysis if they were optimized (OAT3 catalytic rate constant, OAT3 Michaelis-Menten constant, UGT1A9 catalytic rate constant, lipophilicity, GFR fraction, dissolution function parameters, transcellular intestinal permeability), if they are associated with optimized parameters (UGT1A9 Michaelis-Menten constant) or if they might have a strong impact due to calculation methods used in the model (solubility, fraction unbound in plasma, blood/plasma concentration ratio). Applying a threshold of 0.5, the probenecid model is sensitive to the values of the UGT1A9 catalytic rate constant (optimized) and Michaelis-Menten constant (literature value), the probenecid fraction unbound in plasma (literature value), the OAT3 catalytic rate constant (optimized) and the probenecid lipophilicity (optimized).

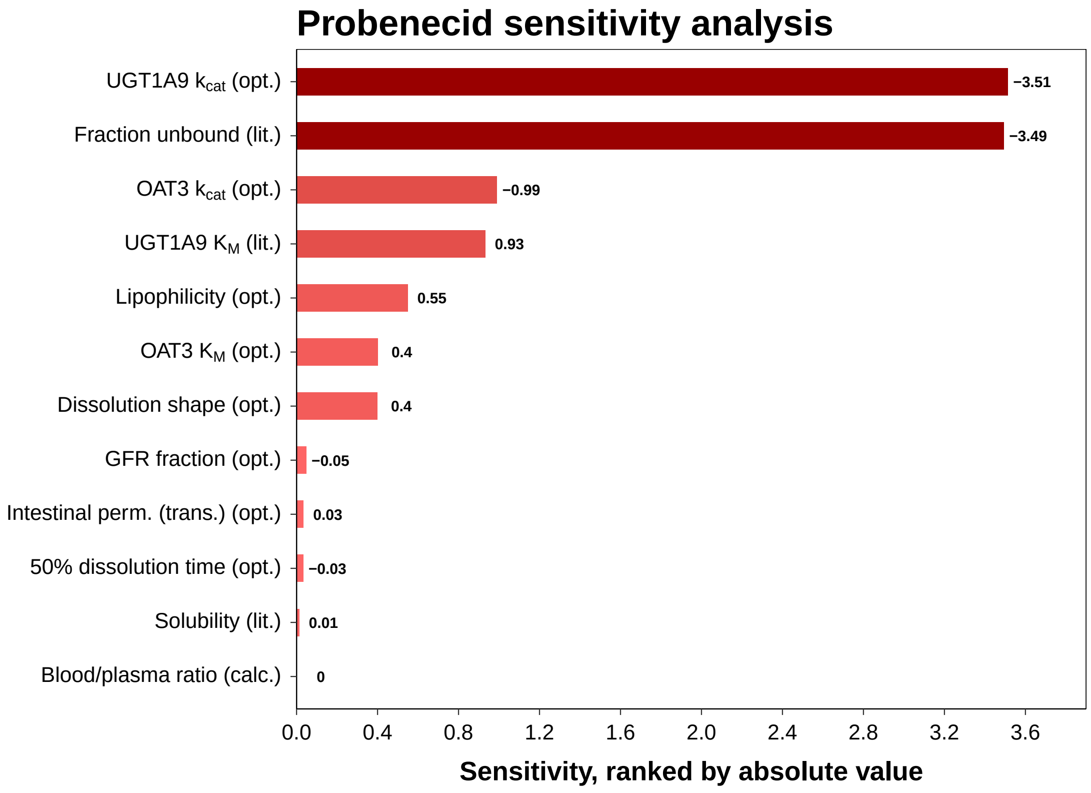

Figure S2.5.3: Probenecid PBPK model sensitivity analysis. Sensitivity of the model to single parameters, measured as change of the simulated $AUC_{0\text{-}12}$ under steady-state conditions of a 500 mg twice daily probenecid regimen. A sensitivity value of +0.5 signifies that a 100% increase of the examined parameter causes a 50% increase of the simulated $AUC_{0\text{-}12}$. **Calc.:** calculated, **GFR:** glomerular filtration rate, **intestinal perm. (trans.):** transcellular intestinal permeability, $\mathbf{k_{cat}}$**:** catalytic rate constant, $\mathbf{K_M}$**:** Michaelis-Menten constant, **lit.:** literature, **OAT3:** organic anion transporter 3, **opt.:** optimized, **UGT1A9:** uridine 5'-diphospho-glucuronosyltransferase 1A9.

3 PBPK modeling of furosemide

3.1 PBPK model development

Furosemide is a loop diuretic agent to treat edema or high blood pressure [31]. The recommended dose is 40 mg once daily [32]. Furosemide is highly bound to plasma proteins (f_u = 2.2%) [33]. The volume of distribution after intravenous administration is 8 to 12 l [34, 35]. The main metabolic pathway is glucuronidation by UGT1A9 and UGT1A1 [36] and the bioavailability of orally administered furosemide shows a high interindividual variability (37-83%) [31]. 50% to 80% of an intravenously administered and 20% to 55% of an orally administered furosemide dose are excreted unchanged in the urine [31]. The FDA lists furosemide as a clinical OAT1/OAT3 substrate [19].

The furosemide PBPK model was developed using 42 different clinical studies including intravenous (single dose) and oral (single- and multiple dose) administration. In addition, 27 studies reported fraction excreted unchanged in urine profiles following intravenous and oral administration. Please refer to Table S3.2.1 for the complete list of clinical studies used in the presented analysis.

The final furosemide PBPK model applies uptake into kidney cells via OAT3, glucuronidation by UGT1A9, secretion into urine via MRP4 and glomerular filtration. The drug-dependent parameters of the final furosemide PBPK model are summarized in Table S3.3.1.

Population predicted compared to observed plasma concentration-time profiles of all 42 clinical studies included in this analysis are shown in semilogarithmic (Figure S3.4.1) and linear plots (Figure S3.4.2). Individual predicted compared to observed plasma concentration-time profiles of all 42 clinical studies included in this analysis are shown in semilogarithmic (Figure S3.4.3) and linear plots (Figure S3.4.4). Population predicted compared to observed fraction excreted unchanged in urine profiles are shown in Figure S3.4.5. Individual predicted compared to observed fraction excreted unchanged in urine profiles are shown in Figure S3.4.6. Figure S3.5.1 shows predicted compared to observed plasma concentration values in a goodness-of-fit plot. Table S3.5.1 lists the MRD values of all 42 studies. The correlation of predicted compared to observed furosemide AUC_{last} and C_{max} values is presented in Figure S3.5.2, further demonstrating the good model performance with 41/42 predicted AUC_{last} and 24/25 predicted C_{max} values within 2-fold of the observed data. The individual values and mean GMFE values and ranges are listed in Table S3.5.2.

The sensitivity analysis results of a simulation of 80 mg furosemide once daily are illustrated in Figure S3.5.3. Applying a threshold of 0.5, the furosemide model is sensitive to the values of the fraction unbound in plasma (literature value) and the OAT3 catalytic rate constant (optimized).

3.2 Clinical studies

Table S3.2.1: Furosemide study table

Dose [mg]	Route	n	Age [years]	Weight [kg]	Height [cm]	Females [%]	Dataset	Reference
20	iv (bolus), sd	11	26 ± 4	-	-	-	training	Haegeli 2007 [37]
20	iv (5 min), sd	9	28-47	53-70	-	-	test	Rosenkranz 1992 [38]
22	iv (bolus), sd	11	21-35	73.5	-	0	test	Tilstone 1978 [39]
35.5	iv (bolus), sd	6	18-23	58-84	-	34	test	Alván 1988 [40]
40	iv (bolus), sd	8	58 (27-74)	71 (62-74)	-	25	test	Andreasen 1977 [41]
40	iv (bolus), sd	7	19-46	58-82	-	14	test	González 1982 [34]
40	iv (bolus), sd	8	22-32	55-82	-	63	test	Hammarlund 1984 [42]
40	iv (bolus), sd	6	20-25	-	-	0	test	Homeida 1977 [43]
40	iv (bolus), sd	7	30 (20-45)	-	-	57	training	Keller 1981 [44]
40	iv (bolus), sd	5	18-26	62-76	-	0	test	Lambert 1983 [35]
40	iv (bolus), sd	5	-	-	-	-	training	Rupp 1974 [45]
40	iv (2 min), sd	18	24 (20-31)	71 (61-83)	-	0	test	Waller 1982 [46]
40	iv (3 min), sd	4	21-33	65-77	-	0	test	Smith 1980 [24]
40	iv (3 min), sd	9	21-40	84 (70-130)	-	0	test	Smith 1980 [47]
80	iv (bolus), sd	6	20-25	-	-	0	training	Branch 1977 [48]
80	iv (bolus), sd	4	18-45	-	-	-	test	Kelly 1974 [49]
80	iv (bolus), sd	6	19-39	72 ± 4	-	0	test	Rane 1978 [50]
80	iv (bolus), sd	10	23-42	74	-	0	training	Verbeeck 1982 [51]
80	iv (2 min), sd	10	20-34	-	-	0	test	Andreasen 1981 [52]
80	iv (2 min), sd	10	27 (22-35)	71 (57-83)	-	0	test	Andreasen 1983 [53]
1	po (sol), sd	28	36 (20-55)	84 (63-105)	179 (163-195)	0	training	Stopfer 2018 [54]
5	po (sol), sd	22	37 (23-49)	84 (68-100)	180 (171-188)	0	training	Stopfer 2016 [55]
20	po (sol), sd	21	24 (21-34)	74 (65-89)	-	0	test	Waller 1985 [56]
20	po (tab), sd	11	26 ± 4	-	-	-	test	Haegeli 2007 [37]
20	po (tab), qd	21	-	-	-	19	test	FDA 2006 [57]

iv: intravenous, **n:** number of individuals studied, **po:** oral, **qd:** once daily, **route:** route of administration, **sd:** single dose, **sol:** solution, **tab:** tablet, **test:** test dataset (model evaluation), **training:** training dataset (parameter optimization). Values are means ± standard deviation or ranges.

Table S3.2.1: Furosemide study table *(continued)*

Dose [mg]	Route	n	Age [years]	Weight [kg]	Height [cm]	Females [%]	Dataset	Reference
20	po (-), qd	22	31 (19-42)	70 ± 8	169 ± 8	18	test	Vaidyanathan 2008 [58]
40	po (sol), sd	18	24 (20-31)	71 (61-83)	-	0	training	Waller 1982 [46]
40	po (sol), sd	21	24 (21-34)	74 (65-89)	-	0	training	Waller 1985 [56]
40	po (sol), sd	21	27 (19-35)	74 (67-85)	-	0	test	Waller 1988 [59]
40	po (sol), sd	15	34 (21-52)	79 (62-95)	179 (169-191)	0	training	Wiebe 2020 [11]
40	po (tab), sd	10	63	80	170	20	test	Ballester 2015 [60]
40	po (tab), sd	12	38 (23-52)	74 (59-97)	177 (166-190)	0	training	Bindschedler 1997 [61]
40	po (tab), sd	8	22-32	55-82	-	63	test	Hammarlund 1984 [42]
40	po (tab), sd	12	18-42	-	-	-	training	Martin 1984 [62]
40	po (tab), sd	12	21-35	-	-	0	test	Rakhit 1987 [63]
40	po (tab), sd	6	-	-	-	-	test	Rupp 1974 [45]
40	po (tab), sd	18	24 (20-31)	71 (61-83)	-	0	test	Waller 1982 [46]
40	po (tab), qd	15	20-43	-	-	-	training	FDA 2005 [64]
44	po (sol), sd	5	21-35	73.5	-	0	test	Tilstone 1978 [39]
80	po (sol), sd	8	18-45	-	-	-	test	Kelly 1974 [49]
80	po (sol), sd	21	24 (21-34)	74 (65-89)	-	0	test	Waller 1985 [56]
80	po (tab), sd	8	18-45	-	-	-	test	Kelly 1974 [49]
80	po (tab), sd	12	24 (18-29)	82 (69-101)	-	0	training	Shoaf 2007 [65]
80	po (tab), sd	9	35 ± 6	78 ± 8	-	33	test	Vree 1995 [33]

iv: intravenous, **n:** number of individuals studied, **po:** oral, **qd:** once daily, **route:** route of administration, **sd:** single dose, **sol:** solution, **tab:** tablet, **test:** test dataset (model evaluation), **training:** training dataset (parameter optimization). Values are means $\pm$ standard deviation or ranges.

3.3 Drug-dependent parameters

Table S3.3.1: Drug-dependent parameters of the furosemide PBPK model

Parameter	Value	Unit	Source	Literature	Reference	Description
MW	330.74	g/mol	Literature	330.74	[26]	Molecular weight
pKa (acid)	3.51	-	Literature	3.51, 3.60	[67, 68]	Acid dissociation constant
pKa (base)	9.87	-		9.87, 10.15		
Solubility (FaSSIF)	3.20	mg/ml	Literature	0.03 (pH 1.20), 0.43 (pH 5.00), 0.68 (FeSSIF), 3.02 (pH 6.50), 3.20 (FaSSIF)	[69]	Solubility
logP	-0.24	-	Literature	-1.20, -0.83, -0.24, 2.56	[28, 70, 71]	Lipophilicity
fu	2.20	%	Literature	1.20, 1.40, 1.50, 1.71, 2.20, 2.30, 3.70, 4.10, 4.60, 5.00	[23, 33, 41, 47, 50, 53, 72–76]	Fraction unbound in plasma
OAT3 K_M	21.50	µmol/l	Literature	21.50	[77]	OAT3 Michaelis-Menten constant
OAT3 k_{cat}	8226.82	1/min	Optimized	-	-	OAT3 transport rate constant
UGT1A9 K_M	72.00	µmol/l	Measured[a]	-	-	UGT1A9 Michaelis-Menten constant
UGT1A9 k_{cat}	954.33	1/min	Optimized	-	-	UGT1A9 catalytic rate constant
MRP4 K_M	27.96	µmol/l	Measured[b]	-	-	MRP4 Michaelis-Menten constant
MRP4 k_{cat}	6841.01	1/min	Optimized	-	-	MRP4 transport rate constant
GFR fraction	1.00	-	Assumed	-	-	Fraction of filtered drug in the urine
EHC continuous fraction	1.00	-	Assumed	-	-	Fraction of bile continually released
Partition coefficients	Diverse	-	Calculated	Schmitt	[78]	Cell to plasma partition coefficients
Cellular permeability	1.91E-5	cm/min	Calculated	CdS	[4]	Permeability into the cellular space
Intestinal permeability (trans.)	5.06E-7	cm/min	Optimized	5.75E-8	Calculated	Transcellular intestinal permeability
Intestinal permeability (para.)	2.32E-6	cm/min	Optimized	0	Calculated	Paracellular intestinal permeability
Tablet fasted Weibull shape	0.53	-	Calculated	Langenbucher	[79, 80]	Dissolution profile shape
Tablet fasted Weibull time	26.77	min	Optimized	-	[61, 62, 64, 65]	Dissolution time (50% dissolved)

CdS: charge-dependent Schmitt calculation method, **EHC:** enterohepatic circulation, **FaSSIF:** fasted state simulated intestinal fluid, **FeSSIF:** fed state simulated intestinal fluid, **GFR:** glomerular filtration rate, **Langenbucher:** Langenbucher calculation method, **MRP4:** multidrug resistance-associated protein 4, **OAT3:** organic anion transporter 3, **para.:** paracellular, **Schmitt:** Schmitt calculation method, **trans.:** transcellular, **UGT1A9:** uridine 5'-diphospho-glucuronosyltransferase 1A9.

[a] The furosemide UGT1A9 K_M was determined using Corning® Supersomes™ Human UGT1A9 with different furosemide concentrations (7.2-720 µmol/l)

[b] The furosemide MRP4 K_M was determined using membrane vesicles overexpressing human MRP4 provided by Solvo Biotechnology (Budapest, Hungary) with different furosemide concentrations (0.1-500 µmol/l) [66]

3.4 Profiles

3.4.1 Semilogarithmic plots - Plasma - Population predictions

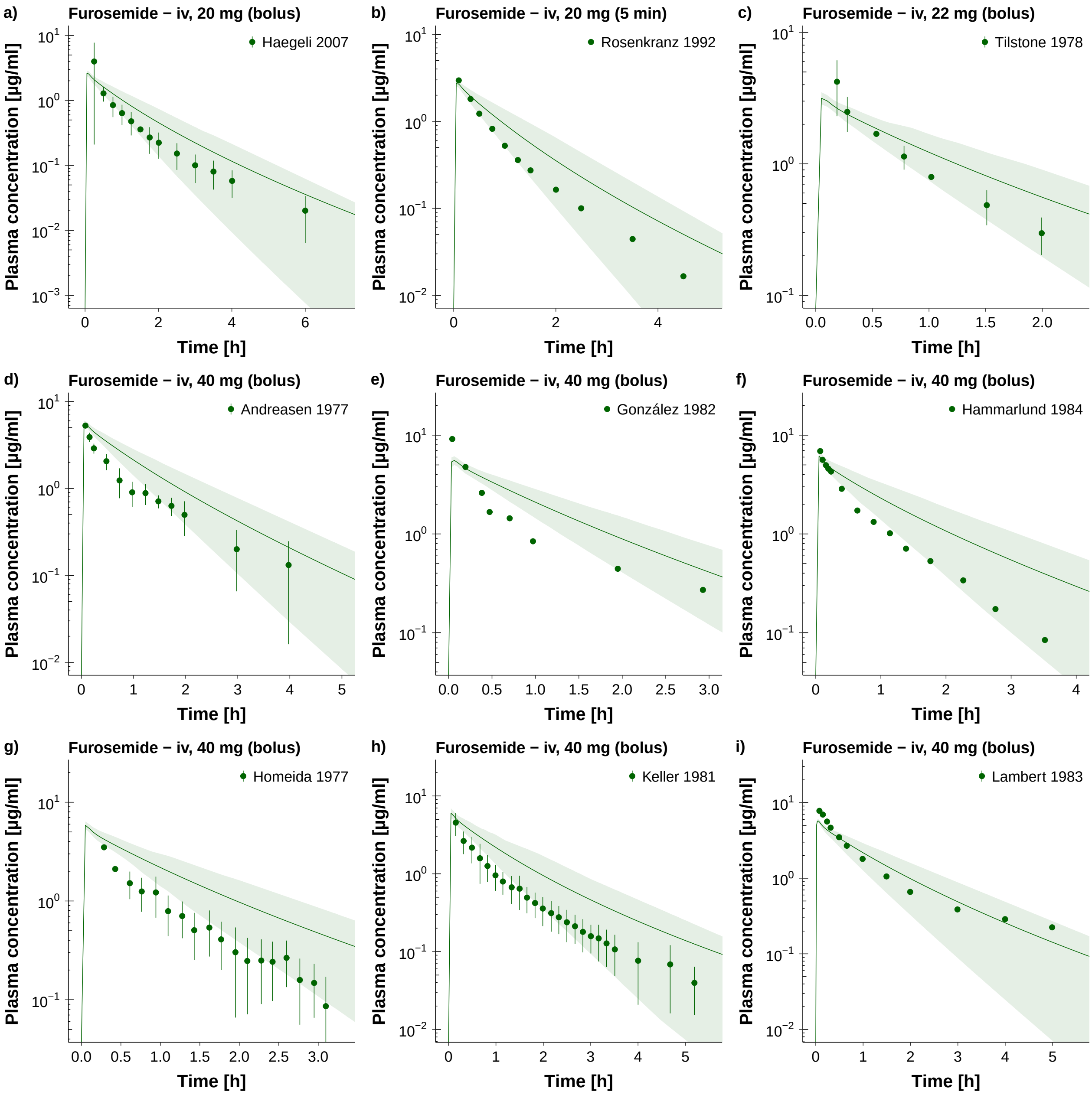

Figure S3.4.1: Furosemide plasma concentration-time profiles. Population predictions of furosemide plasma concentration-time profiles compared to observed data. Observed data are shown as dots ± standard deviation. Population simulation arithmetic means are shown as lines; the shaded areas illustrate the predicted population variation ($Q_{16} - Q_{84}$). Details on dosing regimens, study populations and literature references are summarized in Table S3.2.1.

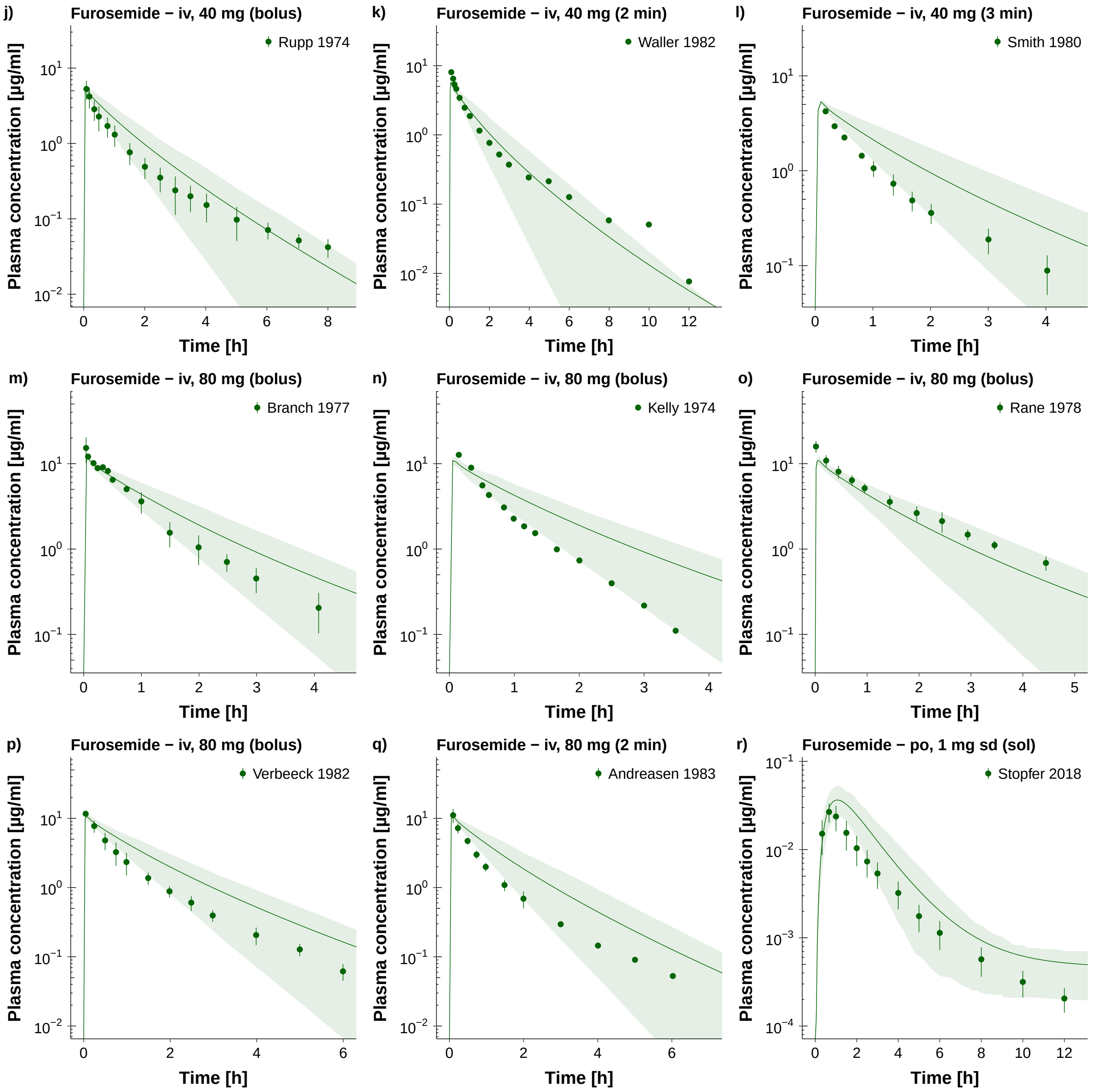

Figure S3.4.1: Furosemide plasma concentration-time profiles. Population predictions of furosemide plasma concentration-time profiles compared to observed data. Observed data are shown as dots $\pm$ standard deviation. Population simulation arithmetic means are shown as lines; the shaded areas illustrate the predicted population variation ($Q_{16} - Q_{84}$). Details on dosing regimens, study populations and literature references are summarized in Table S3.2.1.

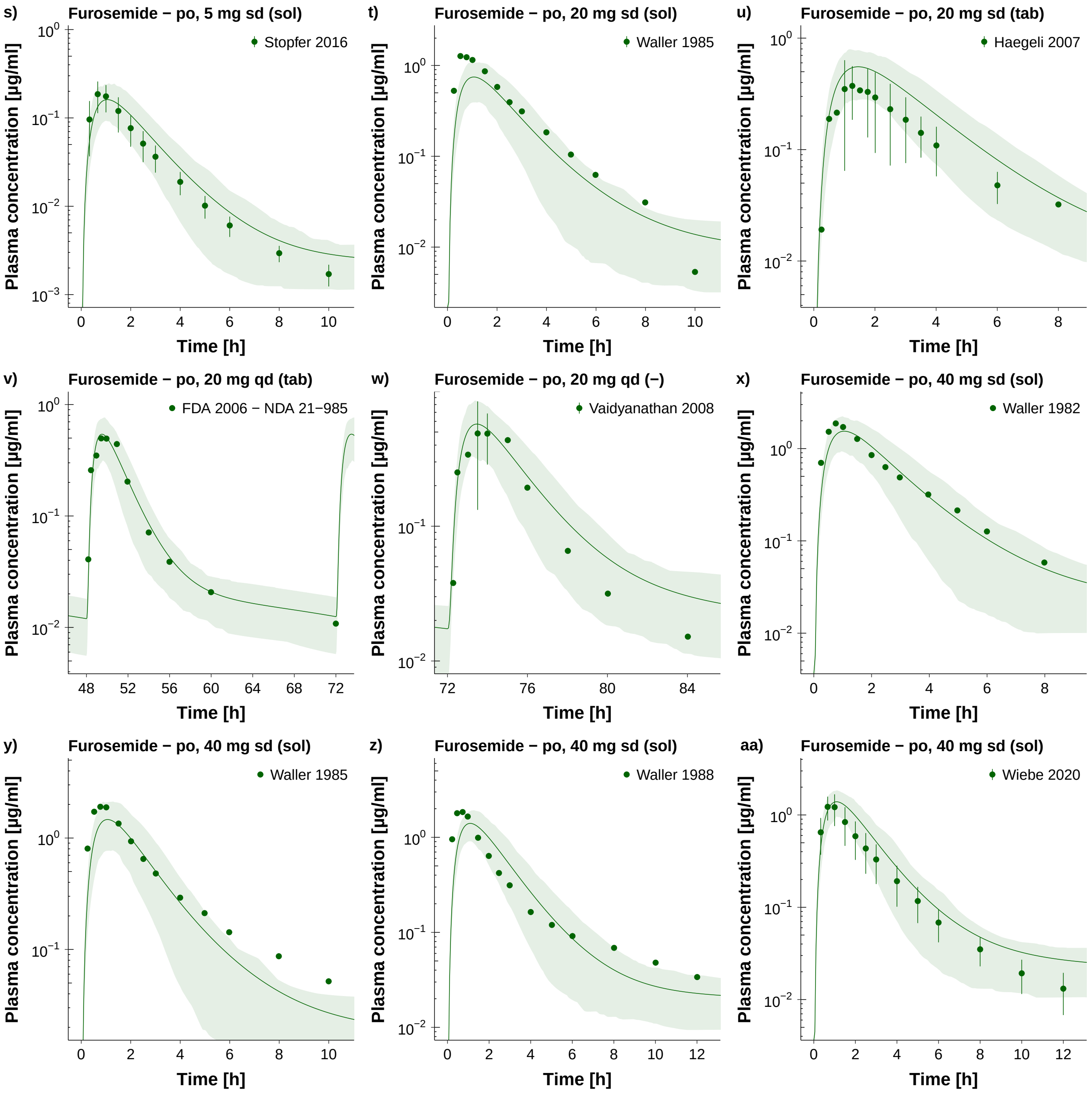

Figure S3.4.1: Furosemide plasma concentration-time profiles. Population predictions of furosemide plasma concentration-time profiles compared to observed data. Observed data are shown as dots $\pm$ standard deviation. Population simulation arithmetic means are shown as lines; the shaded areas illustrate the predicted population variation ($Q_{16} - Q_{84}$). Details on dosing regimens, study populations and literature references are summarized in Table S3.2.1.

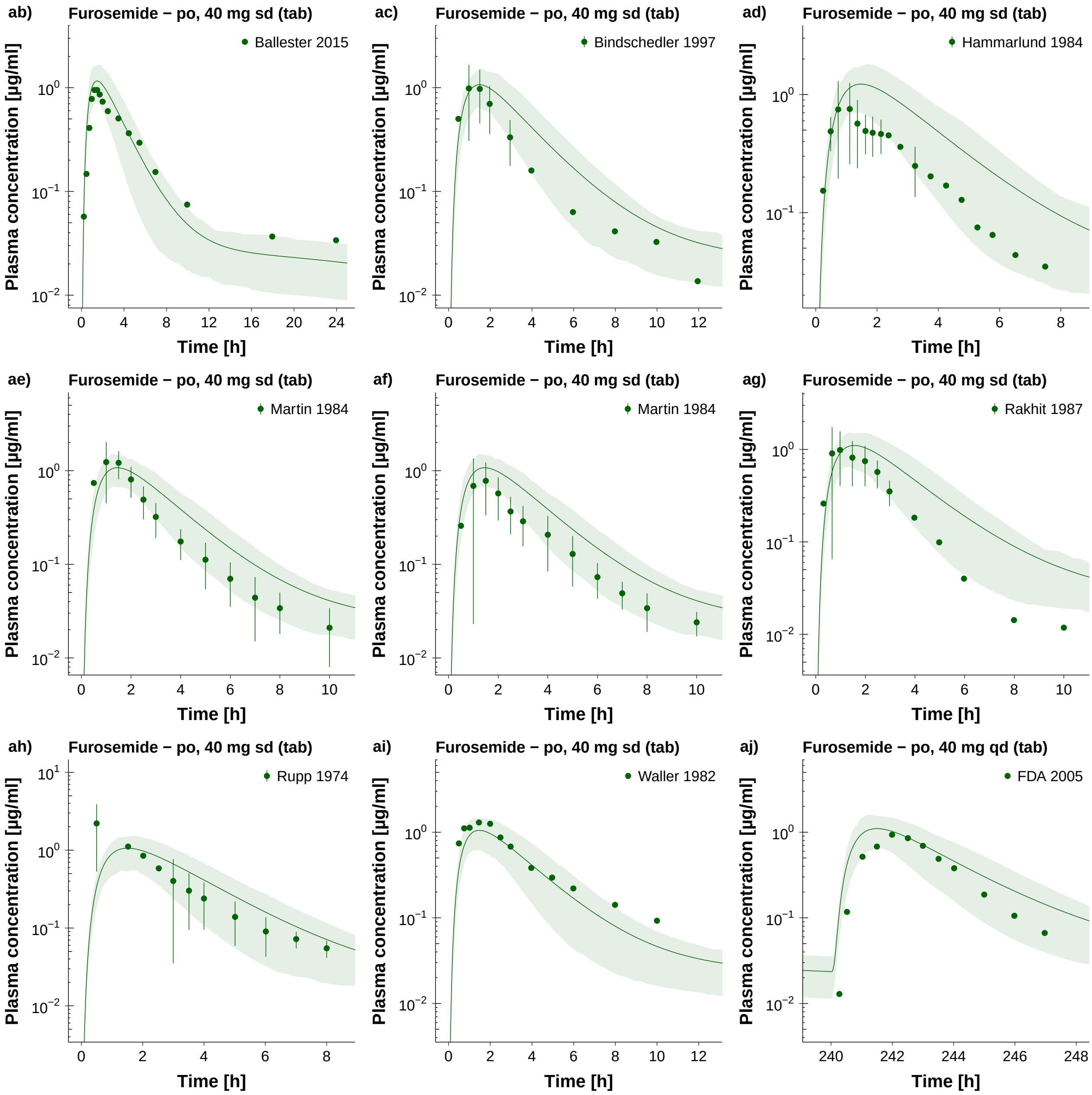

Figure S3.4.1: Furosemide plasma concentration-time profiles. Population predictions of furosemide plasma concentration-time profiles compared to observed data. Observed data are shown as dots $\pm$ standard deviation. Population simulation arithmetic means are shown as lines; the shaded areas illustrate the predicted population variation ($Q_{16} - Q_{84}$). Details on dosing regimens, study populations and literature references are summarized in Table S3.2.1.

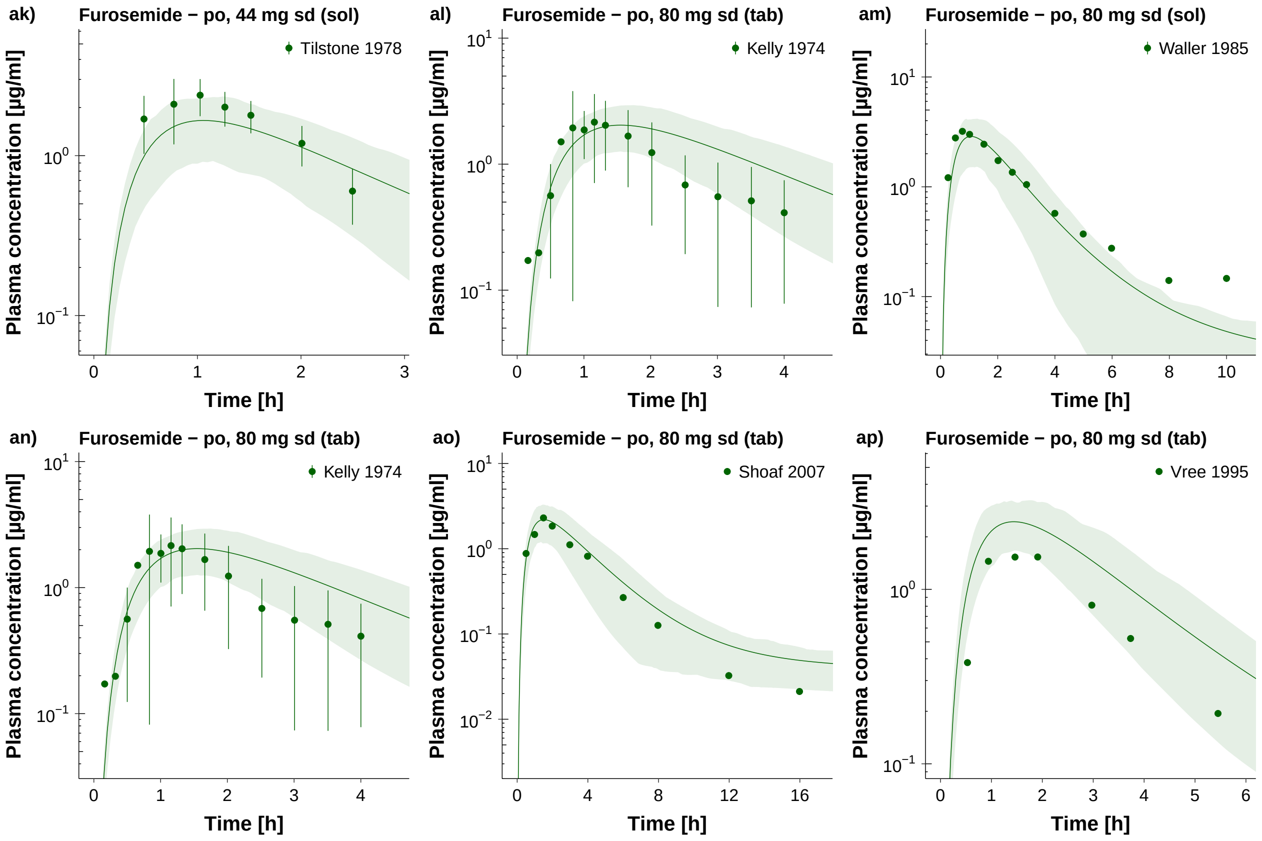

Figure S3.4.1: Furosemide plasma concentration-time profiles. Population predictions of furosemide plasma concentration-time profiles compared to observed data. Observed data are shown as dots $\pm$ standard deviation. Population simulation arithmetic means are shown as lines; the shaded areas illustrate the predicted population variation ($Q_{16} - Q_{84}$). Details on dosing regimens, study populations and literature references are summarized in Table S3.2.1.

3.4.2 Linear plots - Plasma - Population predictions

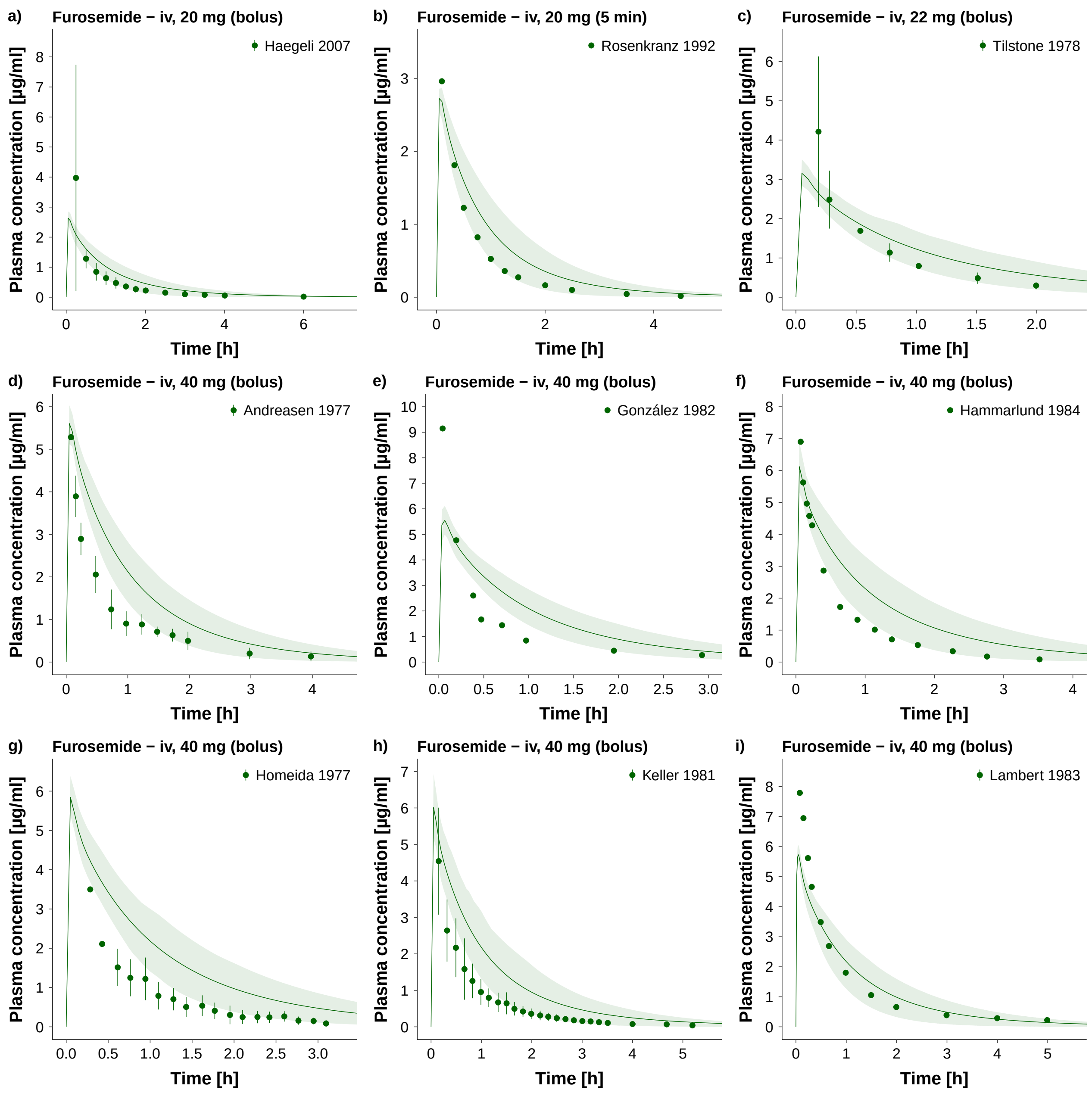

Figure S3.4.2: Furosemide plasma concentration-time profiles. Population predictions of furosemide plasma concentration-time profiles compared to observed data. Observed data are shown as dots $\pm$ standard deviation. Population simulation arithmetic means are shown as lines; the shaded areas illustrate the predicted population variation ($Q_{16} - Q_{84}$). Details on dosing regimens, study populations and literature references are summarized in Table S3.2.1.

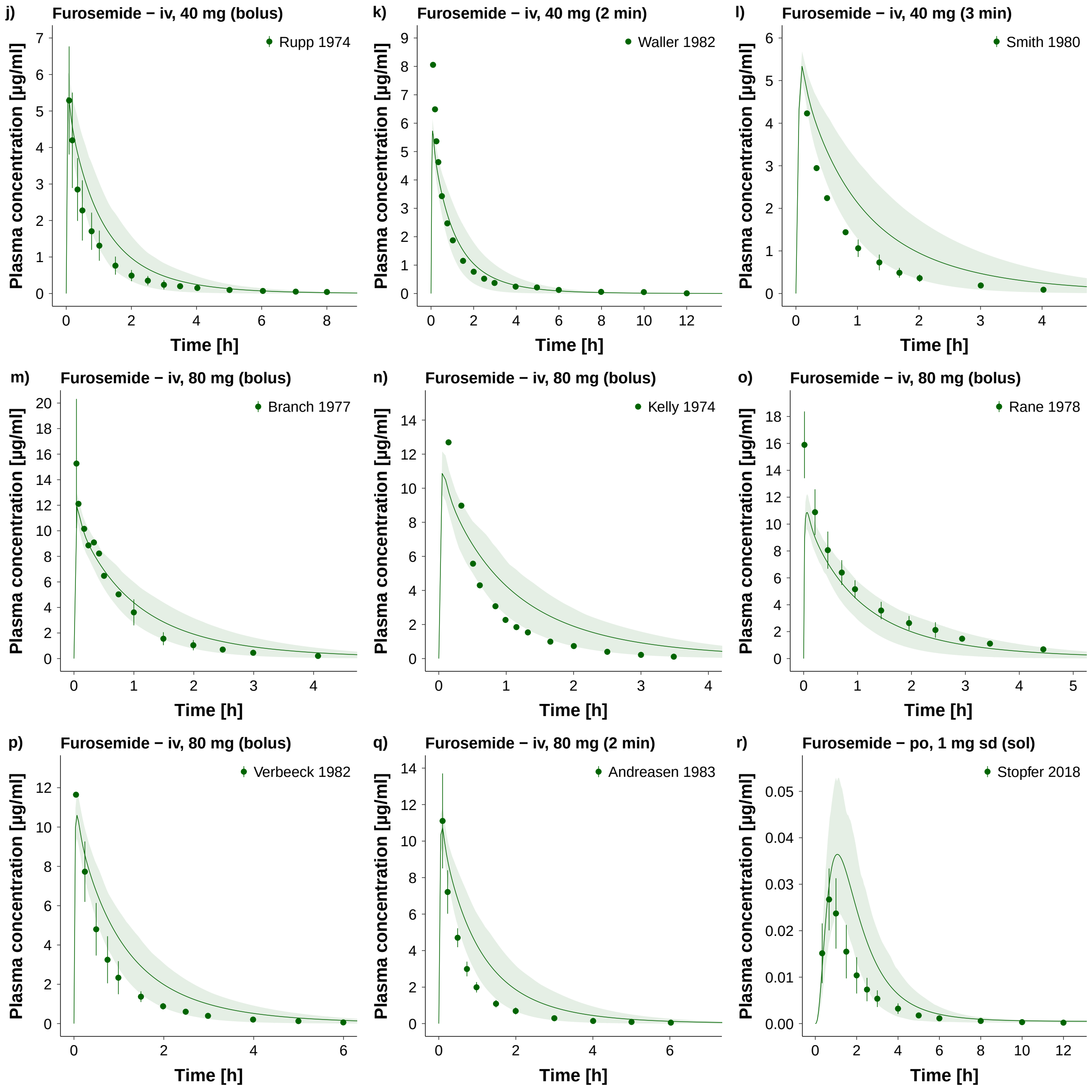

Figure S3.4.2: Furosemide plasma concentration-time profiles. Population predictions of furosemide plasma concentration-time profiles compared to observed data. Observed data are shown as dots ± standard deviation. Population simulation arithmetic means are shown as lines; the shaded areas illustrate the predicted population variation ($Q_{16} - Q_{84}$). Details on dosing regimens, study populations and literature references are summarized in Table S3.2.1.

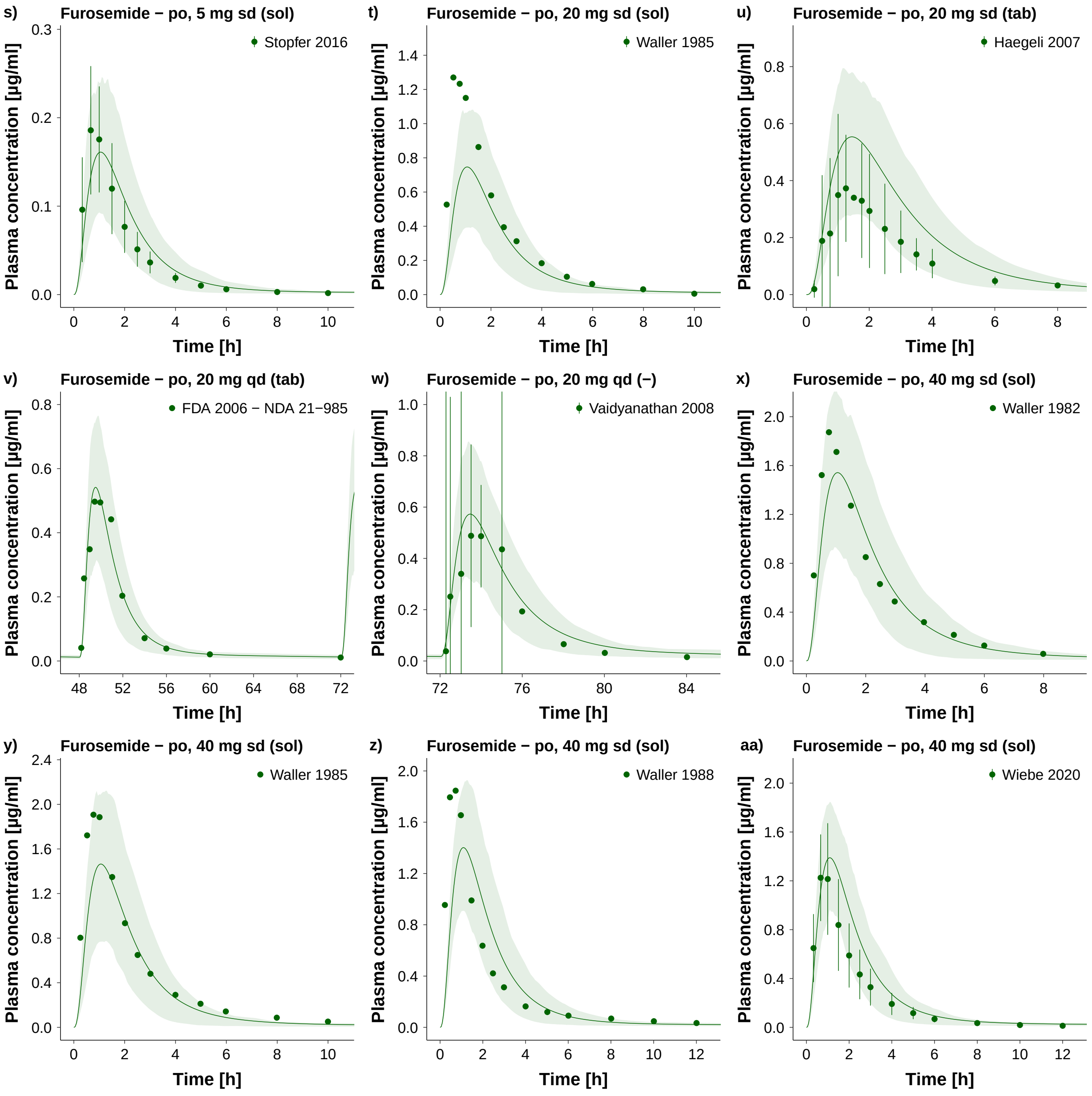

Figure S3.4.2: Furosemide plasma concentration-time profiles. Population predictions of furosemide plasma concentration-time profiles compared to observed data. Observed data are shown as dots ± standard deviation. Population simulation arithmetic means are shown as lines; the shaded areas illustrate the predicted population variation ($Q_{16} - Q_{84}$). Details on dosing regimens, study populations and literature references are summarized in Table S3.2.1.

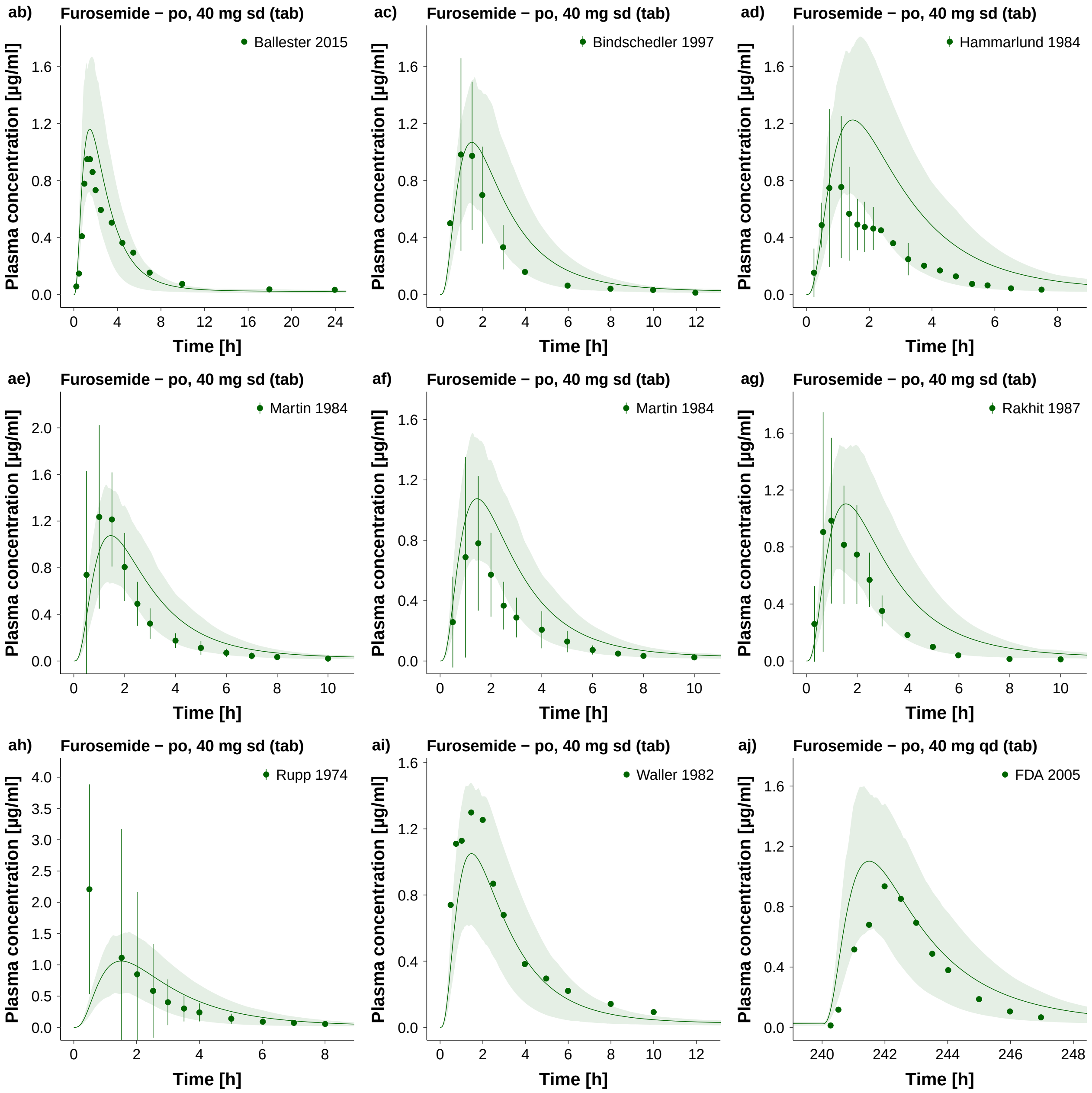

Figure S3.4.2: Furosemide plasma concentration-time profiles. Population predictions of furosemide plasma concentration-time profiles compared to observed data. Observed data are shown as dots ± standard deviation. Population simulation arithmetic means are shown as lines; the shaded areas illustrate the predicted population variation ($Q_{16} - Q_{84}$). Details on dosing regimens, study populations and literature references are summarized in Table S3.2.1.

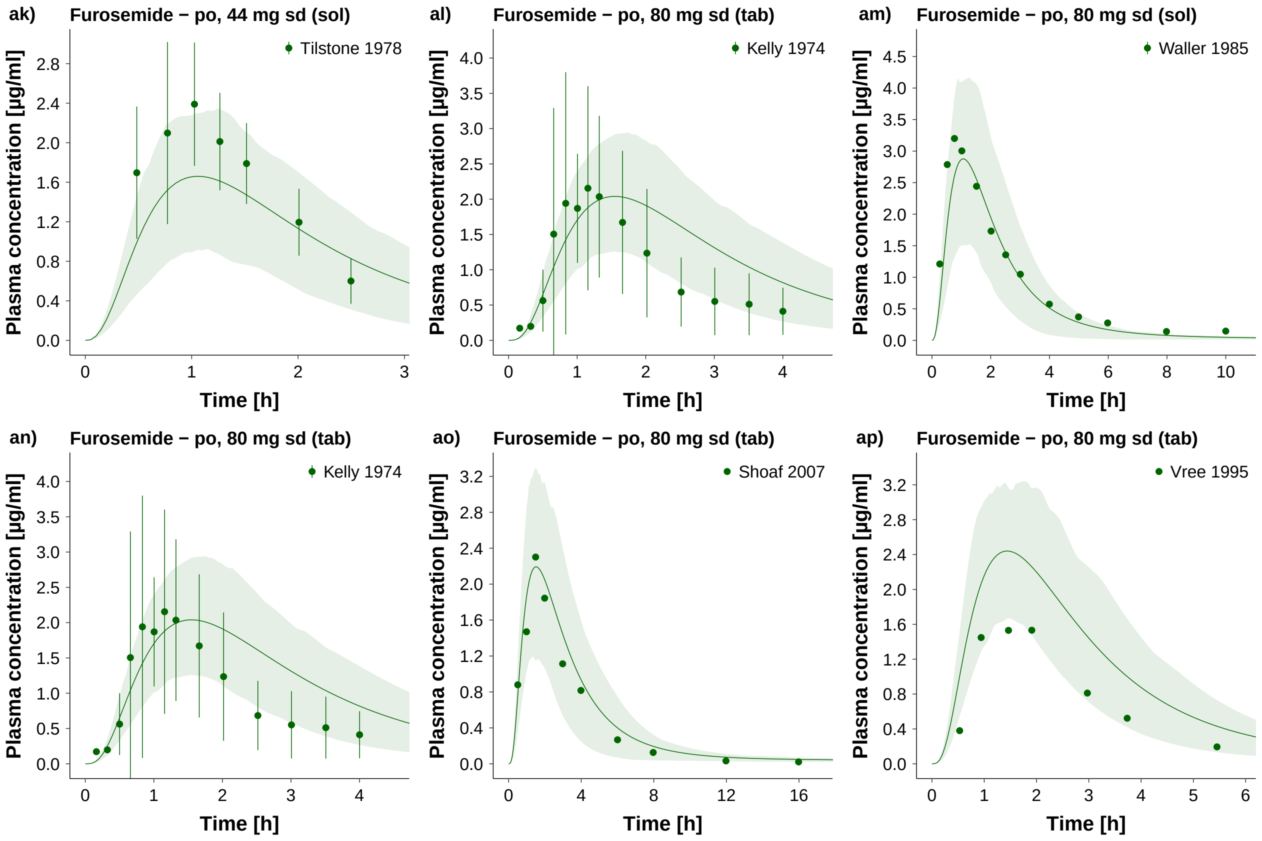

Figure S3.4.2: Furosemide plasma concentration-time profiles. Population predictions of furosemide plasma concentration-time profiles compared to observed data. Observed data are shown as dots $\pm$ standard deviation. Population simulation arithmetic means are shown as lines; the shaded areas illustrate the predicted population variation ($Q_{16} - Q_{84}$). Details on dosing regimens, study populations and literature references are summarized in Table S3.2.1.

3.4.3 Semilogarithmic plots - Plasma - Individual predictions

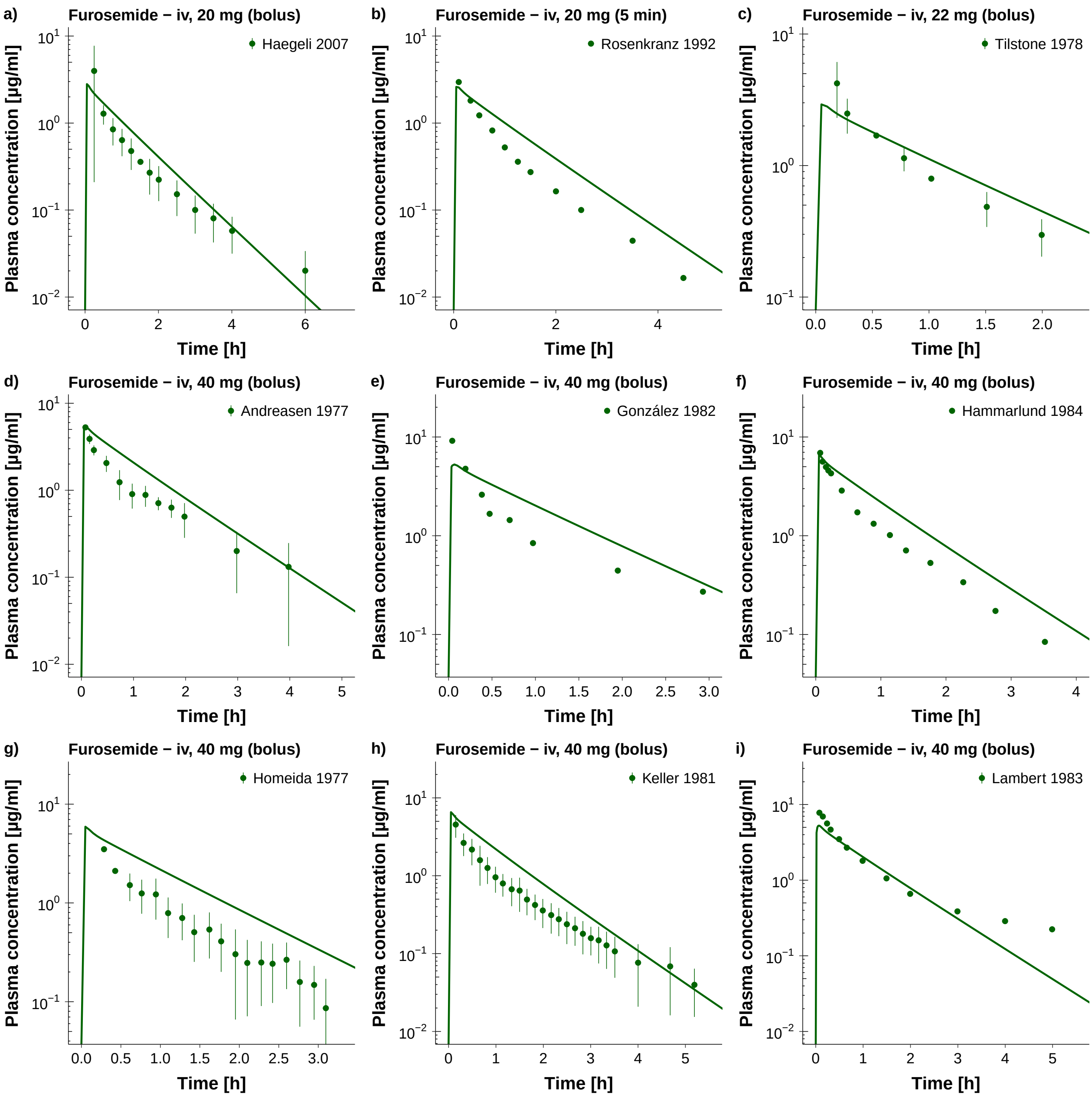

Figure S3.4.3: Furosemide plasma concentration-time profiles. Individual predictions of furosemide plasma concentration-time profiles compared to observed data. Observed data are shown as dots $\pm$ standard deviation. Simulations are shown as lines. Details on dosing regimens, study populations and literature references are summarized in Table S3.2.1.

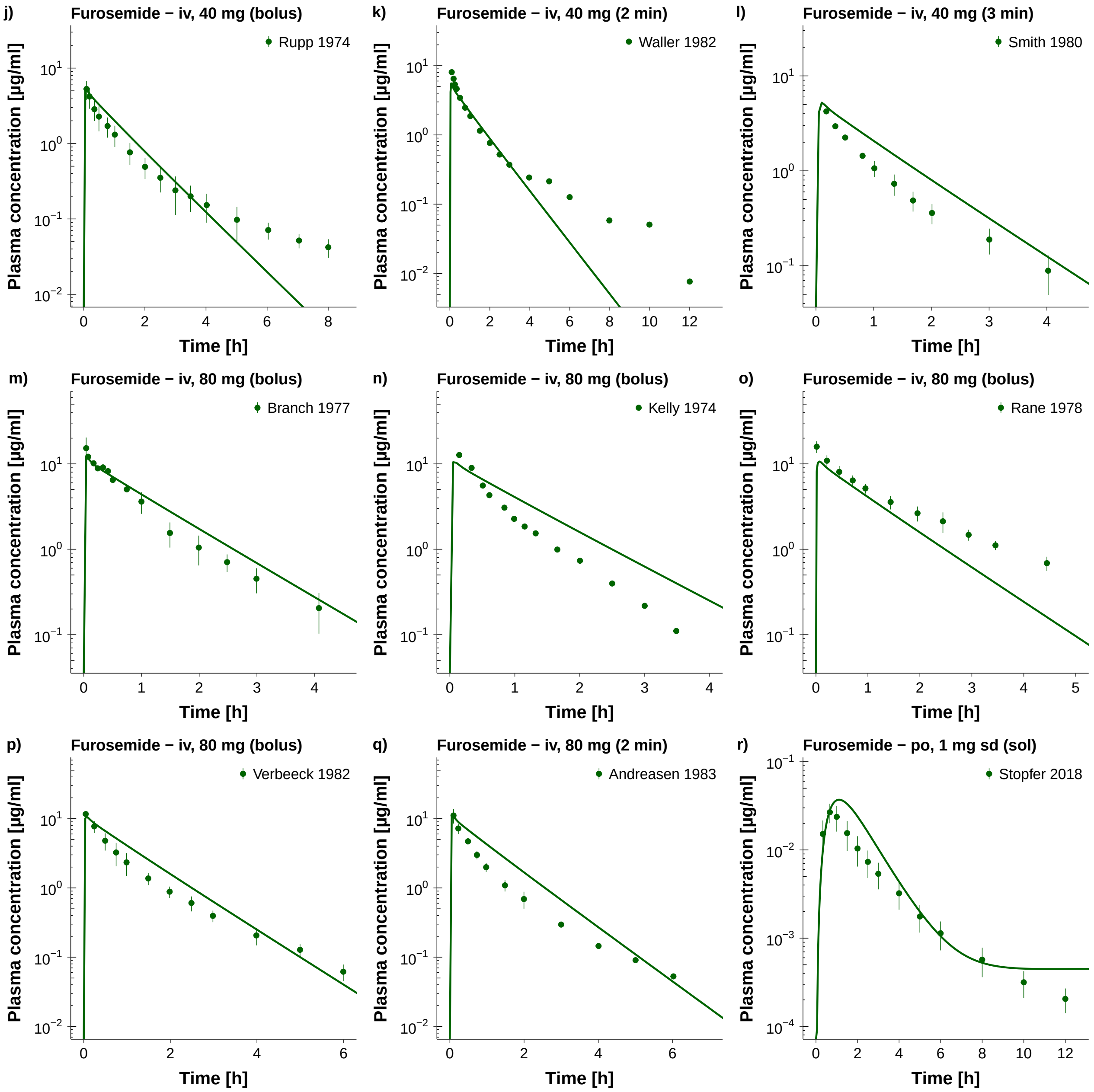

Figure S3.4.3: Furosemide plasma concentration-time profiles. Individual predictions of furosemide plasma concentration-time profiles compared to observed data. Observed data are shown as dots ± standard deviation. Simulations are shown as lines. Details on dosing regimens, study populations and literature references are summarized in Table S3.2.1.

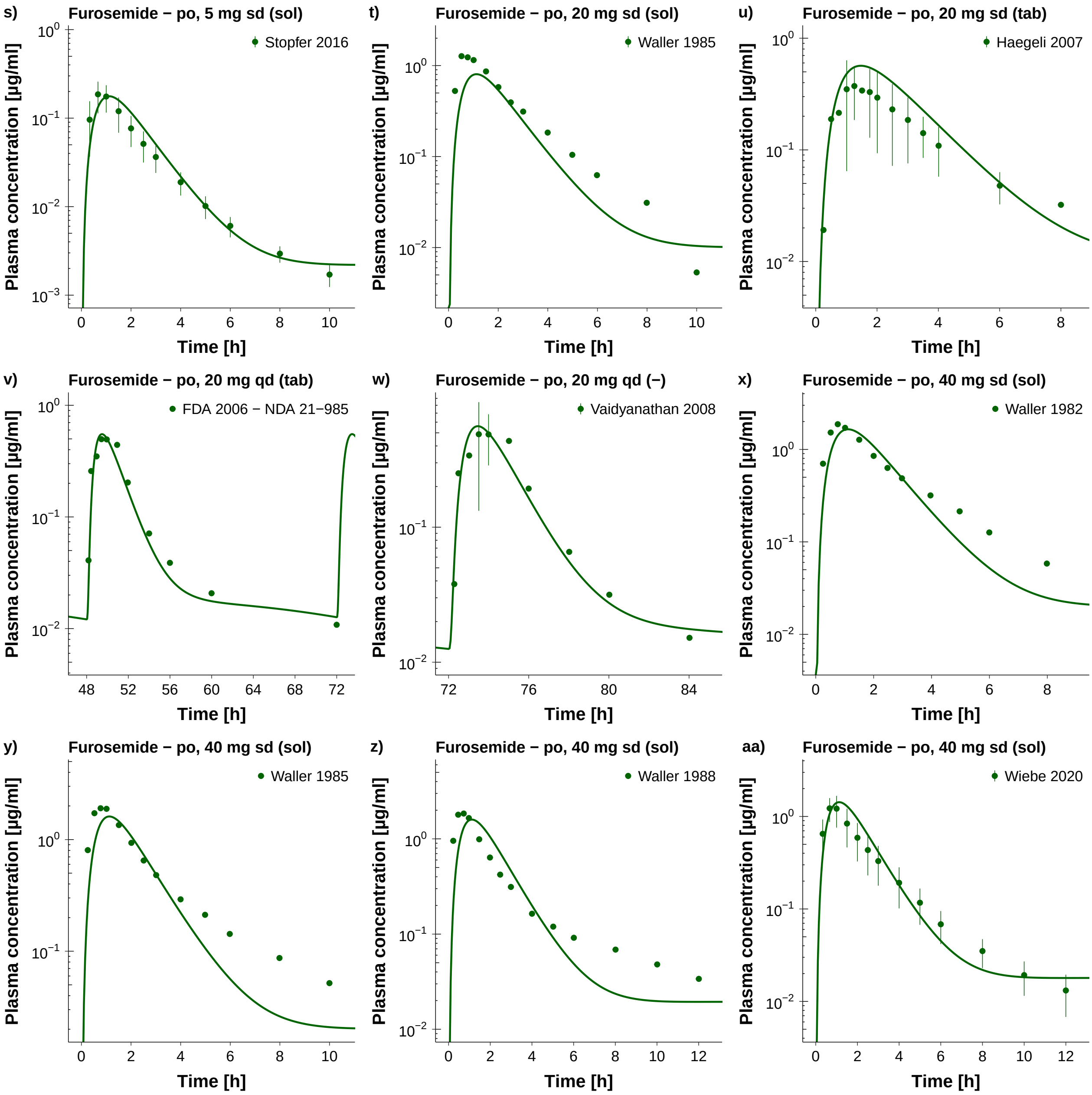

Figure S3.4.3: Furosemide plasma concentration-time profiles. Individual predictions of furosemide plasma concentration-time profiles compared to observed data. Observed data are shown as dots ± standard deviation. Simulations are shown as lines. Details on dosing regimens, study populations and literature references are summarized in Table S3.2.1.

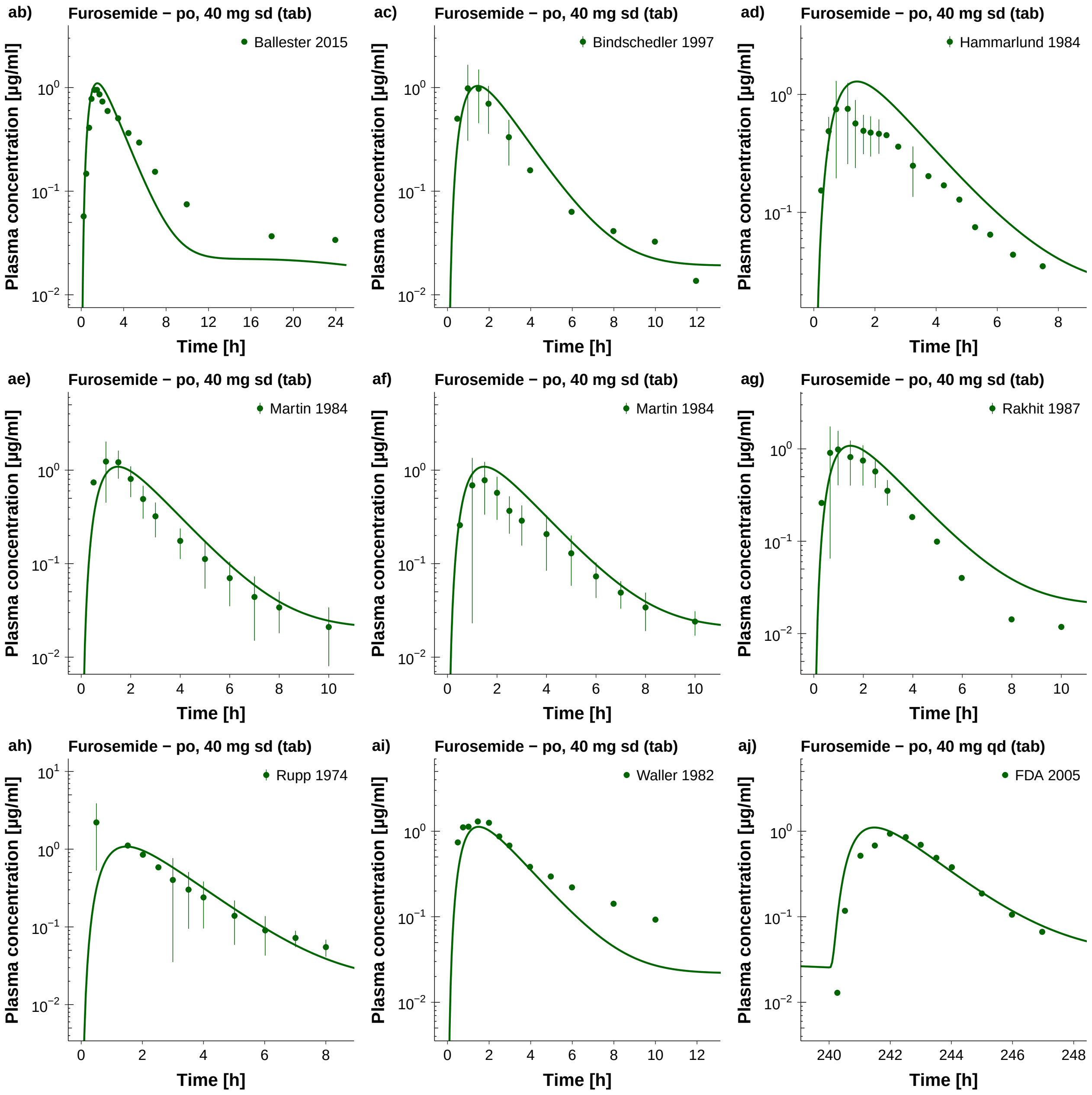

Figure S3.4.3: Furosemide plasma concentration-time profiles. Individual predictions of furosemide plasma concentration-time profiles compared to observed data. Observed data are shown as dots ± standard deviation. Simulations are shown as lines. Details on dosing regimens, study populations and literature references are summarized in Table S3.2.1.

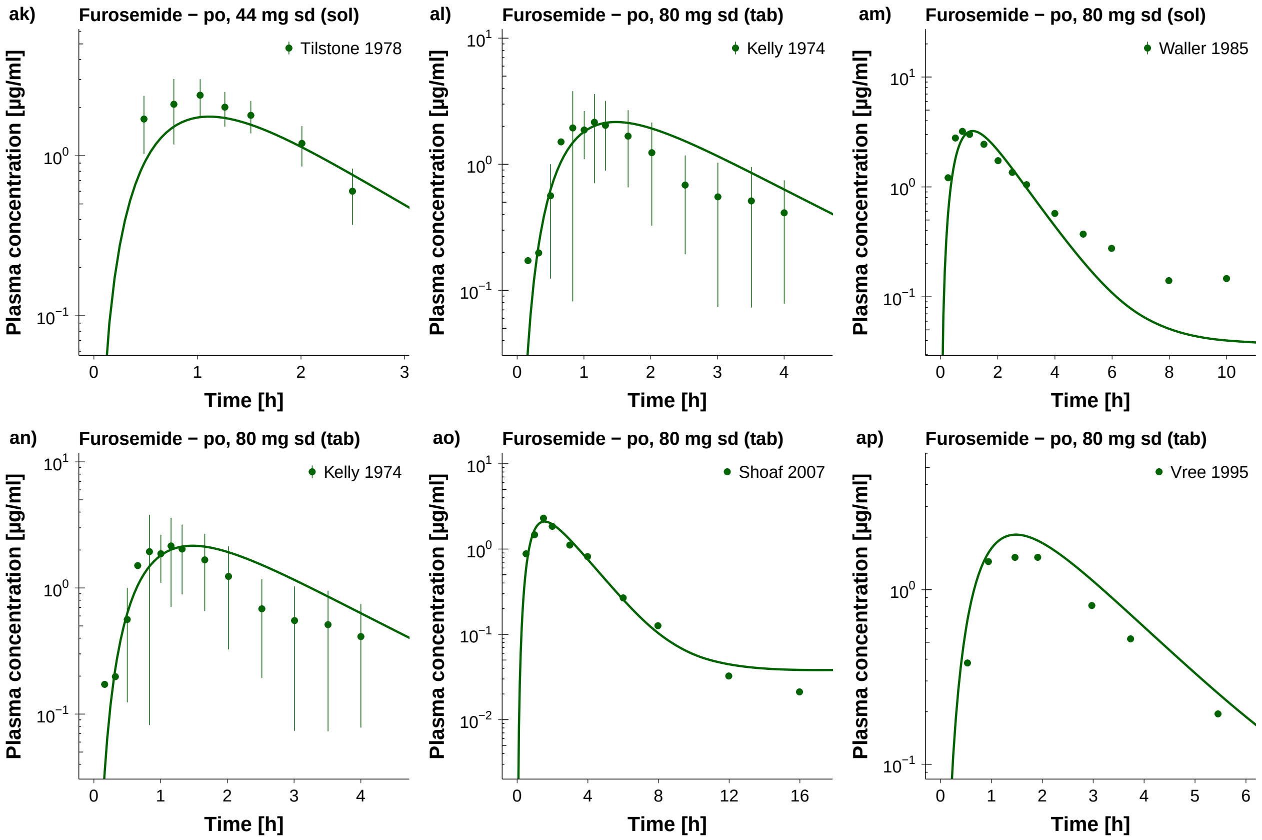

Figure S3.4.3: Furosemide plasma concentration-time profiles. Individual predictions of furosemide plasma concentration-time profiles compared to observed data. Observed data are shown as dots ± standard deviation. Simulations are shown as lines. Details on dosing regimens, study populations and literature references are summarized in Table S3.2.1.

3.4.4 Linear plots - Plasma - Individual predictions

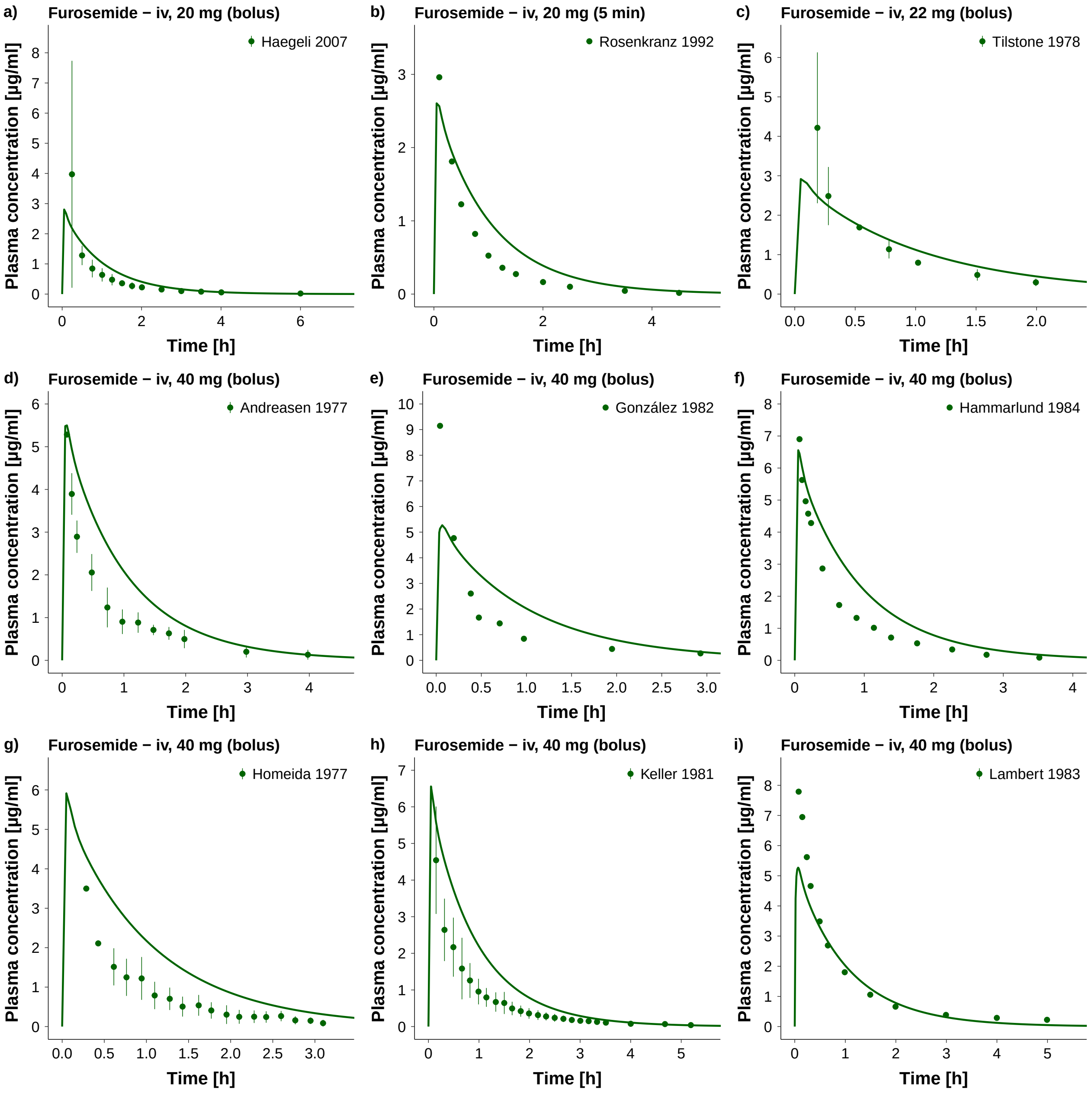

Figure S3.4.4: Furosemide plasma concentration-time profiles. Individual predictions of furosemide plasma concentration-time profiles compared to observed data. Observed data are shown as dots ± standard deviation. Simulations are shown as lines. Details on dosing regimens, study populations and literature references are summarized in Table S3.2.1.

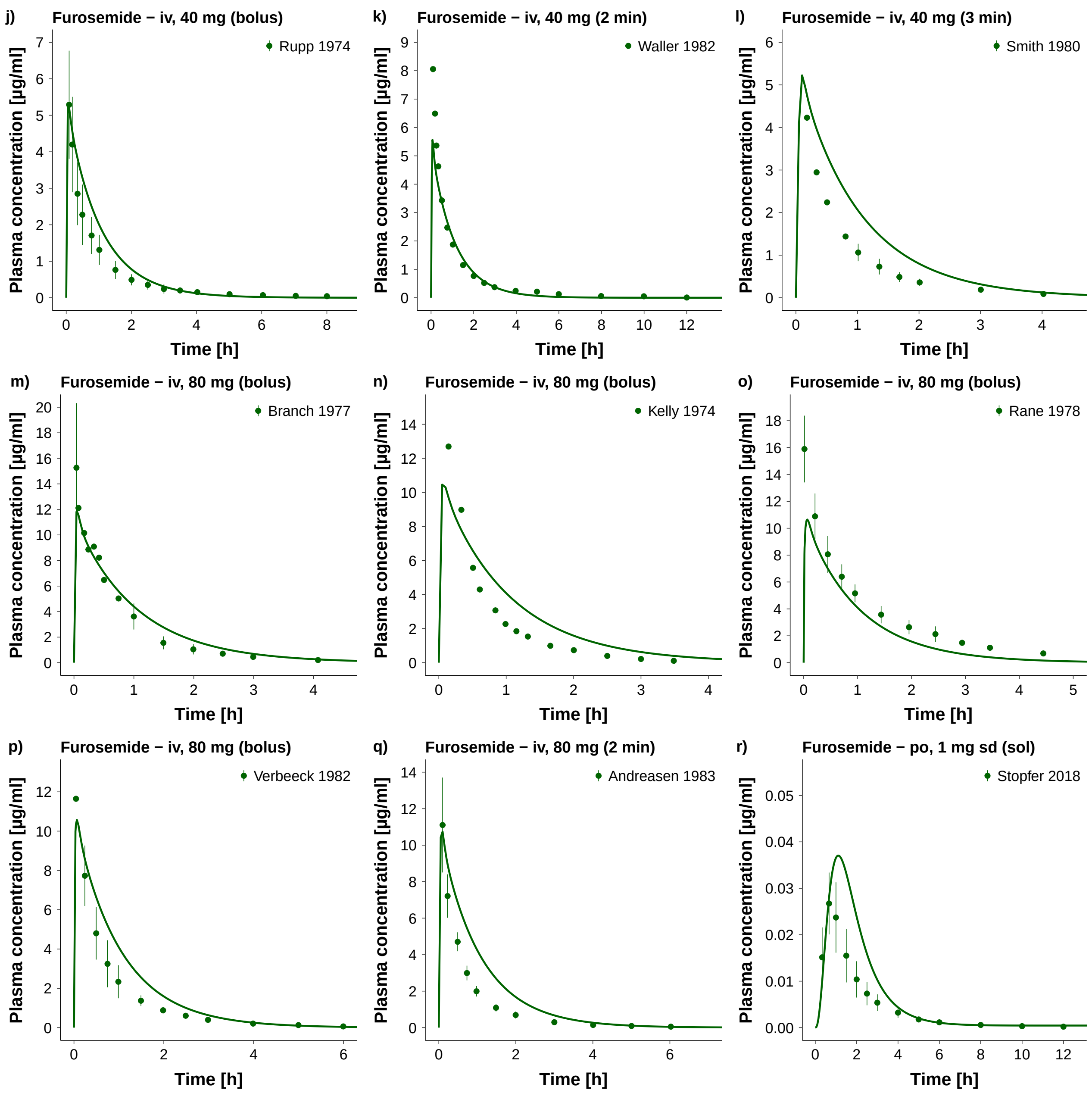

Figure S3.4.4: Furosemide plasma concentration-time profiles. Individual predictions of furosemide plasma concentration-time profiles compared to observed data. Observed data are shown as dots ± standard deviation. Simulations are shown as lines. Details on dosing regimens, study populations and literature references are summarized in Table S3.2.1.

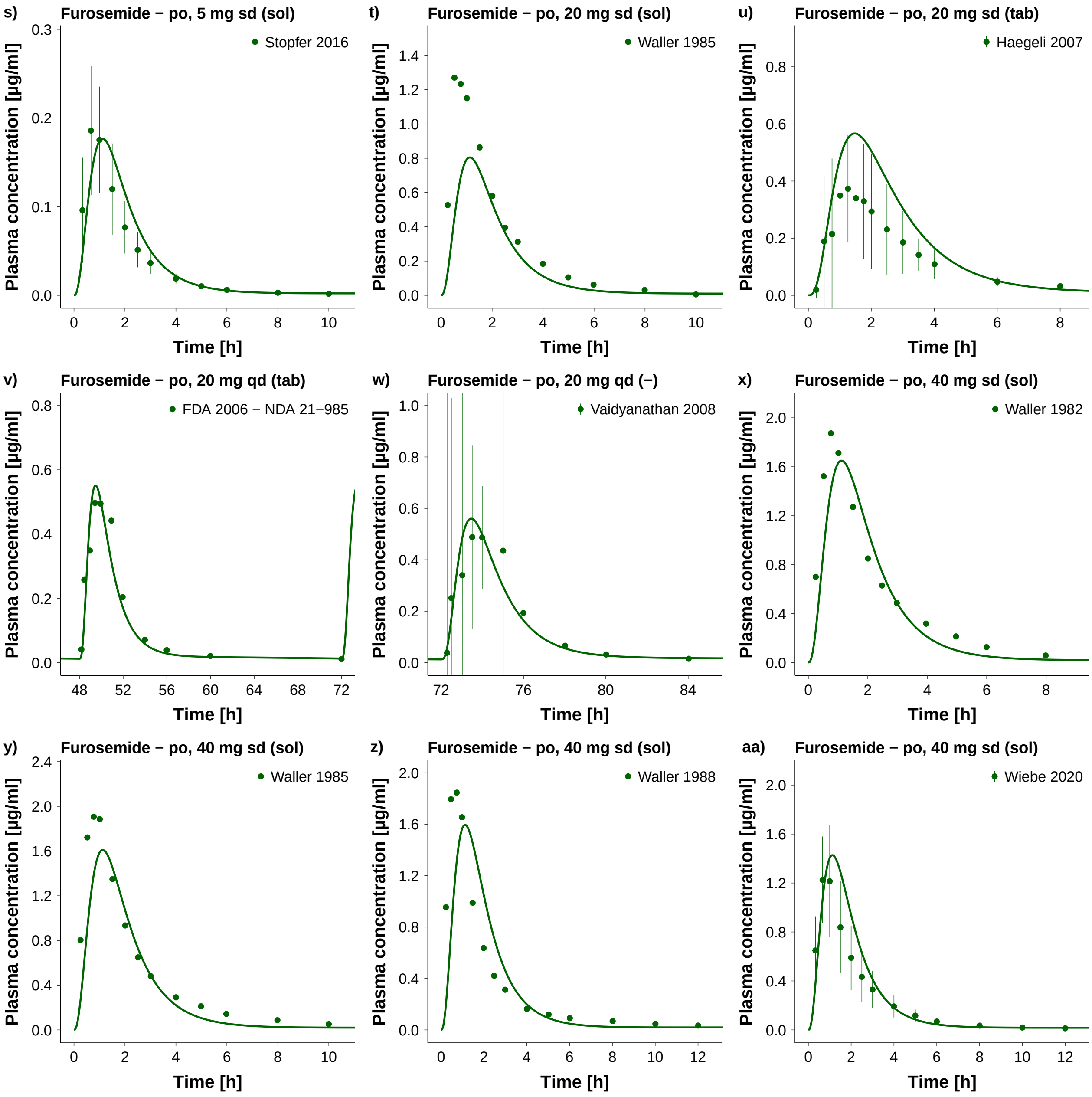

Figure S3.4.4: Furosemide plasma concentration-time profiles. Individual predictions of furosemide plasma concentration-time profiles compared to observed data. Observed data are shown as dots ± standard deviation. Simulations are shown as lines. Details on dosing regimens, study populations and literature references are summarized in Table S3.2.1..

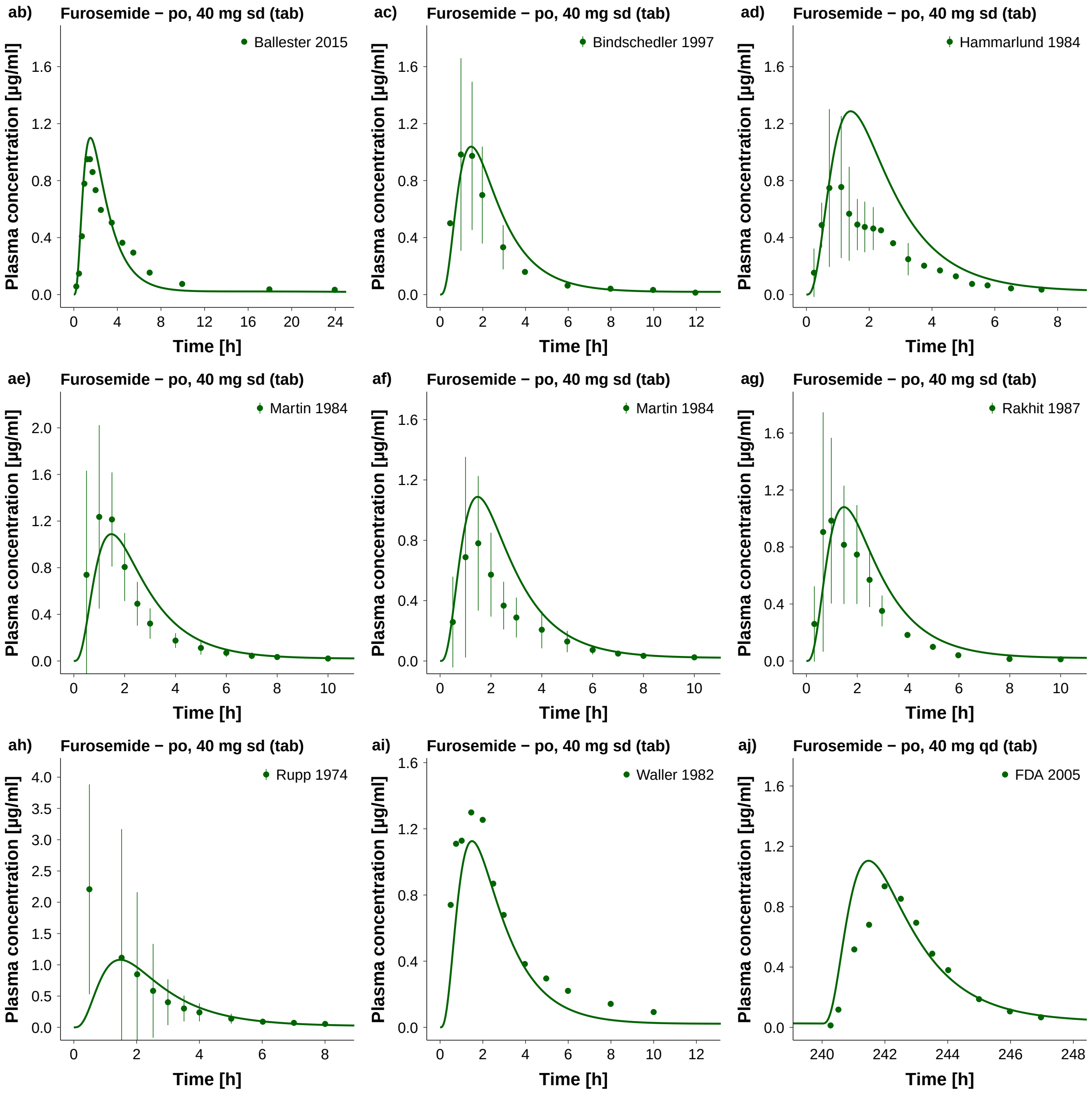

Figure S3.4.4: Furosemide plasma concentration-time profiles. Individual predictions of furosemide plasma concentration-time profiles compared to observed data. Observed data are shown as dots ± standard deviation. Simulations are shown as lines. Details on dosing regimens, study populations and literature references are summarized in Table S3.2.1.

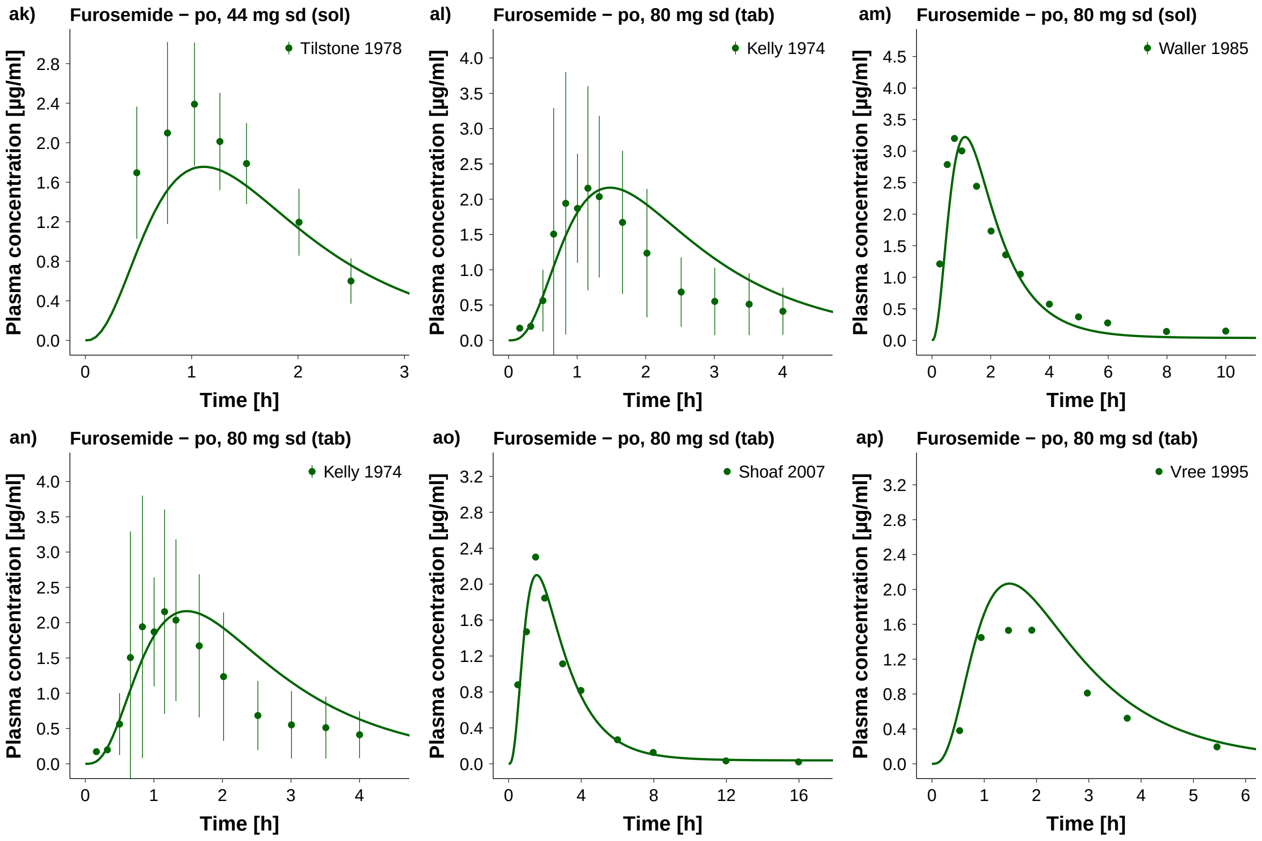

Figure S3.4.4: Furosemide plasma concentration-time profiles. Individual predictions of furosemide plasma concentration-time profiles compared to observed data. Observed data are shown as dots $\pm$ standard deviation. Simulations are shown as lines. Details on dosing regimens, study populations and literature references are summarized in Table S3.2.1.

3.4.5 Linear plots - Fraction excreted unchanged in urine - Population predictions

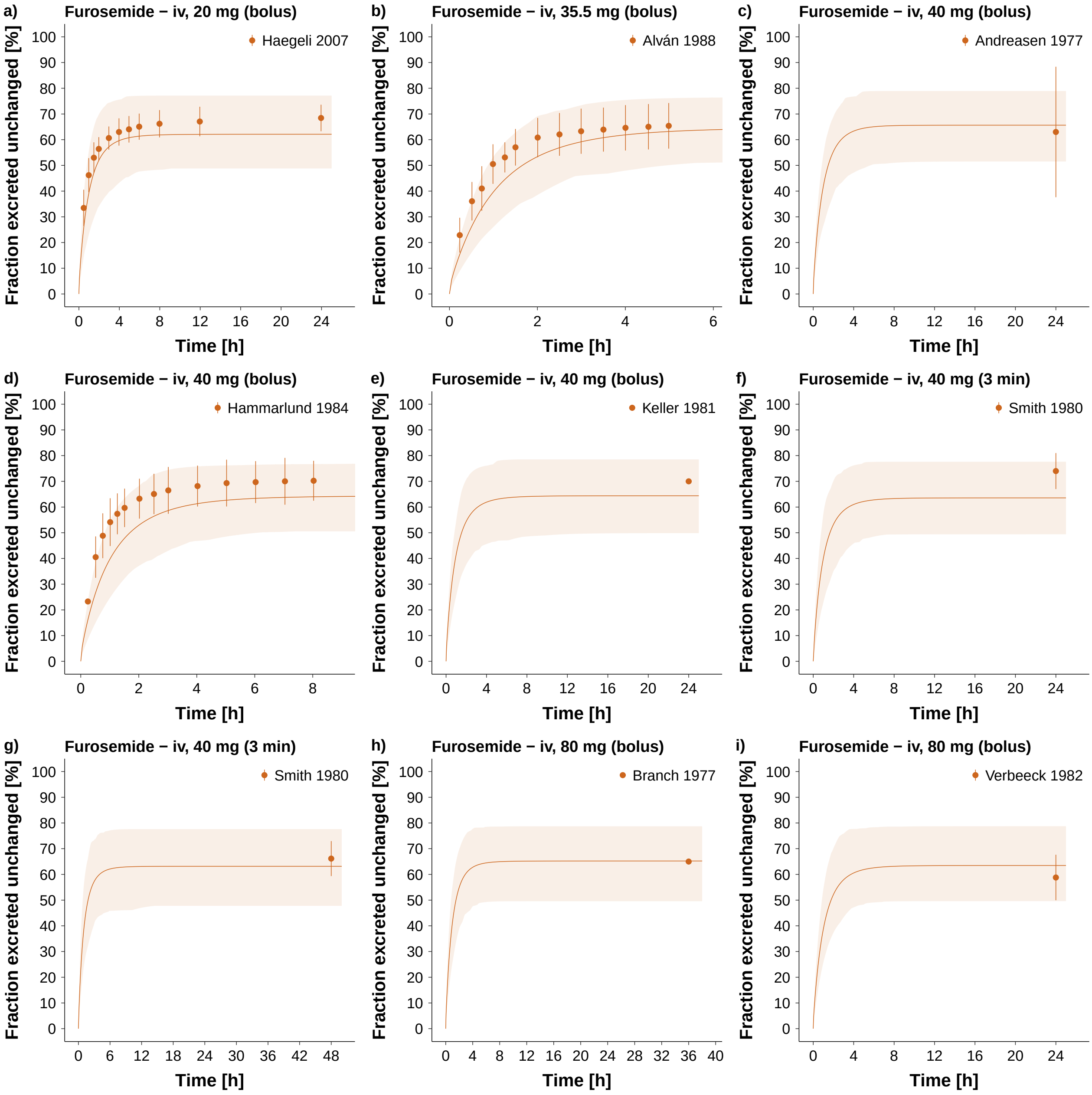

Figure S3.4.5: Furosemide fraction excreted unchanged in urine profiles. Population predictions of furosemide fraction excreted unchanged in urine profiles compared to observed data. Observed data are shown as dots $\pm$ standard deviation. Population simulation arithmetic means are shown as lines; the shaded areas illustrate the predicted population variation ($Q_{16} - Q_{84}$). Details on dosing regimens, study populations and literature references are summarized in Table S3.2.1.

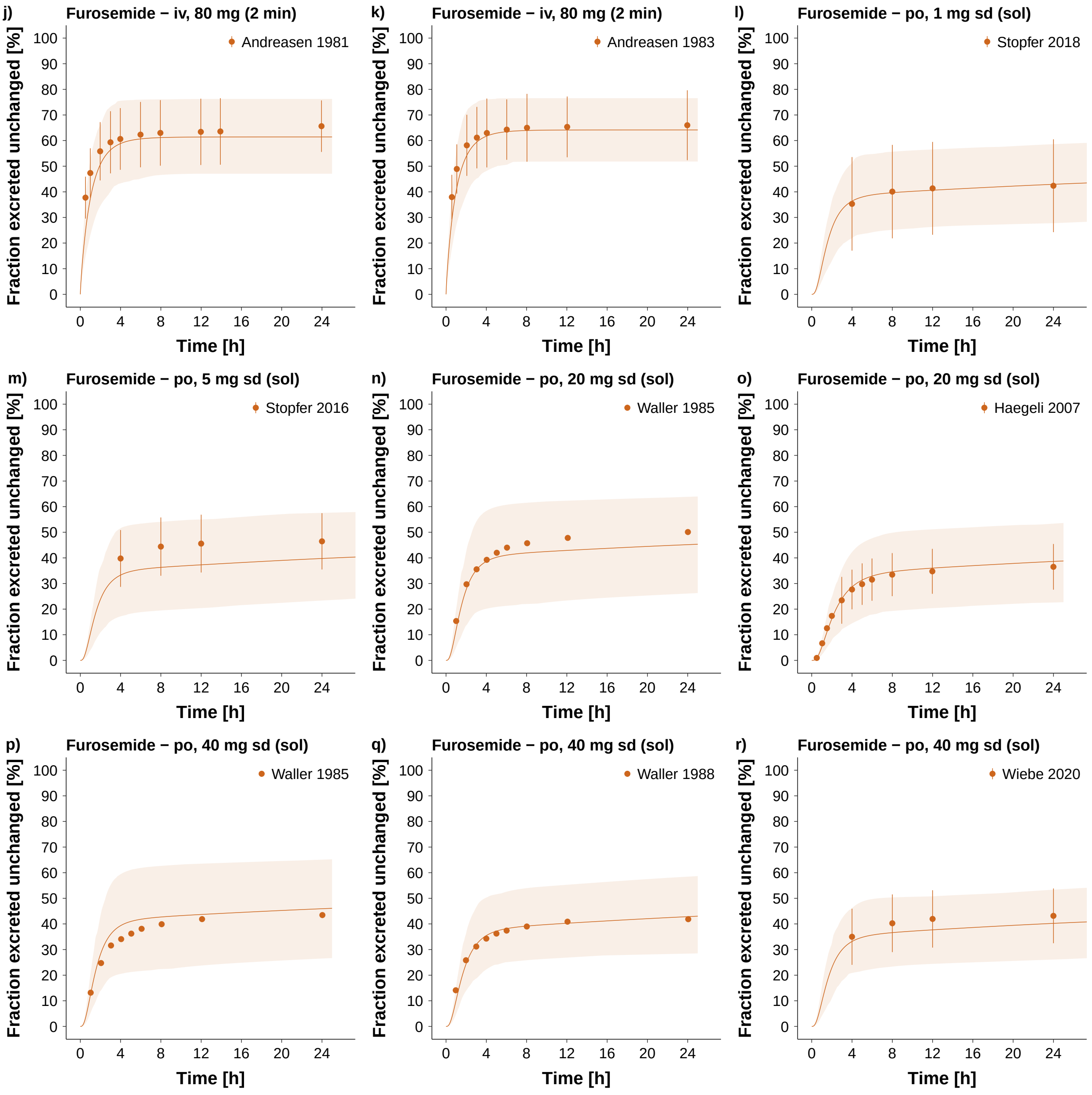

Figure S3.4.5: Furosemide fraction excreted unchanged in urine profiles. Population predictions of furosemide fraction excreted unchanged in urine profiles compared to observed data. Observed data are shown as dots ± standard deviation. Population simulation arithmetic means are shown as lines; the shaded areas illustrate the predicted population variation ($Q_{16} - Q_{84}$). Details on dosing regimens, study populations and literature references are summarized in Table S3.2.1.

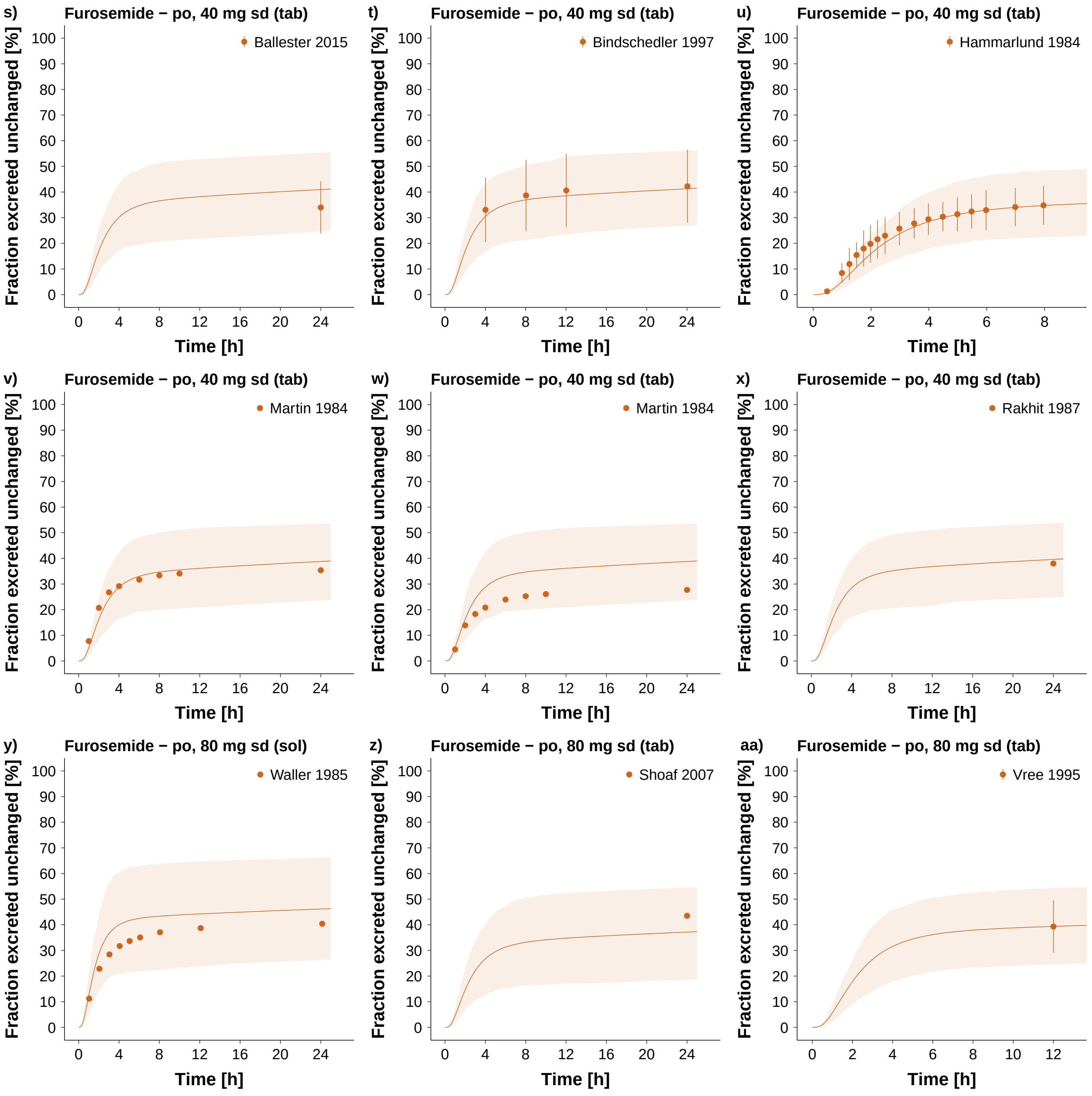

Figure S3.4.5: Furosemide fraction excreted unchanged in urine profiles. Population predictions of furosemide fraction excreted unchanged in urine profiles compared to observed data. Observed data are shown as dots ± standard deviation. Population simulation arithmetic means are shown as lines; the shaded areas illustrate the predicted population variation ($Q_{16} - Q_{84}$). Details on dosing regimens, study populations and literature references are summarized in Table S3.2.1.

3.4.6 Linear plots - Fraction excreted unchanged in urine - Individual predictions

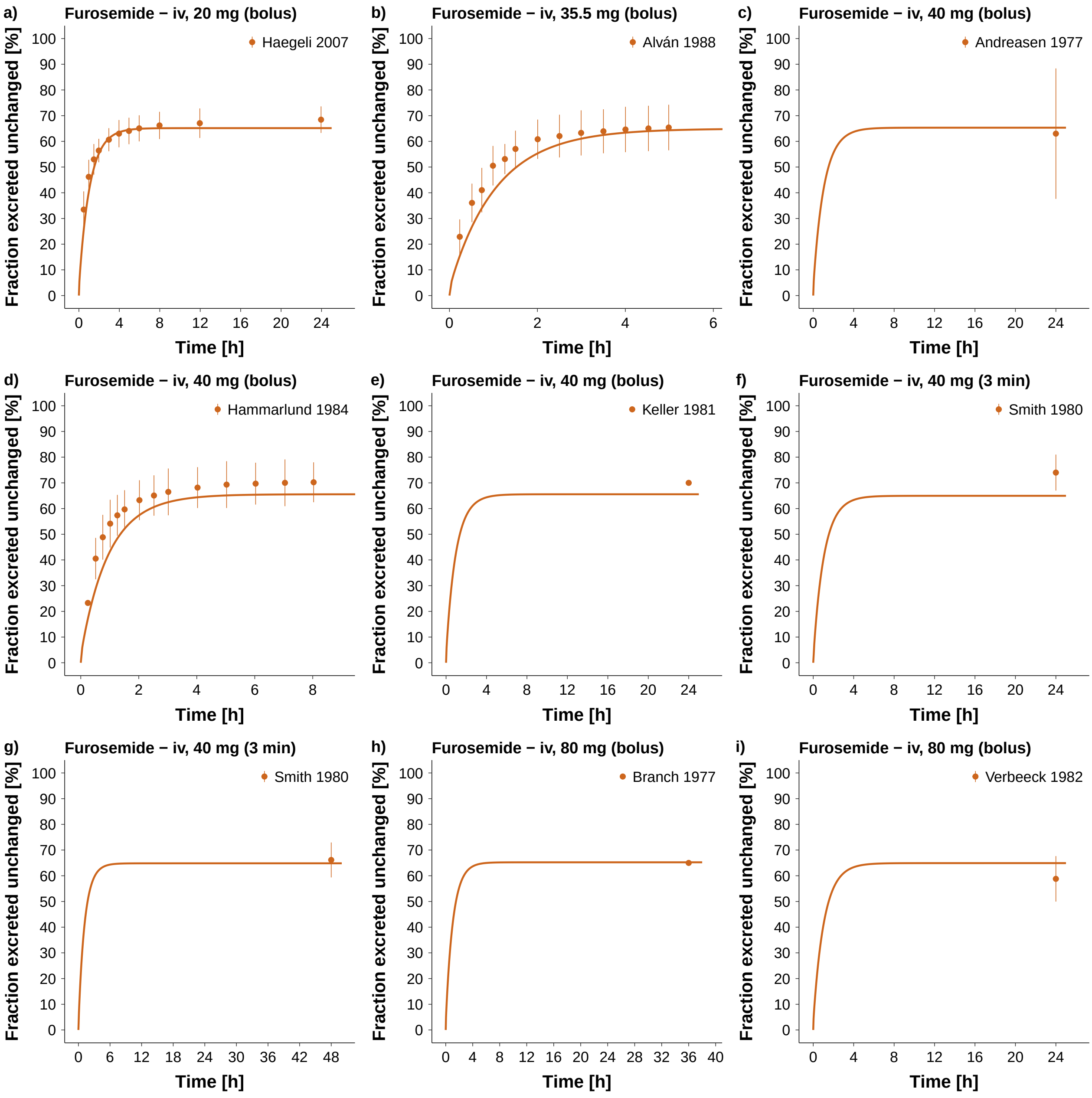

Figure S3.4.6: Furosemide fraction excreted unchanged in urine profiles. Individual predictions of furosemide fraction excreted unchanged in urine profiles compared to observed data. Observed data are shown as dots ± standard deviation. Simulations are shown as lines. Details on dosing regimens, study populations and literature references are summarized in Table S3.2.1.

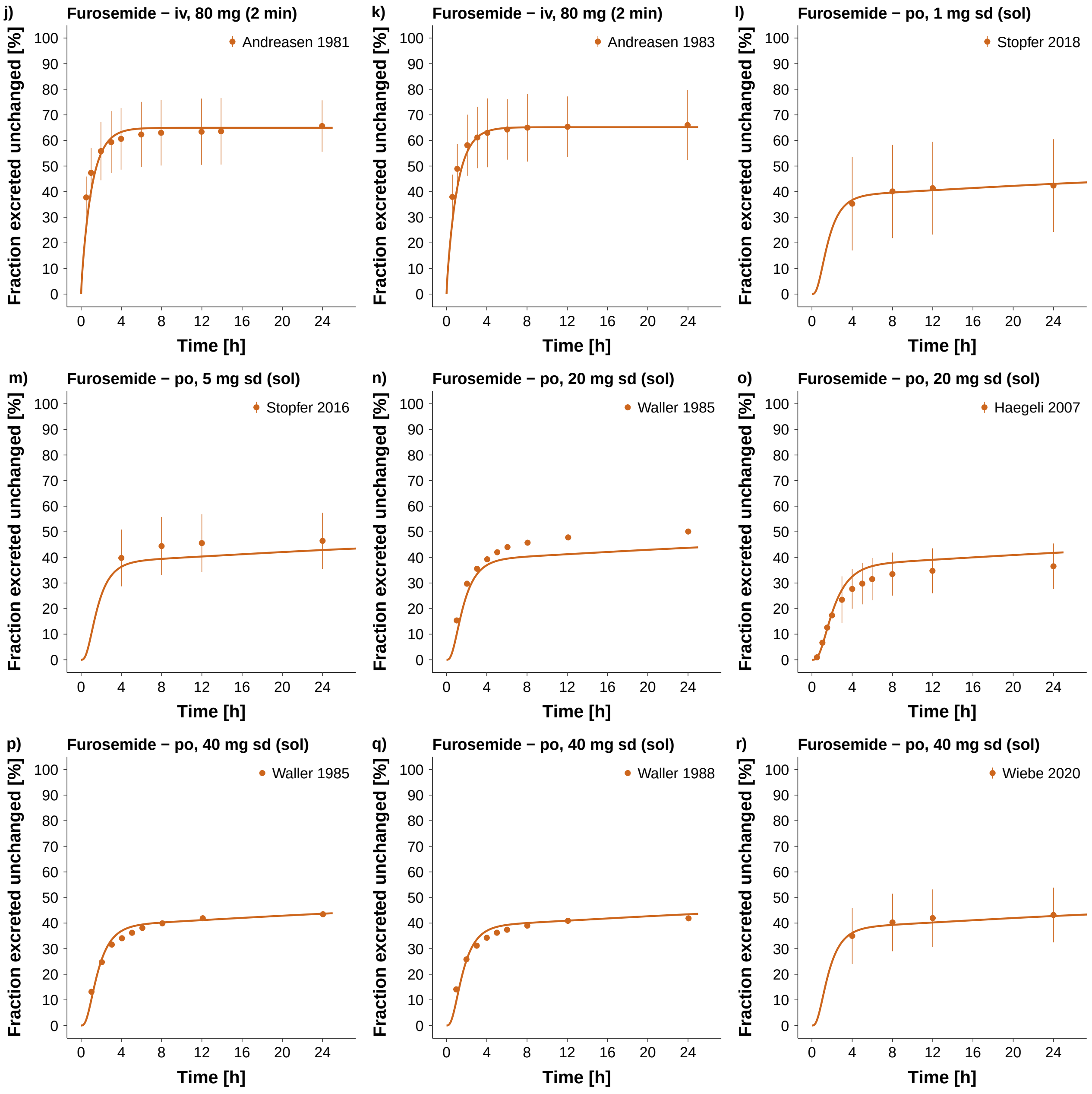

Figure S3.4.6: Furosemide fraction excreted unchanged in urine profiles. Individual predictions of furosemide fraction excreted unchanged in urine profiles compared to observed data. Observed data are shown as dots ± standard deviation. Simulations are shown as lines. Details on dosing regimens, study populations and literature references are summarized in Table S3.2.1.

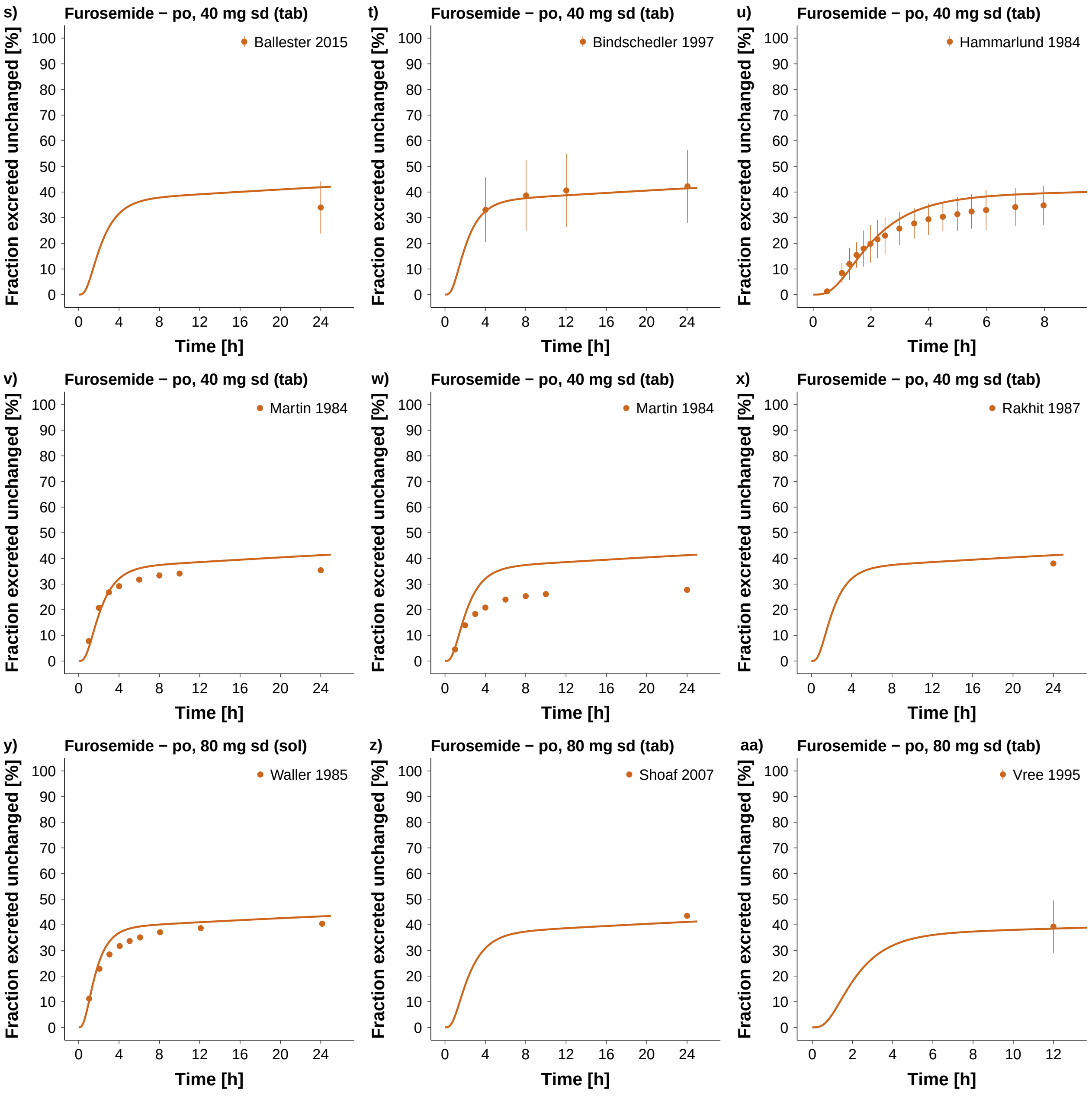

Figure S3.4.6: Furosemide fraction excreted unchanged in urine profiles. Individual predictions of furosemide fraction excreted unchanged in urine profiles compared to observed data. Observed data are shown as dots ± standard deviation. Simulations are shown as lines. Details on dosing regimens, study populations and literature references are summarized in Table S3.2.1.

3.5 Furosemide PBPK model evaluation

3.5.1 Plasma concentration goodness-of-fit plot

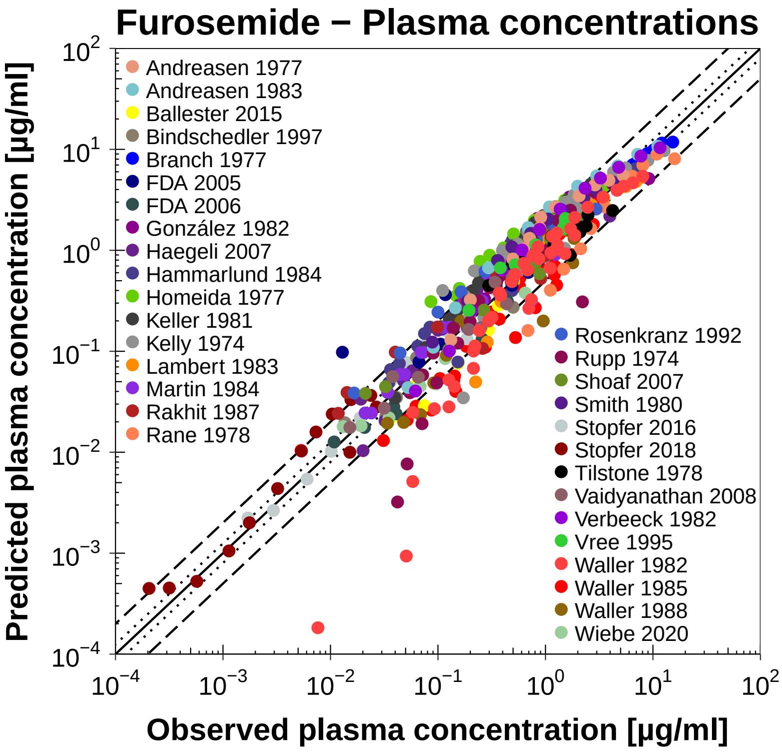

Figure S3.5.1: Furosemide plasma concentrations. Predicted compared to observed furosemide plasma concentration values of all analyzed clinical studies. The solid line marks the line of identity. The dotted lines indicate 1.25-fold, the dashed lines indicate 2-fold deviation. Details on dosing regimens, study populations and literature references are summarized in Table S3.2.1.

3.5.2 Mean relative deviation (MRD) of predicted plasma concentrations

Table S3.5.1: MRD values of predicted furosemide plasma concentrations

Route	Dose [mg]	MRD	Reference
Intravenous			
iv (bolus), sd	20	1.65	Haegeli 2007 [37]
iv (5 min), sd	20	1.94	Rosenkranz 1992 [38]
iv (bolus), sd	22	1.39	Tilstone 1978 [39]
iv (bolus), sd	40	1.70	Andreasen 1977 [41]
iv (bolus), sd	40	1.76	González 1982 [34]
iv (bolus), sd	40	1.68	Hammarlund 1984 [42]
iv (bolus), sd	40	2.43	Homeida 1977 [43]
iv (bolus), sd	40	1.90	Keller 1981 [44]
iv (bolus), sd	40	1.72	Lambert 1983 [35]
iv (bolus), sd	40	2.52	Rupp 1974 [45]
iv (2 min), sd	40	4.63	Waller 1982 [46]
iv (3 min), sd	40	1.75	Smith 1980 [24]
iv (bolus), sd	80	1.34	Branch 1977 [48]
iv (bolus), sd	80	2.03	Kelly 1974 [49]
iv (bolus), sd	80	1.99	Rane 1978 [50]
iv (bolus), sd	80	1.53	Verbeeck 1982 [51]
iv (2 min), sd	80	1.81	Andreasen 1983 [53]
**MRD**		**1.99 (1.34 - 4.63)**<br>**13/17 with MRD $\leq$ 2**	
Oral			
po (sol), sd	1	1.68	Stopfer 2018 [54]
po (sol), sd	5	1.38	Stopfer 2016 [55]
po (sol), sd	20	1.97	Waller 1985 [56]
po (tab), sd	20	1.56	Haegeli 2007 [37]
po (tab), qd	20	1.38	FDA 2006 [57]
po (-), qd	20	1.30	Vaidyanathan 2008 [58]
po (sol), sd	40	1.73	Waller 1982 [46]
po (sol), sd	40	1.91	Waller 1985 [56]
po (sol), sd	40	2.02	Waller 1988 [59]
po (sol), sd	40	1.41	Wiebe 2020 [11]
po (tab), sd	40	1.57	Ballester 2015 [60]
po (tab), sd	40	1.47	Bindschedler 1997 [61]
po (tab), sd	40	1.90	Hammarlund 1984 [42]
po (tab), sd	40	1.54	Martin 1984 [62]
po (tab), sd	40	1.50	Martin 1984 [62]
po (tab), sd	40	1.85	Rakhit 1987 [63]
po (tab), sd	40	1.90	Rupp 1974 [45]
po (tab), sd	40	1.84	Waller 1982 [46]
po (tab), qd	40	2.04	FDA 2005 [64]
po (sol), sd	44	1.38	Tilstone 1978 [39]
po (sol), sd	80	1.55	Kelly 1974 [49]
po (sol), sd	80	1.84	Waller 1985 [56]
po (tab), sd	80	1.79	Kelly 1974 [49]
po (tab), sd	80	1.31	Shoaf 2007 [65]
po (tab), sd	80	1.39	Vree 1995 [33]
**MRD**		**1.65 (1.30 - 2.04)**<br>**23/25 with MRD $\leq$ 2**	
**Overall MRD**		**1.79 (1.30 - 4.63)**<br>**36/42 with MRD $\leq$ 2**	

iv: intravenous, **MRD:** mean relative deviation, **po:** oral, **qd:** once daily, **route:** route of administration, **sd:** single dose, **sol:** solution, **tab:** tablet.

3.5.3 AUC_{last} and C_{max} goodness-of-fit plots

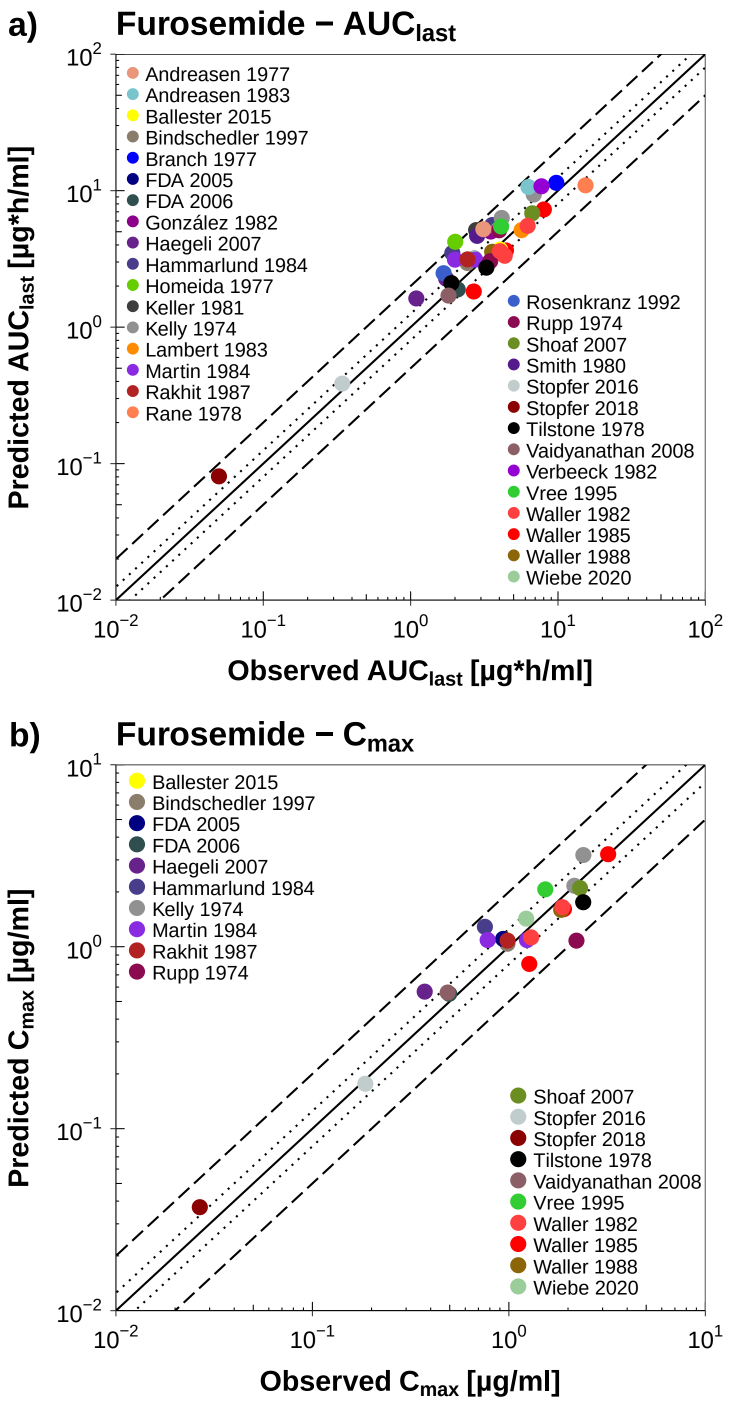

Figure S3.5.2: Furosemide AUC_{last} and C_{max} values. Predicted compared to observed furosemide (a) AUC_{last} and (b) C_{max} values of all analyzed clinical studies. The solid line marks the line of identity. The dotted lines indicate 1.25-fold, the dashed lines indicate 2-fold deviation. Details on dosing regimens, study populations and literature references are summarized in Table S3.2.1. The individual AUC_{last} and C_{max} values, mean GMFE values and ranges are listed in Table S3.5.2.

3.5.4 Predicted and observed AUC_{last} and C_{max} values with mean GMFE values and ranges

Table S3.5.2: Predicted and observed furosemide AUC_{last} and C_{max} values

		AUC_{last}			C_{max}				
**Route**	**Dose [mg]**	**Pred [µg*h/ml]**	**Obs [µg*h/ml]**	**Pred/Obs**	**Pred [µg/ml]**	**Obs [µg/ml]**	**Pred/Obs**	**t_{last} [h]**	**Reference**
Intravenous									
iv (bolus), sd	20	2.27	1.74	1.30	-	-	-	6.0	Haegeli 2007 [37]
iv (5 min), sd	20	2.49	1.67	1.49	-	-	-	4.5	Rosenkranz 1992 [38]
iv (bolus), sd	22	2.10	1.89	1.11	-	-	-	2.0	Tilstone 1978 [39]
iv (bolus), sd	40	5.22	3.12	1.68	-	-	-	4.0	Andreasen 1977 [41]
iv (bolus), sd	40	5.02	3.49	1.44	-	-	-	3.0	González 1982 [34]
iv (bolus), sd	40	5.60	3.58	1.57	-	-	-	4.5	Hammarlund 1984 [42]
iv (bolus), sd	40	4.22	2.01	2.10	-	-	-	3.0	Homeida 1977 [43]
iv (bolus), sd	40	5.14	2.78	1.85	-	-	-	5.0	Keller 1981 [44]
iv (bolus), sd	40	5.14	5.66	0.91	-	-	-	5.0	Lambert 1983 [35]
iv (bolus), sd	40	5.13	3.98	1.29	-	-	-	8.0	Rupp 1974 [45]
iv (2 min), sd	40	5.53	6.23	0.89	-	-	-	12.0	Waller 1982 [46]
iv (3 min), sd	40	4.67	2.83	1.65	-	-	-	4.0	Smith 1980a [24]
iv (bolus), sd	80	11.42	9.74	1.17	-	-	-	4.0	Branch 1977 [48]
iv (bolus), sd	80	9.37	6.84	1.37	-	-	-	3.5	Kelly 1974 [49]
iv (bolus), sd	80	10.95	15.37	0.71	-	-	-	4.5	Rane 1978 [50]
iv (bolus), sd	80	10.77	7.72	1.39	-	-	-	6.0	Verbeeck 1982 [51]
iv (2 min), sd	80	10.64	6.30	1.69	-	-	-	6.0	Andreasen 1983 [53]
**GMFE**				**1.45 (1.10 - 2.10)**					
				**16/17 with GMFE $\leq$ 2**					
Oral									
po (sol), sd	1	0.08	0.05	1.61	0.04	0.03	1.39	12.0	Stopfer 2018 [54]
po (sol), sd	5	0.39	0.34	1.12	0.18	0.19	0.95	10.0	Stopfer 2016 [55]
po (sol), sd	20	1.83	2.68	0.68	0.80	1.27	0.63	10.0	Waller 1985 [56]

AUC_{last}: area under the plasma concentration-time curve (AUC) from the time of drug administration to the time of the last concentration measurement, **C_{max}:** peak plasma concentration, **GMFE:** geometric mean fold error, **iv:** intravenous, **obs:** observed, **po:** oral, **pred:** predicted, **qd:** once daily, **route:** route of administration, **sd:** single dose, **sol:** solution, **tab:** tablet, **t_{last}:** time of the last concentration measurement. GMFE values are means and ranges.

Table S3.5.2: Predicted and observed furosemide AUC_{last} and C_{max} values *(continued)*

		AUC_{last}			C_{max}				
**Route**	**Dose [mg]**	**Pred [µg*h/ml]**	**Obs [µg*h/ml]**	**Pred/Obs**	**Pred [µg/ml]**	**Obs [µg/ml]**	**Pred/Obs**	**t_{last} [h]**	**Reference**
po (tab), sd	20	1.62	1.09	1.49	0.57	0.37	1.52	8.0	Haegeli 2007 [37]
po (tab), qd	20	1.89	2.09	0.91	0.55	0.50	1.11	24.0	FDA 2006 [57]
po (-), qd	20	1.70	1.81	0.94	0.56	0.49	1.15	12.0	Vaidyanathan 2008 [58]
po (sol), sd	40	3.60	4.04	0.89	1.65	1.87	0.88	8.0	Waller 1982 [46]
po (sol), sd	40	3.65	4.43	0.82	1.61	1.91	0.84	10.0	Waller 1985 [56]
po (sol), sd	40	3.57	3.57	1.00	1.60	1.85	0.86	12.0	Waller 1988 [59]
po (sol), sd	40	3.17	2.70	1.17	1.43	1.23	1.16	12.0	Wiebe 2020 [11]
po (tab), sd	40	3.70	4.04	0.92	1.10	0.95	1.16	24.0	Ballester 2015 [60]
po (tab), sd	40	2.95	2.43	1.22	1.04	0.98	1.06	12.0	Bindschedler 1997 [61]
po (tab), sd	40	3.50	1.92	1.82	1.29	0.75	1.70	7.5	Hammarlund 1984 [42]
po (tab), sd	40	3.12	2.74	1.14	1.09	1.24	0.88	10.0	Martin 1984 [62]
po (tab), sd	40	3.12	2.00	1.56	1.09	0.78	1.39	10.0	Martin 1984 [62]
po (tab), sd	40	3.14	2.43	1.29	1.08	0.98	1.10	10.0	Rakhit 1987 [63]
po (tab), sd	40	3.04	3.47	0.88	1.08	2.21	0.49	8.0	Rupp 1974 [45]
po (tab), sd	40	3.33	4.35	0.77	1.13	1.30	0.87	10.0	Waller 1982 [46]
po (tab), qd	40	3.18	2.71	1.18	1.10	0.94	1.18	10.0	FDA 2005 [64]
po (sol), sd	44	2.73	3.26	0.84	1.76	2.39	0.73	2.5	Tilstone 1978 [39]
po (sol), sd	80	6.31	4.15	1.52	3.19	2.39	1.33	4.0	Kelly 1974 [49]
po (sol), sd	80	7.29	8.02	0.91	3.22	3.20	1.01	10.0	Waller 1985 [56]
po (tab), sd	80	5.20	3.94	1.32	2.16	2.16	1.00	4.0	Kelly 1974 [49]
po (tab), sd	80	6.83	6.69	1.02	2.10	2.30	0.91	16.0	Shoaf 2007 [65]
po (tab), sd	80	5.46	4.11	1.33	2.07	1.53	1.35	5.5	Vree 1995 [33]
**GMFE**				**1.26 (1.00 - 1.82)**			**1.26 (1.00 - 2.04)**		
				**25/25 with GMFE ≤ 2**			**24/25 with GMFE ≤ 2**		
**Overall GMFE**				**1.34 (1.00 - 2.10)**			**1.26 (1.00 - 2.04)**		
				**41/42 with GMFE ≤ 2**			**24/25 with GMFE ≤ 2**		

AUC_{last}: area under the plasma concentration-time curve (AUC) from the time of drug administration to the time of the last concentration measurement, **C_{max}:** peak plasma concentration, **GMFE:** geometric mean fold error, **iv:** intravenous, **obs:** observed, **po:** oral, **pred:** predicted, **qd:** once daily, **route:** route of administration, **sd:** single dose, **sol:** solution, **tab:** tablet, **t_{last}:** time of the last concentration measurement. GMFE values are means and ranges.

3.5.5 Sensitivity analysis

Sensitivity of the final furosemide PBPK model to single parameters (local sensitivity analysis) was analyzed, measured as relative change of the $AUC_{0\text{-}24}$ of the last administration interval for simulation of the highest recommended furosemide dose (80 mg once daily for 14 days).
The sensitivity analysis results are illustrated in Figure S3.5.3. Parameters were included into the analysis if they were optimized (OAT3 catalytic rate constant, UGT1A9 catalytic rate constant, MRP4 catalytic rate constant, 50% dissolution time, transcellular intestinal permeability, paracellular intestinal permeability), if they are associated with optimized parameters (OAT3 Michaelis-Menten constant, UGT1A9 Michaelis-Menten constant, MRP4 Michaelis-Menten constant, dissolution shape) or if they might have a strong impact due to calculation methods used in the model (solubility, fraction unbound in plasma, blood/plasma concentration ratio, lipophilicity, glomerular filtration rate fraction). Applying a threshold of 0.5, the furosemide model is sensitive to the values of the furosemide fraction unbound in plasma (literature value) and the OAT3 catalytic rate constant (optimized).

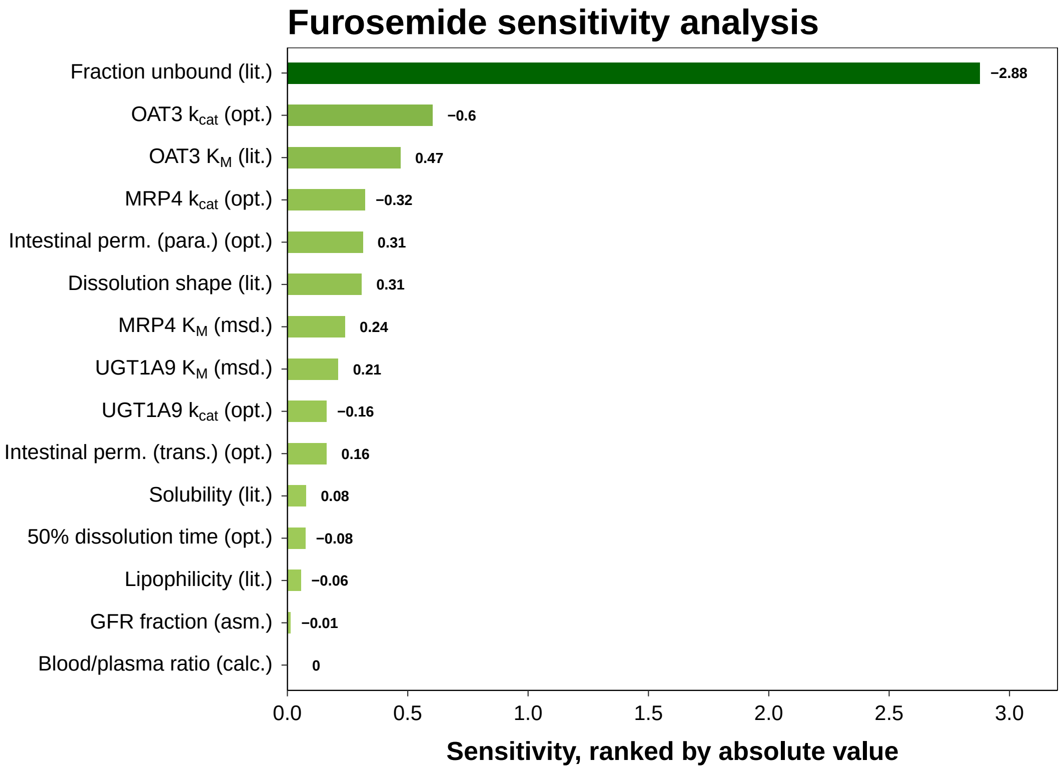

Figure S3.5.3: Furosemide PBPK model sensitivity analysis. Sensitivity of the model to single parameters, measured as change of the simulated $AUC_{0\text{-}24}$ under steady-state conditions of an 80 mg once daily furosemide regimen. A sensitivity value of +0.5 signifies that a 100% increase of the examined parameter causes a 50% increase of the simulated AUC. **Asm.:** assumed, **calc.:** calculated, **GFR:** glomerular filtration rate, **intestinal perm. (para.):** paracellular intestinal permeability, **intestinal perm. (trans.):** transcellular intestinal permeability, **k_{cat}:** catalytic rate constant, **K_M:** Michaelis-Menten constant, **MRP4:** multidrug resistance-associated protein 4, **msd.:** measured, **lit.:** literature, **OAT3:** organic anion transporter 3, **opt.:** optimized, **UGT1A9:** uridine 5'-diphospho-glucuronosyltransferase 1A9.

4 PBPK modeling of rifampicin

4.1 Rifampicin PBPK model

Rifampicin is an antibiotic used for the treatment of mycobacterium infections. It induces the expression of multiple metabolizing enzymes and transporters and is classified by the FDA as strong clinical index inducer of cytochrome P450 (CYP) 2C19 and CYP3A4 [19]. The rifampicin PBPK model applied for DDI prediction has been developed and qualified previously [81–85]. Drug-dependent parameters of the rifampicin model used to describe the probenecid-rifampicin DDI are summarized in Table S4.2.1

4.2 Drug-dependent parameters

Table S4.2.1: Drug-dependent parameters of the rifampicin PBPK model (adopted from [81])

Parameter	Value	Unit	Source	Literature	Reference	Description
MW	822.94	g/mol	Literature	822.94	[26]	Molecular weight
pKa (acid)	1.70	-	Literature	1.70	[86]	Acid dissociation constant
pKa (base)	7.90	-	Literature	7.90	[86]	Acid dissociation constant
Solubility (pH 7.50)	2.80	mg/ml	Literature	1.10 (pH 6.50), 1.40 (pH 6.80), 2.54 (pH 6.80), 2.80 (pH 7.50), 3.35 (pH 7.40)	[87–90]	Solubility
logP	2.50	-	Optimized	1.30, 2.70	[26, 87]	Lipophilicity
fu	17.00	%	Literature	11.00, 16.00, 17.00, 17.50	[87, 90–92]	Fraction unbound in plasma
B/P ratio	0.89	-	Calculated	0.90[a]	[93]	Blood/plasma ratio
OATP1B1 K_M	1.50	µmol/l	Literature	1.50	[94]	OATP1B1 Michaelis-Menten constant
OATP1B1 k_{cat}	105.41	1/min	Optimized	-	-	OATP1B1 transport rate constant
AADAC K_M	195.10	µmol/l	Literature	195.10	[95]	AADAC Michaelis-Menten constant
AADAC k_{cat}	9.87	1/min	Optimized	-	-	AADAC catalytic rate constant
Pgp K_M	55.00	µmol/l	Literature	55.00	[96]	Pgp Michaelis-Menten constant
Pgp k_{cat}	0.61	1/min	Optimized	-	-	Pgp transport rate constant
GFR fraction	1.00	-	Assumed	-	-	Fraction of filtered drug in the urine
EHC continuous fraction	1.00	-	Assumed	-	-	Fraction of bile continually released
Induction EC_{50}	0.34	µmol/l	Literature	0.34	[91, 92]	Conc. for half-maximal induction
E_{max} OATP1B1	0.38	-	Optimized	-	-	Maximum in vivo induction effect
E_{max} AADAC	0.99	-	Optimized	-	-	Maximum in vivo induction effect
E_{max} Pgp	2.50	-	Literature	2.50	[97]	Maximum in vivo induction effect
OATP1B1 K_i	0.48	µmol/l	Literature	0.48	[98]	Conc. for half-maximal competitive inhibition
Pgp K_i	169.00	µmol/l	Literature	169.00	[99]	Conc. for half-maximal competitive inhibition
Partition coefficients	Diverse	-	Calculated	R&R	[100, 101]	Cell to plasma partition coefficients
Cellular permeability	2.93E-5	cm/min	Calculated	PK-Sim	[102]	Permeability into the cellular space
Intestinal permeability	1.24E-5	cm/min	Optimized	3.84E-7	Calculated	Transcellular intestinal permeability

AADAC: arylacetamide deacetylase, **conc.:** concentration, **EHC:** enterohepatic circulation, **GFR:** glomerular filtration rate, **OATP1B1:** organic anion transporting polypeptide 1B1, **Pgp:** P-glycoprotein, **PK-Sim:** PK-Sim standard calculation method, **R&R:** Rodgers and Rowland calculation method.

[a] blood/serum concentration ratio measured in tuberculosis patients

5 Probenecid-furosemide DDI

5.1 PBPK DDI modeling

To predict the probenecid-furosemide DDI, competitive inhibition of OAT3 by probenecid was implemented using a K_i of 5.41 µmol/l. This probenecid OAT3 K_i was determined in human embryonic kidney 293 cells (HEK293) expressing hOAT3, with furosemide as the substrate (0.25 µmol/l) and 0-100 µmol/l of probenecid [9]. Non-competitive inhibition of UGT1A9 was implemented using a K_i of 242.0 µmol/l. This probenecid UGT1A9 K_i was determined using Corning® Supersomes™ Human UGT1A9, with furosemide as the substrate (18-72 µmol/l) and 0-3000 µmol/l of probenecid [in-house measurement]. The K_i value to model the competitive inhibition of MRP4 (K_i = 87.40 µmol/l) was optimized during parameter identification of the furosemide PBPK model, due to a lack of in vitro data.
Details on dosing regimens, study populations and literature references of the predicted clinical DDI studies are summarized in Table S5.2.1. Plots of population predicted compared to observed furosemide plasma concentration-time profiles of all probenecid-furosemide DDI studies are shown in semilogarithmic (Figure S5.3.1) and linear plots (Figure S5.3.2). Plots of individual predicted compared to observed furosemide plasma concentration-time profiles of all probenecid-furosemide DDI studies are shown in semilogarithmic (Figure S5.3.3) and linear plots (Figure S5.3.4). Population predicted compared to observed fraction excreted unchanged in urine profiles are shown Figure S5.3.5. Individual predicted compared to observed fraction excreted unchanged in urine profiles are shown Figure S5.3.6. The correlation of predicted to observed DDI AUC_{last} ratios and DDI C_{max} ratios is shown in Figure S5.4.1. The individual DDI AUC_{last} ratios and DDI C_{max} ratios with mean GMFE values and ranges are summarized in Table S5.4.1.

5.2 Clinical studies

Table S5.2.1: Probenecid-furosemide DDI study table

Probenecid administration		Furosemide administration		Dose gap [h]	n	Age [years]	Weight [kg]	Height [cm]	Females [%]	Dataset	Reference
Dose [mg]	***Route***	***Dose [mg]***	***Route***								
500	po (-), qid (D1-D3)	40	iv (bolus), sd (D4)	2	6	20-25	-	-	0	test	Homeida 1977 [43]
1000	po (-), sd (D4)										
1000	po (tab), bid (D1)	40	iv (3 min), sd (D1)	1	4	21-33	65-77	-	0	test	Smith 1980 [24]
1000	po (tab), bid (D1)	1	po (sol), sd (D1)	1	15	33 (21-51)	79 (62-95)	180 (169-191)	0	test	Wiebe 2020 [11]
1000	po (tab), bid (D1)	40	po (sol), sd (D1)	1	15	34 (21-52)	79 (62-95)	179 (169-191)	0	training	Wiebe 2020 [11]
1000	po (tab), sd (D1)	40	po (tab), sd (D1)	1	14	26 ± 4	63 ± 7	-	0	test	Shen 2019 [23]
1000	po (tab), sd (D1)	80	po (tab), sd (D1)	1	9	35 ± 6	78 ± 8	-	33	test	Vree 1995 [33]

bid: twice daily, **D:** day of administration, **iv:** intravenous, **n:** number of individuals studied, **po:** oral, **qid:** four times daily, **route:** route of administration, **sd:** single dose, **sol:** solution, **tab:** tablet, **test:** test dataset (model evaluation), **training:** training dataset (parameter optimization). Values are means ± standard deviation or ranges.

5.3 Profiles

5.3.1 Semilogarithmic plots - Plasma - Population predictions

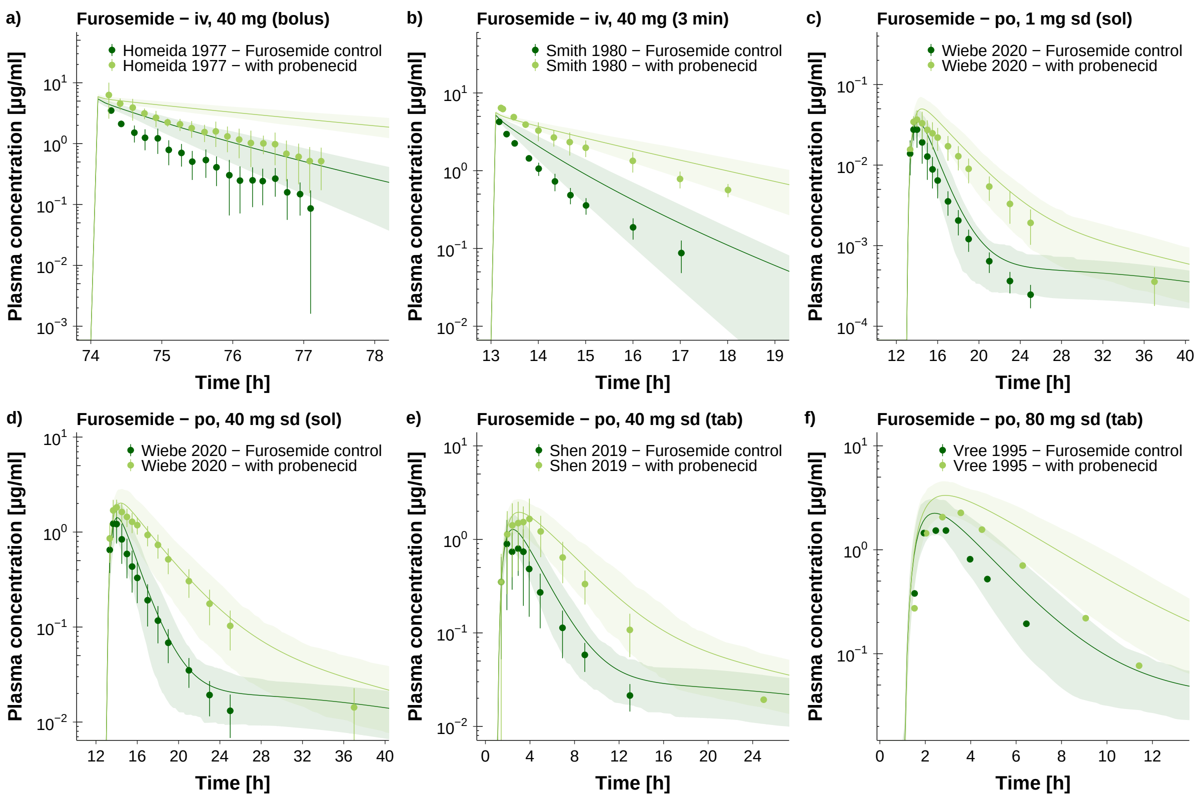

Figure S5.3.1: Probenecid-furosemide DDI. Population predictions of furosemide plasma concentration-time profiles compared to observed data. Observed data are shown as dots ± standard deviation. Population simulation arithmetic means are shown as lines; the shaded areas illustrate the predicted population variation ($Q_{16} - Q_{84}$). Details on dosing regimens, study populations and literature references are summarized in Table S5.2.1.

5.3.2 Linear plots - Plasma - Population predictions

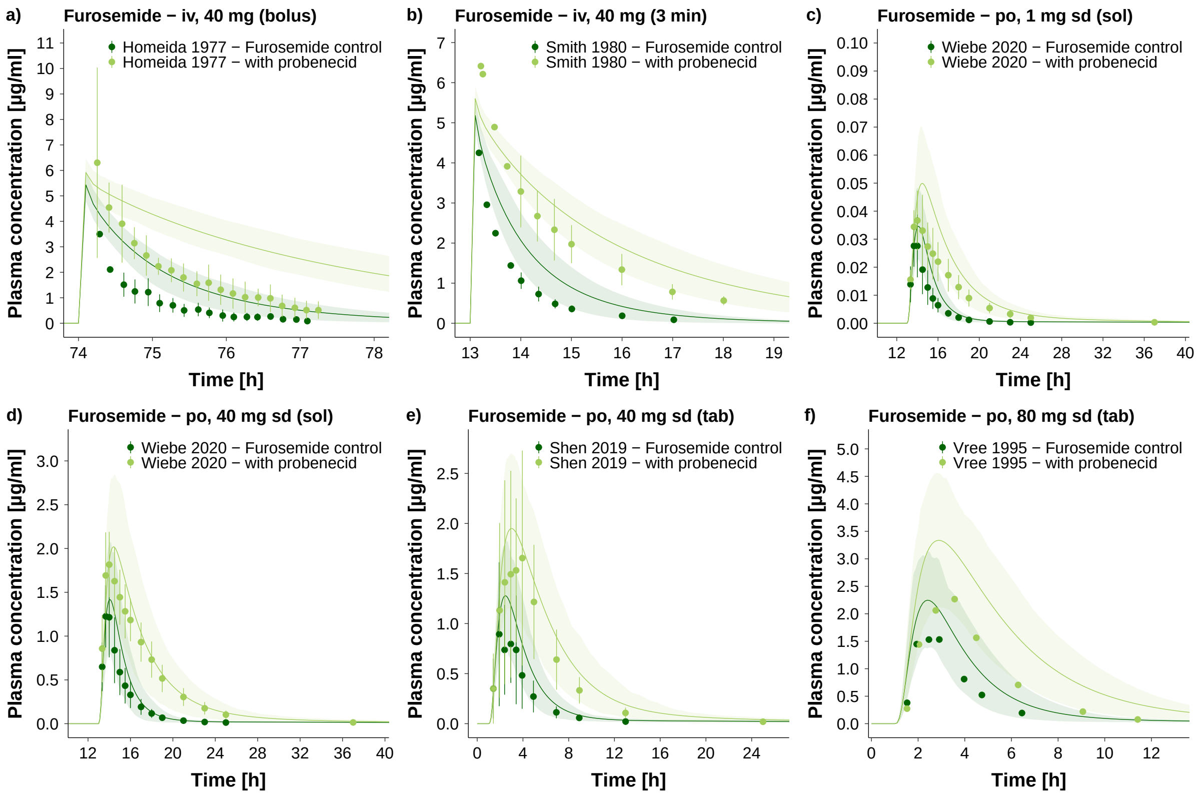

Figure S5.3.2: Probenecid-furosemide DDI. Population predictions of furosemide plasma concentration-time profiles compared to observed data. Observed data are shown as dots ± standard deviation. Population simulation arithmetic means are shown as lines; the shaded areas illustrate the predicted population variation ($Q_{16} - Q_{84}$). Details on dosing regimens, study populations and literature references are summarized in Table S5.2.1.

5.3.3 Semilogarithmic plots - Plasma - Individual predictions

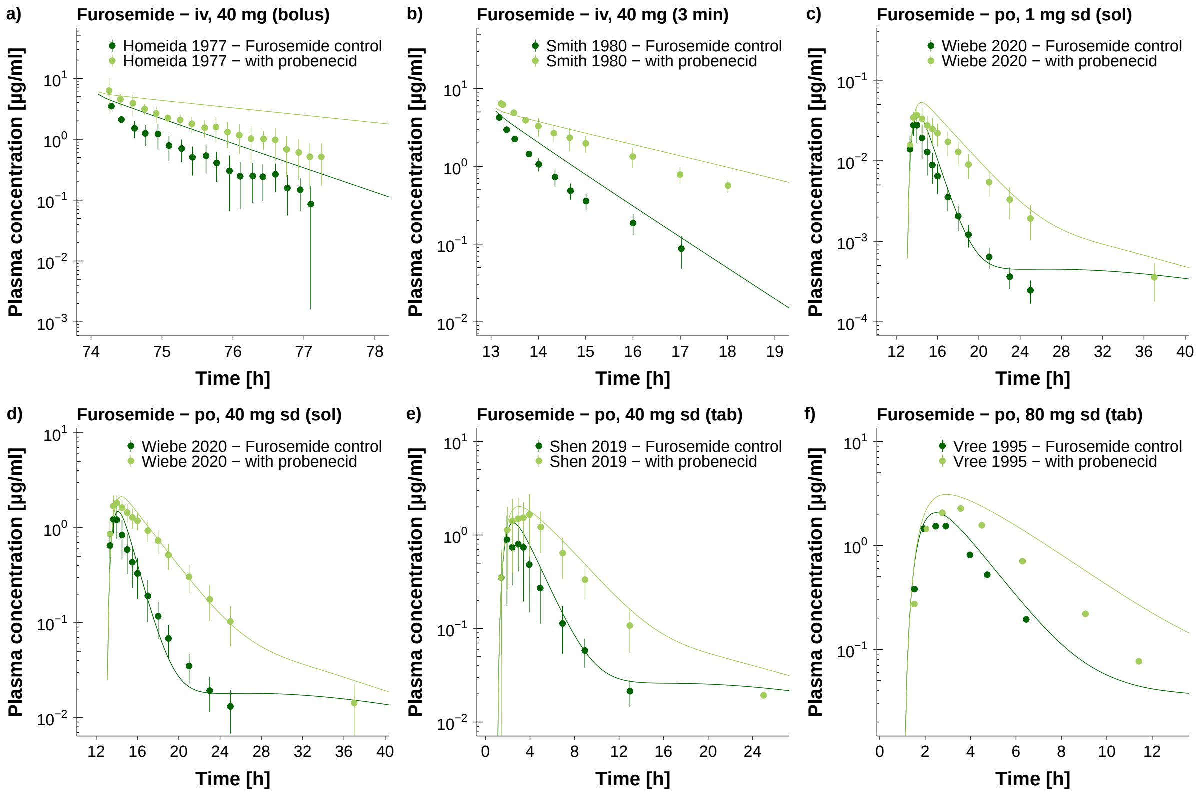

Figure S5.3.3: Probenecid-furosemide DDI. Individual predictions of furosemide plasma concentration-time profiles compared to observed data. Observed data are shown as dots ± standard deviation. Simulations are shown as lines. Details on dosing regimens, study populations and literature references are summarized in Table S5.2.1.

5.3.4 Linear plots - Plasma - Individual predictions

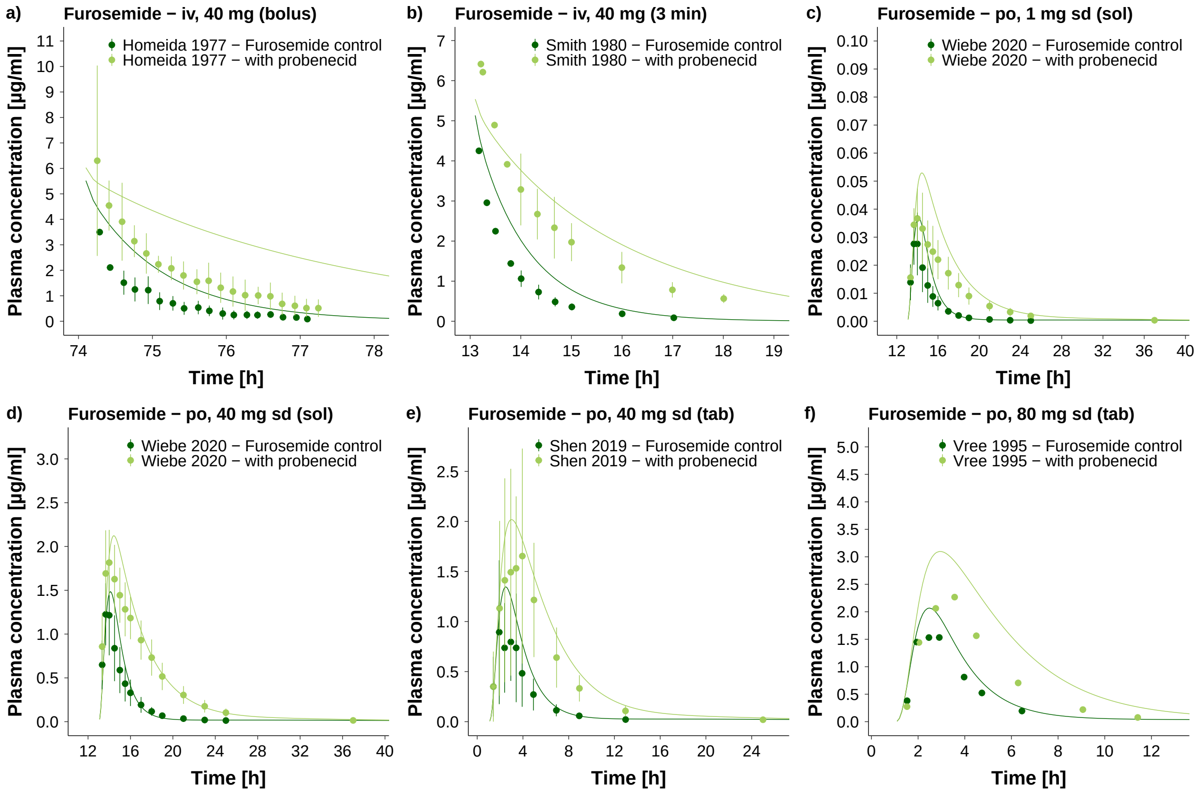

Figure S5.3.4: Probenecid-furosemide DDI. Individual predictions of furosemide plasma concentration-time profiles compared to observed data. Observed data are shown as dots ± standard deviation. Simulations are shown as lines. Details on dosing regimens, study populations and literature references are summarized in Table S5.2.1.

5.3.5 Linear plots - Fraction excreted unchanged in urine - Population predictions

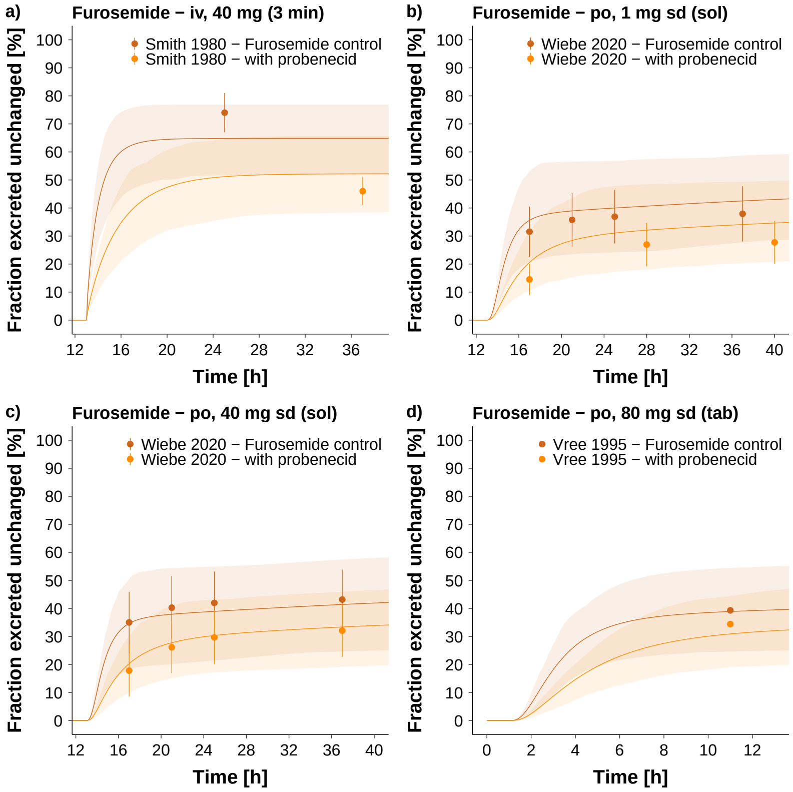

Figure S5.3.5: Probenecid-furosemide DDI. Population predictions of furosemide fraction excreted unchanged in urine profiles compared to observed data. Observed data are shown as dots $\pm$ standard deviation. Population simulation arithmetic means are shown as lines; the shaded areas illustrate the predicted population variation ($Q_{16} - Q_{84}$). Details on dosing regimens, study populations and literature references are summarized in Table S5.2.1.

5.3.6 Linear plots - Fraction excreted unchanged in urine - Individual predictions

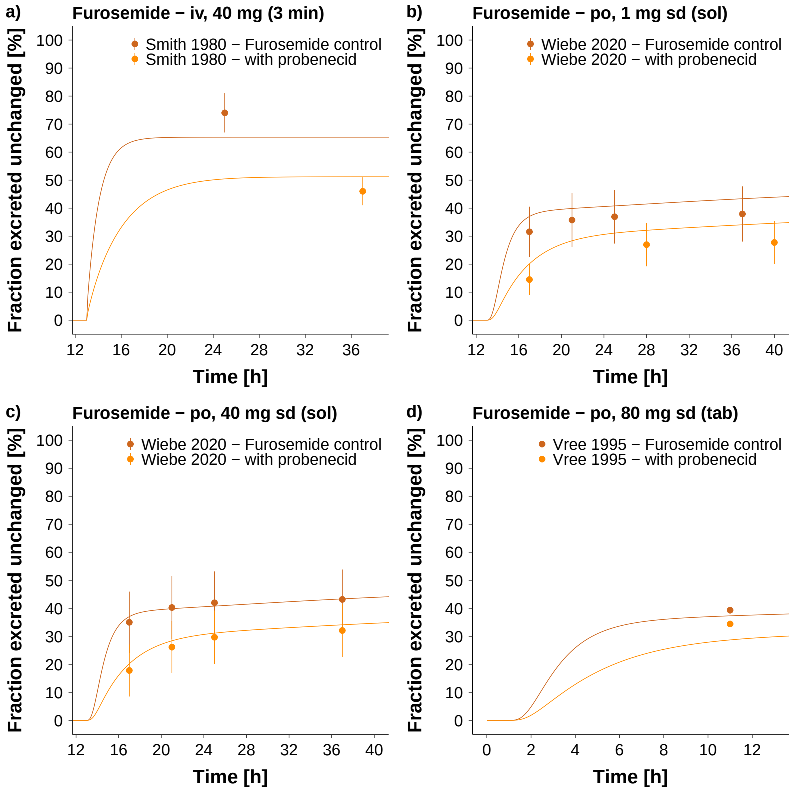

Figure S5.3.6: Probenecid-furosemide DDI. Individual predictions of furosemide fraction excreted unchanged in urine profiles compared to observed data. Observed data are shown as dots ± standard deviation. Simulations are shown as lines. Details on dosing regimens, study populations and literature references are summarized in Table S5.2.1.

5.4 PBPK DDI performance evaluation

5.4.1 DDI AUC_{last} and DDI C_{max} ratio goodness-of-fit plots

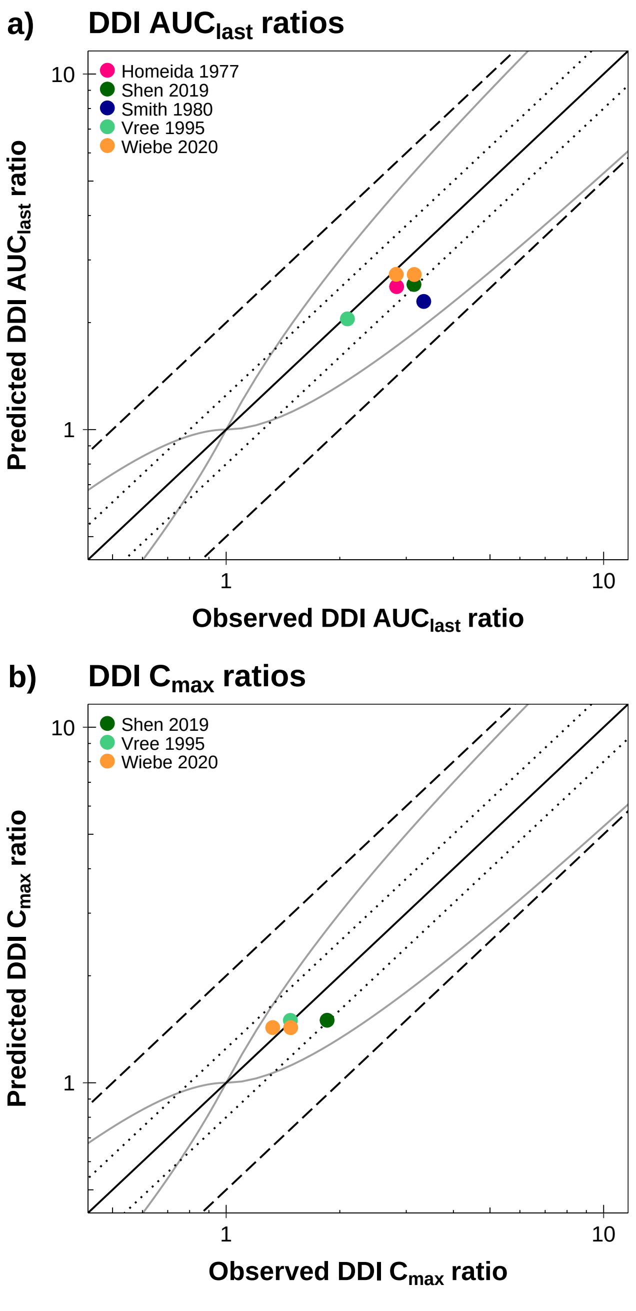

Figure S5.4.1: DDI AUC_{last} and DDI C_{max} ratios. Predicted compared to observed (a) DDI AUC_{last} ratios and (b) DDI C_{max} ratios of all analyzed probenecid-furosemide DDI studies. The solid line marks the line of identity. The dotted lines indicate 1.25-fold, the dashed lines indicate 2-fold deviation. The curved grey lines show the prediction acceptance limits proposed by Guest et al. [103]. Details on dosing regimens, study populations and literature references are summarized in Table S5.2.1. The individual DDI AUC_{last} and DDI C_{max} ratios, mean GMFE values and ranges are listed in Table S5.4.1.

5.4.2 Predicted and observed DDI AUC_{last} and DDI C_{max} ratios with mean GMFE values and ranges

Table S5.4.1: Predicted and observed probenecid-furosemide DDI AUC_{last} ratios and DDI C_{max} ratios

**Probenecid administration**		**Furosemide administration**			**DDI AUC_{last} ratio**			**DDI C_{max} ratio**				**Reference**
Dose [mg]	***Route***	***Dose [mg]***	***Route***	**Dose gap [h]**	***Pred***	***Obs***	***Pred/Obs***	***Pred***	***Obs***	***Pred/Obs***	**t_{last} [h]**	
Intravenous												
500<br>1000	po (-), qid (D1-D3)<br>po (-), sd (D4)	40	iv (bolus), sd (D4)	2	2.52	2.83	0.89	-	-	-	3.0	Homeida 1977 [43]
1000	po (tab), bid (D1)	40	iv (3 min), sd (D1)	1	2.29	3.34	0.69	-	-	-	4.0	Smith 1980 [24]
**GMFE**							**1.29 (1.12 - 1.45)**<br>**2/2 with GMFE $\leq$ 2**					
Oral												
1000	po (tab), bid (D1)	1	po (sol), sd (D1)	1	2.73	2.82	0.97	1.43	1.33	1.08	12.0	Wiebe 2020 [11]
1000	po (tab), bid (D1)	40	po (sol), sd (D1)	1	2.73	3.15	0.87	1.43	1.48	0.97	12.0	Wiebe 2020 [11]
1000	po (tab), sd (D1)	40	po (tab), sd (D1)	1	2.56	3.14	0.82	1.50	1.85	0.81	12.0	Shen 2019 [23]
1000	po (tab), sd (D1)	80	po (tab), sd (D1)	1	2.05	2.10	0.98	1.50	1.48	1.01	5.0	Vree 1995 [33]
**GMFE**							**1.11 (1.02 - 1.22)**<br>**4/4 with GMFE $\leq$ 2**			**1.09 (1.01 - 1.23)**<br>**4/4 with GMFE $\leq$ 2**		
**Overall GMFE**							**1.17 (1.02 - 1.45)**<br>**6/6 with GMFE $\leq$ 2**			**1.09 (1.01 - 1.23)**<br>**4/4 with GMFE $\leq$ 2**		

AUC_{last}: area under the plasma concentration-time curve (AUC) from the time of drug administration to the time of the last concentration measurement, **bid:** twice daily, **C_{max}:** peak plasma concentration, **D:** day of administration, **GMFE:** geometric mean fold error, **iv:** intravenous, **obs:** observed, **po:** oral, **pred:** predicted, **qid:** four times daily, **route:** route of administration, **sd:** single dose, **sol:** solution, **tab:** tablet, **t_{last}:** time of the last concentration measurement. GMFE values are means and ranges.

6 Probenecid-rifampicin DDI

6.1 PBPK DDI modeling

To predict the probenecid-rifampicin DDI, competitive inhibition of OATP1B1 by probenecid was implemented using a K_i of 39.8 µmol/l. This probenecid OATP1B1 K_i was determined in OATP1B1-expressing HEK293 cells, with 2´,7´-dichlorofluorescein as the substrate (3 µmol/l) and 1-1000 µmol/l of probenecid [10].

Details on dosing regimen, study population and literature reference of the predicted clinical DDI study are summarized in Table S6.2.1. Plots of population predicted compared to observed rifampicin plasma concentration-time profile of the probenecid-rifampicin DDI study are shown in semilogarithmic and linear plots in Figure S6.3.1. Plots of individual predicted compared to observed rifampicin plasma concentration-time profile of the probenecid-rifampicin DDI study are shown in semilogarithmic and linear plots in Figure S6.3.2. The correlation of predicted to observed DDI AUC_{last} ratios and DDI C_{max} ratios is shown in Figure S6.4.1. The DDI AUC_{last} ratios and DDI C_{max} ratios with GMFE values are summarized in Table S6.4.1.

6.2 Clinical studies

Table S6.2.1: Probenecid-rifampicin DDI study table

**Probenecid administration**		**Rifampicin administration**		**Dose gap [h]**	**n**	**Age [years]**	**Weight [kg]**	**Height [cm]**	**Females [%]**	**Dataset**	**Reference**
Dose [mg]	***Route***	***Dose [mg]***	***Route***								
2000<br>1500	po (-), sd (D2),<br>po (-), sd (D2)[a]	300	po (tab), qd (D1-D2)	0.33	6	24-40	-	-	0	test	Kenwright 1973 [104]

D: day of administration, **n:** number of individuals studied, **po:** oral, **qd:** once daily, **route:** route of administration, **sd:** single dose, **tab:** tablet, **test:** test dataset (model evaluation). Values are means ± standard deviation or ranges.

[a] 2000 mg probenecid 0.33 h before and 1500 mg 6 h after rifampicin administration

6.3 Profiles

6.3.1 Semilogarithmic and linear plots - Plasma - Population predictions

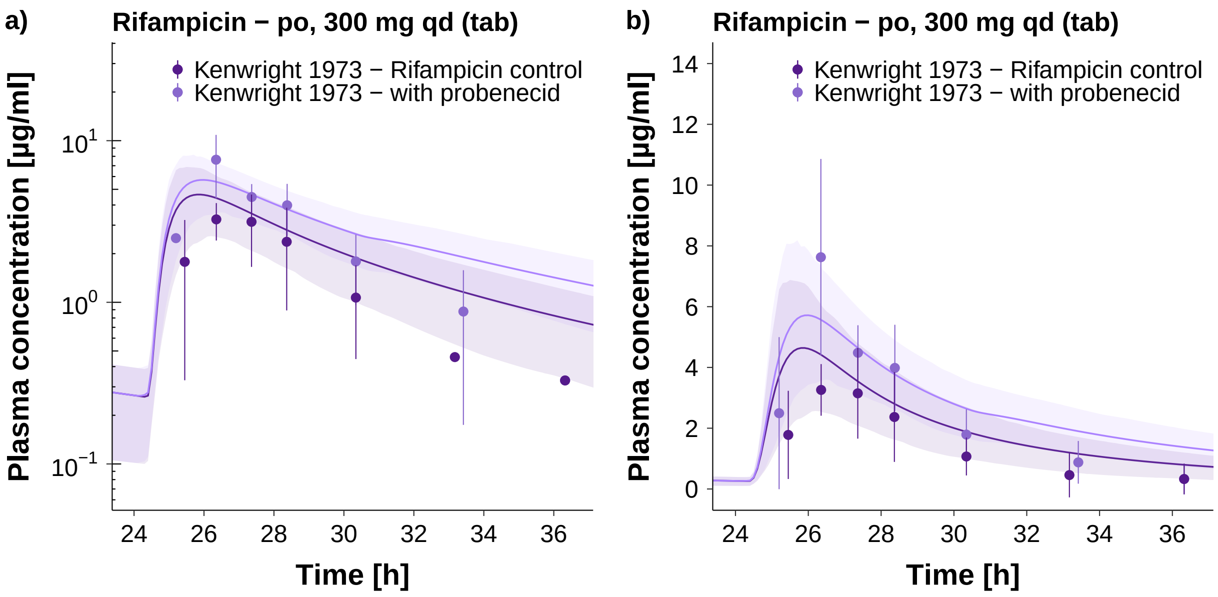

Figure S6.3.1: Probenecid-rifampicin DDI. Population predictions of rifampicin plasma concentration-time profiles compared to observed data. Observed data are shown as dots ± standard deviation. Population simulation arithmetic means are shown as lines; the shaded areas illustrate the predicted population variation ($Q_{16} - Q_{84}$). Details on dosing regimen, study population and literature reference are summarized in Table S6.2.1.

6.3.2 Semilogarithmic and linear plots - Plasma - Individual predictions

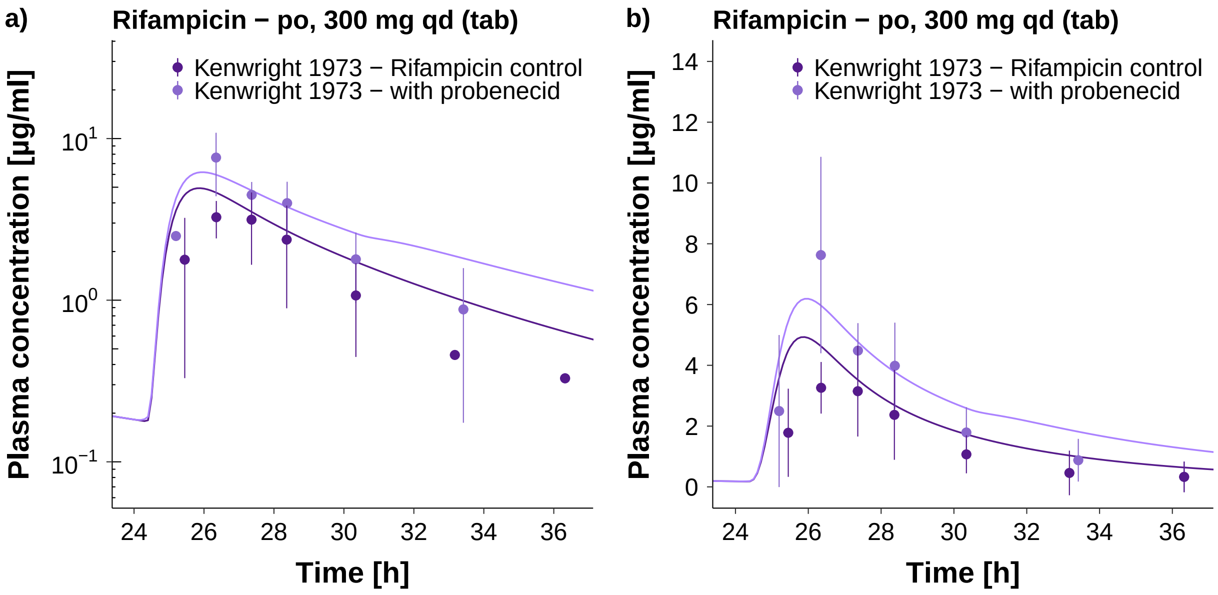

Figure S6.3.2: Probenecid-rifampicin DDI. Individual predictions of rifampicin plasma concentration-time profiles compared to observed data. Observed data are shown as dots ± standard deviation. Simulations are shown as lines. Details on dosing regimen, study population and literature reference are summarized in Table S6.2.1.

6.4 PBPK DDI performance evaluation

6.4.1 DDI AUC_{last} and DDI C_{max} ratio goodness-of-fit plots

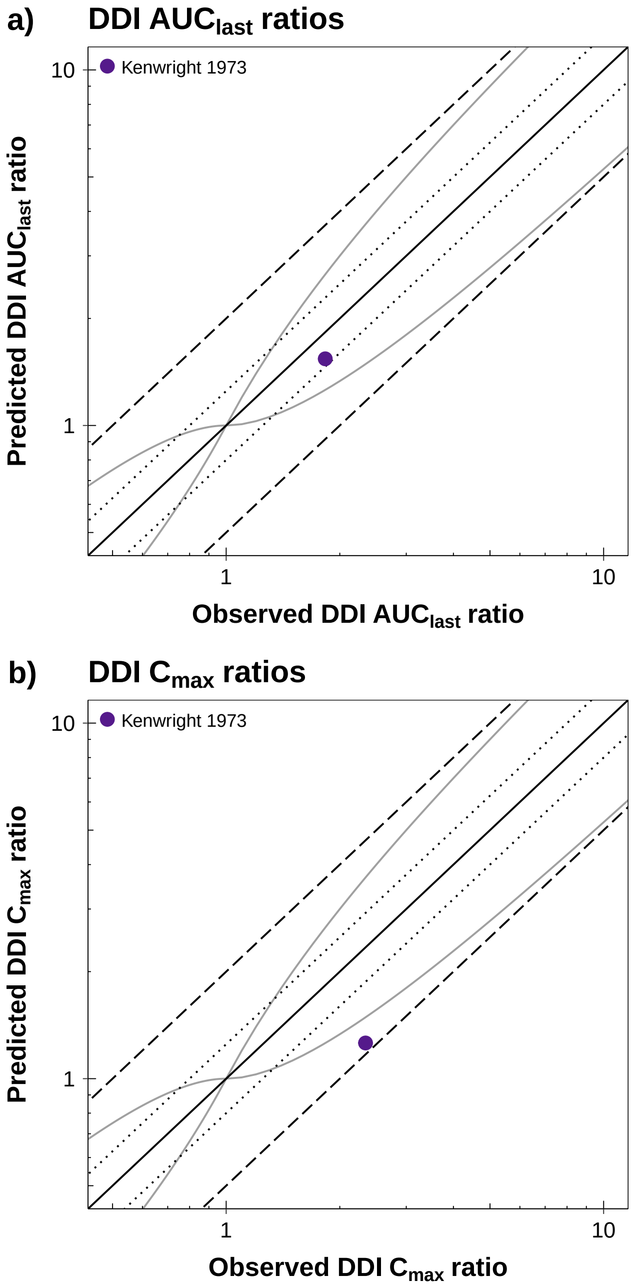

Figure S6.4.1: DDI AUC_{last} and DDI C_{max} ratios. Predicted compared to observed (a) DDI AUC_{last} ratios and (b) DDI C_{max} ratios of the probenecid-rifampicin DDI study. The solid line marks the line of identity. The dotted lines indicate 1.25-fold, the dashed lines indicate 2-fold deviation. The curved grey lines show the prediction acceptance limits proposed by Guest et al. [103]. Details on dosing regimen, study population and literature reference are summarized in Table S6.2.1. The individual DDI AUC_{last} and DDI C_{max} ratios and GMFE values are listed in Table S6.4.1.

6.4.2 Predicted and observed DDI AUC_{last} and DDI C_{max} ratios with GMFE values

Table S6.4.1: Predicted and observed probenecid-rifampicin DDI AUC_{last} ratios and DDI C_{max} ratios

**Probenecid administration**		**Rifampicin administration**			**DDI AUC_{last} ratio**			**DDI C_{max} ratio**				**Reference**
Dose [mg]	***Route***	***Dose [mg]***	***Route***	**Dose gap [h]**	***Pred***	***Obs***	***Pred/Obs***	***Pred***	***Obs***	***Pred/Obs***	**t_{last} [h]**	
2000<br>1500	po (-), sd (D2),<br>po (-), sd (D2)[a]	300	po (tab), qd (D1-D2)	0.33	1.54	1.83	0.84	1.26	2.34	0.54	11.0	Kenwright 1973 [104]
**GMFE**							**1.19**<br>**1/1 with GMFE $\leq$ 2**			**1.85**<br>**1/1 with GMFE $\leq$ 2**		

AUC_{last}: area under the plasma concentration-time curve (AUC) from the time of drug administration to the time of the last concentration measurement, **C_{max}:** peak plasma concentration, **D:** day of administration, **GMFE:** geometric mean fold error, **obs:** observed, **po:** oral, **pred:** predicted, **qd:** once daily, **route:** route of administration, **sd:** single dose, **tab:** tablet, **t_{last}:** time of the last concentration measurement.

[a] 2000 mg probenecid 0.33 h before and 1500 mg 6 h after rifampicin administration

7 System-dependent parameters

Table S7.0.1: System-dependent parameters

	Reference concentration					Half-life	
**Enzyme/Transporter**	**Mean[a]**	**GeoSD[b]**	**Relative expression[c]**	**Localization**	**Direction**	**Liver [h]**	**Intestine [h]**
**Enzyme**							
AADAC	1.00[d] [7]	1.40[e]	RT-PCR [107]	Intracellular	-	36	23
UGT1A9	0.20[f] [105, 108]	1.12 [4]	RT-PCR [107]	Intracellular	-	36	23
**Transporter**							
MRP4	0.02[f] [105, 109]	1.89 [109]	RT-PCR [110]	Basolateral, in renal tubule cells apical	Efflux	36	23
OAT3	0.09[f] [105, 109]	1.53 [109]	RT-PCR [110]	Basolateral	Influx	36	-
OATP1B1	0.07[g] [106]	1.54 [106]	RT-PCR [110]	Basolateral	Influx	36	-
Pgp (efflux)	1.41 [81]	1.60 [106]	RT-PCR [110], with the relative expression in intestinal mucosa increased by factor 3.57 (optimized)	Apical	Efflux	36	23

AADAC: arylacetamide deacetylase, **MRP4:** multidrug resistance-associated protein 4, **OAT3:** organic anion transporter 3, **OATP1B1:** organic anion transporting polypeptide 1B1, **Pgp:** P-glycoprotein, **RT-PCR:** reverse transcription-polymerase chain reaction, **UGT1A9:** uridine 5'-diphospho-glucuronosyltransferase 1A9

[a] µmol/l in the tissue of highest expression

[b] Geometric standard deviation of the reference concentration

[c] Relative expression in the different organs (PK-Sim® expression database profile)

[d] If no information was available, the mean reference concentration was set to 1.00 µmol/l and the catalytic rate constant (k_{cat}) was optimized according to [7]

[e] If no information was available, a moderate variability of 35% CV was assumed (= 1.40 GSD)

[f] Calculated from transporter/enzyme per mg membrane protein x 26.2 mg human kidney microsomal protein per g kidney [105]

[g] Calculated from transporter per mg membrane protein x 37.0 mg membrane protein per g liver [106]

8 Abbreviations

**ADME**	Absorption, distribution, metabolism and excretion
**AUC**	Area under the plasma concentration-time curve
**AUC_{0-12}**	Area under the plasma concentration-time curve from 0 to 12 h
**AUC_{0-24}**	Area under the plasma concentration-time curve from 0 to 24 h
**AUC_{last}**	Area under the plasma concentration-time curve from the time of drug administration to the time of the last concentration measurement
**bid**	Twice daily
**C_{max}**	Peak plasma concentration
**CYP**	Cytochrome P450
**DDI**	Drug-drug interaction
**f_u**	Fraction unbound in plasma
**GFR**	Glomerular filtration rate
**GMFE**	Geometric mean fold error
**HEK293**	Human embryonic kidney 293 cell line
**ICRP**	International Commission on Radiological Protection
**iv**	Intravenous
**k_{cat}**	Catalytic rate constant
**K_i**	Inhibition constant
**K_M**	Michaelis-Menten constant
**$K_{M,app}$**	Michaelis-Menten constant in the presence of inhibitor
**logP**	Lipophilicity
**MRD**	Mean relative deviation
**MRP4**	Multidrug resistance-associated protein 4
**OAT**	Organic anion transporter
**OATP1B1**	Organic anion transporting polypeptide 1B1
**PBPK**	Physiologically based pharmacokinetic
**PK**	Pharmacokinetics
**po**	Oral
**qd**	Once daily
**T_{max}**	Time to peak plasma concentration
**UGT1A1**	Uridine 5’-diphospho-glucuronosyltransferase 1A1
**UGT1A9**	Uridine 5’-diphospho-glucuronosyltransferase 1A9
**v**	Reaction velocity

$\mathbf{V_D}$ Volume of distribution

$\mathbf{v_{max}}$ Maximum reaction velocity

$\mathbf{v_{max,app}}$ Maximum reaction velocity in the presence of inhibitor

Bibliography

[1] T Eissing, L Kuepfer, C Becker, M Block, K Coboeken, T Gaub, L Goerlitz, J Jaeger, R Loosen, B Ludewig, M Meyer, C Niederalt, M Sevestre, HU Siegmund, J Solodenko, K Thelen, U Telle, W Weiss, T Wendl, S Willmann, and J Lippert. A computational systems biology software platform for multiscale modeling and simulation: integrating whole-body physiology, disease biology, and molecular reaction networks. *Frontiers in physiology*, 2(February):1–10, 2011.

[2] J Lippert, R Burghaus, A Edginton, S Frechen, M Karlsson, A Kovar, T Lehr, P Milligan, V Nock, S Ramusovic, M Riggs, S Schaller, J Schlender, S Schmidt, M Sevestre, E Sjögren, J Solodenko, A Staab, and D Teutonico. Open systems pharmacology community - An open access, open source, open science approach to modeling and simulation in pharmaceutical sciences. *CPT: pharmacometrics & systems pharmacology*, 8(12):878–882, 2019.

[3] JG Wojtyniak, H Britz, D Selzer, M Schwab, and T Lehr. Data digitizing: Accurate and precise data extraction for quantitative systems pharmacology and physiologically-based pharmacokinetic modeling. *CPT: Pharmacometrics & Systems Pharmacology*, 9(6):322–331, 2020.

[4] Open Systems Pharmacology Suite Community. Open Systems Pharmacology Suite Manual, 2018. URL `https://docs.open-systems-pharmacology.org/`.

[5] J Valentin. Basic anatomical and physiological data for use in radiological protection: reference values. A report of age- and gender-related differences in the anatomical and physiological characteristics of reference individuals. ICRP Publication 89. *Annals of the ICRP*, 32 (3-4):5–265, 2002.

[6] S Willmann, K Höhn, A Edginton, M Sevestre, J Solodenko, W Weiss, J Lippert, and W Schmitt. Development of a physiology-based whole-body population model for assessing the influence of individual variability on the pharmacokinetics of drugs. *Journal of pharmacokinetics and pharmacodynamics*, 34(3):401–31, 2007.

[7] M Meyer, S Schneckener, B Ludewig, L Kuepfer, and J Lippert. Using expression data for quantification of active processes in physiologically based pharmacokinetic modeling. *Drug metabolism and disposition: the biological fate of chemicals*, 40(5):892–901, 2012.

[8] G Tanaka and H Kawamura. Anatomical and physiological characteristics for asian reference man: male and female of different ages: Tanaka model. *Division of Radioecology. National Institute of Radiological Sciences. Hitachinaka 311-12 Japan.*, pages NIRS–M–115, 1996.

[9] Y Tsuruya, K Kato, Y Sano, Y Imamura, K Maeda, Y Kumagai, Y Sugiyama, and H Kusuhara. Investigation of endogenous compounds applicable to drug-drug interaction studies involving the renal organic anion transporters, OAT1 and OAT3, in humans. *Drug Metabolism and Disposition*, 44(12):1825–1933, 2016.

[10] S Izumi, Y Nozaki, T Komori, O Takenaka, K Maeda, H Kusuhara, and Y Sugiyama. Investigation of fluorescein derivatives as substrates of organic anion transporting polypeptide (OATP) 1B1 to develop sensitive fluorescence-based OATP1B1 inhibition assays. *Molecular Pharmaceutics*, 13(2):438–448, 2016.

[11] ST Wiebe, T Giessmann, K Hohl, S Schmidt-Gerets, E Hauel, A Jambrecina, K Bader, N Ishiguro, ME Taub, A Sharma, T Ebner, G Mikus, MF Fromm, F Müller, and P Stopfer. Validation of a drug transporter probe cocktail using the prototypical inhibitors rifampin, probenecid, verapamil, and cimetidine. *Clinical Pharmacokinetics*, 2020.

[12] RF Cunningham, ZH Israili, and PG Dayton. Clinical Pharmacokinetics of Probenecid. *Clinical Pharmacokinetics*, 6(2):135–51, 1981.

[13] Biokanol® Pharma GmbH. Fachinformation: Probenecid Weimer®, 2010.

[14] TB Vree, EW Van Ewijk-Beneken Kolmer, EW Wuis, and YA Hekster. Capacity-limited renal glucuronidation of probenecid by humans. A pilot Vmax-finding study. *Pharmaceutisch weekblad. Scientific edition*, 14(5):325–31, 1992.

[15] BM Emanuelsson, B. Beermann, and LK Paalzow. Non-linear elimination and protein binding of probenecid. *European Journal of Clinical Pharmacology*, 32(4):395–401, 1987.

[16] Y Ito, T Fukami, T Yokoi, and M Nakajima. An orphan esterase ABHD10 modulates probenecid acyl glucuronidation in human liver. *Drug Metabolism and Disposition*, 42(12): 2109–116, 2014.

[17] JM Perel, RF Cunningham, HM Fales, and PG Dayton. Identification and renal excretion of probenecid metabolites in man. *Life sciences. Pt. 1: Physiology and pharmacology*, 9(23): 1337–43, 1970.

[18] A Selen, GL Amidon, and PG Wellingx. Pharmacokinetics of probenecid following oral doses to human volunteers. *Journal of Pharmaceutical Sciences*, 71(11):1238–42, 1982.

[19] U.S. Food and Drug Administration. Drug development and drug interactions: table of substrates, inhibitors and inducers, 2017. URL `https://www.fda.gov/Drugs/DevelopmentApprovalProcess/DevelopmentResources/DrugInteractionsLabeling/ucm093664.htm`.

[20] TB Vree, EW Van Ewijk-Beneken Kolmer, EW Wuis, YA Hekster, and MM Broekman. Interindividual variation in the capacity-limited renal glucuronidation of probenecid by humans. *Pharmacy world & science : PWS*, 15(5):197–202, 1993.

[21] PG Dayton, TF Yu, W Chen, L Berger, LA West, and AB Gutman. The physiological disposition of probenecid, including renal clearance, in man, studied by an improved method for its estimation in biological material. *The Journal of pharmacology and experimental therapeutics*, 140:278–86, 1963.

[22] CB Landersdorfer, CMJ Kirkpatrick, M Kinzig, JB Bulitta, U Holzgrabe, U Jaehde, A Reiter, KG Naber, M Rodamer, and F Sörgel. Competitive inhibition of renal tubular secretion of ciprofloxacin and metabolite by probenecid. *British Journal of Clinical Pharmacology*, 69(2): 167–78, 2010.

[23] H Shen, VK Holenarsipur, TT Mariappan, DM Drexler, JL Cantone, P Rajanna, S Singh Gautam, Y Zhang, J Gan, PA Shipkova, P Marathe, and WG Humphreys. Evidence for the validity of pyridoxic acid (PDA) as a plasma-based endogenous probe for OAT1 and OAT3 function in healthy subjects. *Journal of Pharmacology and Experimental Therapeutics*, 368 (1):136–45, 2019.

[24] DE Smith, WL Gee, DC Brater, ET Lin, and LZ Benet. Preliminary evaluation of furosemide–probenecid interaction in humans. *Journal of Pharmaceutical Sciences*, 69(5): 571–75, 1980.

[25] CB Landersdorfer, CMJ Kirkpatrick, M Kinzig, JB Bulitta, U Holzgrabe, GL Drusano, and F Sorgel. Competitive inhibition of renal tubular secretion of gemifloxacin by probenecid. *Antimicrobial Agents and Chemotherapy*, 53(9):3902–07, 2009.

[26] DS Wishart, C Knox, AC Guo, S Shrivastava, M Hassanali, P Stothard, Z Chang, and J Woolsey. DrugBank: a comprehensive resource for in silico drug discovery and exploration. *Nucleic acids research*, 34(Database issue):D668–72, 2006.

[27] A Avdeef. Physicochemical profiling (solubility, permeability and charge state). *Current Topics in Medicinal Chemistry*, 1(4):277–351, 2001.

[28] A Avdeef. *Absorption and drug development - Solubility, permeability, and charge state*. 2003.

[29] E Söderlind, E Karlsson, A Carlsson, R Kong, A Lenz, S Lindborg, and JJ Sheng. Simulating fasted human intestinal fluids: understanding the roles of lecithin and bile acids. *Molecular pharmaceutics*, 7(5):1498–507, 2010.

[30] C Hansch, A Leo, and D Hoekman. *Exploring QSAR: hydrophobic, electronic, steric constants*. 1995.

[31] LL Ponto and RD Schoenwald. Furosemide (frusemide). A pharmacokinetic/pharmacodynamic review (Part I). *Clinical pharmacokinetics*, 18(5):381–408, 1990.

[32] Sanofi-Aventis Deutschland GmbH. Fachinformation: Lasix® long 30 mg Retardkapseln/Lasix® 40 mg Tabletten/Lasix® 500 mg Tabs, 2017.

[33] TB Vree, M van den Biggelaar-Martea, and CPWGM Verwey-van Wissen. Probenecid inhibits the renal clearance of frusemide and its acyl glucuronide. *British journal of clinical pharmacology*, 39(6):692–5, 1995.

[34] G González, A Arancibia, MI Rivas, P Caro, and C Antezana. Pharmacokinetics of furosemide in patients with hepatic cirrhosis. *European journal of clinical pharmacology*, 22(4):315–20, 1982.

[35] C Lambert, P Larochelle, and P du Souich. Effects of phenobarbital and tobacco smoking on furosemide kinetics and dynamics in normal subjects. *Clinical pharmacology and therapeutics*, 34(2):170–5, 1983.

[36] O Kerdpin, K M Knights, D J Elliot, and J O Miners. In vitro characterisation of human renal and hepatic frusemide glucuronidation and identification of the UDP-glucuronosyltransferase enzymes involved in this pathway. *Biochemical Pharmacology*, 76(2):249–257, 2008.

[37] L Haegeli, HP Brunner-La Rocca, M Wenk, M Pfisterer, J Drewe, and S Krähenbühl. Sublingual administration of furosemide: new application of an old drug. *British journal of clinical pharmacology*, 64(6):804–9, 2007.

[38] B Rosenkranz, KH Lehr, G Mackert, and HW Seyberth. Metamizole-furosemide interaction study in healthy volunteers. *European journal of clinical pharmacology*, 42(6):593–8, 1992.

[39] WJ Tilstone and A Fine. Furosemide kinetics in renal failure. *Clinical pharmacology and therapeutics*, 23(6):644–50, 1978.

[40] G Alván, B Beermann, L Hjelte, M Lind, A Lindholm, and B Strandvik. Increased nonrenal clearance and increased diuretic efficiency of furosemide in cystic fibrosis. *Clinical pharmacology and therapeutics*, 44(4):436–41, 1988.

[41] F Andreasen and E Mikkelsen. Distribution, elimination and effect of furosemide in normal subjects and in patients with heart failure. *European journal of clinical pharmacology*, 12(1): 15–22, 1977.

[42] MM Hammarlund, LK Paalzow, and B Odlind. Pharmacokinetics of furosemide in man after intravenous and oral administration. Application of moment analysis. *European journal of clinical pharmacology*, 26(2):197–207, 1984.

[43] M Homeida, C Roberts, and RA Branch. Influence of probenecid and spironolactone on furosemide kinetics and dynamics in man. *Clinical Pharmacology & Therapeutics*, 22(4): 402–9, 1977.

[44] E Keller, G Hoppe-Seyler, R Mumm, and P Schollmeyer. Influence of hepatic cirrhosis and end-stage renal disease on pharmacokinetics and pharmacodynamics of furosemide. *European journal of clinical pharmacology*, 20(1):27–33, 1981.

[45] W Rupp. Pharmacokinetics and pharmacodynamics of Lasix. *Scottish medical journal*, 19 (Suppl 1):5–13, 1974.

[46] ES Waller, SF Hamilton, JW Massarella, MA Sharanevych, RV Smith, GJ Yakatan, and JT Doluisio. Disposition and absolute bioavailability of furosemide in healthy males. *Journal of pharmaceutical sciences*, 71(10):1105–8, 1982.

[47] DE Smith, ET Lin, and LZ Benet. Absorption and disposition of furosemide in healthy volunteers, measured with a metabolite-specific assay. *Drug Metabolism and Disposition*, 8(5): 337–42, 1980.

[48] RA Branch, CJ Roberts, M Homeida, and D Levine. Determinants of response to frusemide in normal subjects. *British journal of clinical pharmacology*, 4(2):121–7, 1977.

[49] MR Kelly, RE Cutler, AW Forrey, and BM Kimpel. Pharmacokinetics of orally administered furosemide. *Clinical pharmacology and therapeutics*, 15(2):178–86, 1974.

[50] A Rane, JP Villeneuve, WJ Stone, AS Nies, GR Wilkinson, and RA Branch. Plasma binding and disposition of furosemide in the nephrotic syndrome and in uremia. *Clinical pharmacology and therapeutics*, 24(2):199–207, 1978.

[51] RK Verbeeck, RV Patwardhan, JP Villeneuve, GR Wilkinson, and RA Branch. Furosemide disposition in cirrhosis. *Clinical pharmacology and therapeutics*, 31(6):719–25, 1982.

[52] F Andreasen, CK Christensen, FK Jakobsen, and CE Mogensen. The use of HPLC to elucidate the metabolism and urinary excretion of furosemide and its metabolic products. *Acta pharmacologica et toxicologica*, 49(3):223–9, 1981.

[53] F Andreasen, U Hansen, SE Husted, and JA Jansen. The pharmacokinetics of frusemide are influenced by age. *British journal of clinical pharmacology*, 16(4):391–7, 1983.

[54] P Stopfer, T Giessmann, K Hohl, S Hutzel, S Schmidt, D Gansser, N Ishiguro, ME Taub, A Sharma, T Ebner, and F Müller. Optimization of a drug transporter probe cocktail: potential screening tool for transporter-mediated drug–drug interactions. *British Journal of Clinical Pharmacology*, 84(9):1941–1949, 2018.

[55] P Stopfer, T Giessmann, K Hohl, A Sharma, N Ishiguro, M E Taub, H Zimdahl-Gelling, D Gansser, M Wein, T Ebner, and F Müller. Pharmacokinetic evaluation of a drug transporter cocktail consisting of digoxin, furosemide, metformin, and rosuvastatin. *Clinical Pharmacology & Therapeutics*, 100(3):259–67, 2016.

[56] ES Waller, JW Massarella, MS Tomkiw, RV Smith, and JT Doluisio. Pharmacokinetics of furosemide after three different single oral doses. *Biopharmaceutics & drug disposition*, 6(2): 109–17, 1985.

[57] U.S. Food and Drug Administration. Clinical pharmacology and biopharmaceutics review - NDA: 21-985 - part 3. 2006.

[58] S Vaidyanathan, M Bartlett, HA Dieterich, CM Yeh, A Antunes, D Howard, and WP Dole. Pharmacokinetic interaction of the direct renin inhibitor aliskiren with furosemide and extended-release isosorbide-5-mononitrate in healthy subjects. *Cardiovascular therapeutics*, 26(4):238–46, 2008.

[59] ES Waller, ML Crismon, RV Smith, MT Bauza, and JT Doluisio. Comparative bioavailability of furosemide from solution and 40 mg tablets with different dissolution characteristics following oral administration in normal men. *Biopharmaceutics & drug disposition*, 9(2):211–8, 1988.

[60] MR Ballester, E Roig, I Gich, M Puntes, J Delgadillo, B Santos, and RM Antonijoan. Randomized, open-label, blinded-endpoint, crossover, single-dose study to compare the pharmacodynamics of torasemide-PR 10 mg, torasemide-IR 10 mg, and furosemide-IR 40 mg, in patients with chronic heart failure. *Drug design, development and therapy*, 9:4291–302, 2015.

[61] M Bindschedler, P Degen, G Flesch, M de Gasparo, and G Preiswerk. Pharmacokinetic and pharmacodynamic interaction of single oral doses of valsartan and furosemide. *European journal of clinical pharmacology*, 52(5):371–8, 1997.

[62] BK Martin, M Uihlein, RM Ings, LA Stevens, and J McEwen. Comparative bioavailability of two furosemide formulations in humans. *Journal of pharmaceutical sciences*, 73(4):437–41, 1984.

[63] A Rakhit, GM Kochak, V Tipnis, and ME Hurley. Inhibition of renal clearance of furosemide by pentopril, an angiotensin-converting enzyme inhibitor. *Clinical pharmacology and therapeutics*, 41(5):580–6, 1987.

[64] U.S. Food and Drug Administration. Clinical pharmacology and biopharmaceutics review - NDA: 21-742 - part 3. 2005.

[65] SE Shoaf, SL Bramer, P Bricmont, and CA Zimmer. Pharmacokinetic and pharmacodynamic interaction between tolvaptan, a non-peptide AVP antagonist, and furosemide or hydrochlorothiazide. *Journal of cardiovascular pharmacology*, 50(2):213–22, 2007.

[66] CY Li, A Basit, A Gupta, Z Gáborik, E Kis, and B Prasad. Major glucuronide metabolites of testosterone are primarily transported by MRP2 and MRP3 in human liver, intestine and kidney. *The Journal of steroid biochemistry and molecular biology*, 191(12):105350, 2019.

[67] A Avdeef and O Tsinman. Miniaturized rotating disk intrinsic dissolution rate measurement: Effects of buffer capacity in comparisons to traditional wood's apparatus. *Pharmaceutical Research*, 25(11):2613–627, 2008.

[68] KJ Box, G Völgyi, E Baka, M Stuart, K Takács-Novák, and JEA Comer. Equilibrium versus kinetic measurements of aqueous solubility, and the ability of compounds to supersaturate in solution - a validation study. *Journal of pharmaceutical sciences*, 95(6):1298–307, 2006.

[69] K Takács-Novák, V Szőke, G Völgyi, P Horváth, R Ambrus, and P Szabó-Révész. Biorelevant solubility of poorly soluble drugs: rivaroxaban, furosemide, papaverine and niflumic acid. *Journal of pharmaceutical and biomedical analysis*, 83:279–85, 2013.

[70] A Berthod, S Carda-Broch, and MC Garcia-Alvarez-Coque. Hydrophobicity of ionizable compounds. A theoretical study and measurements of diuretic octanol-water partition coefficients by countercurrent chromatography. *Analytical Chemistry*, 71(4):879–88, 1999.

[71] R Ventura and J Segura. Detection of diuretic agents in doping control. *Journal of Chromatography B: Biomedical Sciences and Applications*, 687(1):127–44, 1996.

[72] F Andreasen, CK Christensen, FK Jacobsen, J Jansen, CE Mogensen, and OL Pedersen. The individual variation in pharmacokinetics and pharmacodynamics of furosemide in young normal male subjects. *European journal of clinical investigation*, 12(3):247–55, 1982.

[73] TB Vree, M van den Biggelaar-Martea, and CPWGM Verwey-van Wissen. Determination of furosemide with its acyl glucuronide in human plasma and urine by means of direct gradient high-performance liquid chromatographic analysis with fluorescence detection. Preliminary pharmacokinetics and effect of probenecid. *Journal of chromatography. B, Biomedical applications*, 655(1):53–62, 1994.

[74] GM Pacifici, A Viani, HU Schulz, and HJ Frercks. Plasma protein binding of furosemide in the elderly. *European journal of clinical pharmacology*, 32(2):199–202, 1987.

[75] F Andreasen and P Jakobsen. Determination of furosemide in blood plasma and its binding to proteins in normal plasma and in plasma from patients with acute renal failure. *Acta Pharmacologica et Toxicologica*, 35(1):49–57, 1974.

[76] AW Forrey, B Kimpel, AD Blair, and RE Cutler. Furosemide concentrations in serum and urine, and its binding by serum proteins as measured fluorometrically. *Clinical chemistry*, 20 (2):152–8, 1974.

[77] T Ebner, N Ishiguro, and ME Taub. The use of transporter probe drug cocktails for the assessment of transporter-based drug–drug interactions in a clinical setting - Proposal of a four component transporter cocktail. *Journal of Pharmaceutical Sciences*, 104(9):3220–3228, 2015.

[78] W Schmitt. General approach for the calculation of tissue to plasma partition coefficients. *Toxicology in vitro : an international journal published in association with BIBRA*, 22(2):457–67, 2008.

[79] PJ McNamara, TS Foster, GA Digenis, RB Patel, WA Craig, PG Welling, RS Rapaka, VK Prasad, and VP Shah. Influence of tablet dissolution on furosemide bioavailability: a bioequivalence study. *Pharmaceutical research*, 4(2):150–3, 1987.

[80] F Langenbucher. Linearization of dissolution rate by the Weibull distribution. *J Pharm Pharmac*, 24:979, 1972.

[81] N Hanke, S Frechen, D Moj, H Britz, T Eissing, T Wendl, and T Lehr. PBPK Models for CYP3A4 and P-gp DDI prediction: a modeling network of rifampicin, itraconazole, clarithromycin, midazolam, alfentanil, and digoxin. *CPT: pharmacometrics & systems pharmacology*, 7(10):647–59, 2018.

[82] H Britz, N Hanke, AK Volz, O Spigset, M Schwab, T Eissing, T Wendl, S Frechen, and T Lehr. Physiologically-based pharmacokinetic models for CYP1A2 drug-drug interaction prediction: a modeling network of fluvoxamine, theophylline, caffeine, rifampicin, and midazolam. *CPT: pharmacometrics & systems pharmacology*, 8(5):296–307, 2019.

[83] D Türk, N Hanke, S Wolf, S Frechen, T Eissing, T Wendl, M Schwab, and T Lehr. Physiologically based pharmacokinetic models for prediction of complex CYP2C8 and OATP1B1 (SLCO1B1) drug–drug–gene interactions: a modeling network of gemfibrozil, repaglinide, pioglitazone, rifampicin, clarithromycin and itraconazole. *Clinical Pharmacokinetics*, 58(12): 1595–1607, 2019.

[84] N Hanke, D Türk, D Selzer, S Wiebe, É Fernandez, P Stopfer, V Nock, and T Lehr. A Mechanistic, Enantioselective, Physiologically Based Pharmacokinetic Model of Verapamil and Norverapamil, Built and Evaluated for Drug–Drug Interaction Studies. *Pharmaceutics*, 12(6):556, 2020.

[85] L Kovar, C Schräpel, D Selzer, Y Kohl, R Bals, M Schwab, and T Lehr. Physiologically-Based Pharmacokinetic (PBPK) Modeling of Buprenorphine in Adults, Children and Preterm Neonates. *Pharmaceutics*, 12(6):578, jun 2020.

[86] MJ O'Neil, PE Heckelman, CB Koch, KJ Roman, CM Kenny, and MR D'Arecca. *The Merck Index: An Encyclopedia of Chemicals, Drugs, and Biologicals 14th edn.* 2006.

[87] G Baneyx, N Parrott, C Meille, A Iliadis, and T Lavé. Physiologically based pharmacokinetic modeling of CYP3A4 induction by rifampicin in human: influence of time between substrate and inducer administration. *European journal of pharmaceutical sciences : official journal of the European Federation for Pharmaceutical Sciences*, 56:1–15, 2014.

[88] R Panchagnula, I Gulati, M Varma, and YA Raj. Dissolution methodology for evaluation of rifampicin-containing fixed-dose combinations using biopharmaceutic classification sytem based approach. *Clinical research and regulatory affairs*, 24(2-4):61–76, 2007.

[89] S Agrawal and R Panchagnula. Implication of biopharmaceutics and pharmacokinetics of rifampicin in variable bioavailability from solid oral dosage forms. *Biopharmaceutics & drug disposition*, 26(8):321–34, 2005.

[90] G Boman and VA Ringberger. Binding of rifampicin by human plasma proteins. *European journal of clinical pharmacology*, 7(5):369–73, 1974.

[91] IE Templeton, JB Houston, and A Galetin. Predictive utility of in vitro rifampin induction data generated in fresh and cryopreserved human hepatocytes, Fa2N-4, and HepaRG cells. *Drug metabolism and disposition: the biological fate of chemicals*, 39(10):1921–9, 2011.

[92] M Shou, M Hayashi, Y Pan, Y Xu, K Morrissey, L Xu, and G L Skiles. Modeling, prediction, and in vitro in vivo correlation of CYP3A4 induction. *Drug metabolism and disposition: the biological fate of chemicals*, 36(11):2355–70, 2008.

[93] U Loos, E Musch, JC Jensen, G Mikus, HK Schwabe, and M Eichelbaum. Pharmacokinetics of oral and intravenous rifampicin during chronic administration. *Klinische Wochenschrift*, 63 (23):1205–11, 1985.

[94] RG Tirona, BF Leake, AW Wolkoff, and RB Kim. Human organic anion transporting polypeptide-C (SLC21A6) is a major determinant of rifampin-mediated pregnane X receptor activation. *The Journal of pharmacology and experimental therapeutics*, 304(1):223–8, 2003.

[95] A Nakajima, T Fukami, Y Kobayashi, A Watanabe, M Nakajima, and T Yokoi. Human arylacetamide deacetylase is responsible for deacetylation of rifamycins: rifampicin, rifabutin, and rifapentine. *Biochemical pharmacology*, 82(11):1747–56, 2011.

[96] A Collett, J Tanianis-Hughes, D Hallifax, and G Warhurst. Predicting P-glycoprotein effects on oral absorption: correlation of transport in Caco-2 with drug pharmacokinetics in wild-type and mdr1a(-/-) mice in vivo. *Pharmaceutical research*, 21(5):819–26, 2004.

[97] B Greiner, M Eichelbaum, P Fritz, HP Kreichgauer, O von Richter, J Zundler, and HK Kroemer. The role of intestinal P-glycoprotein in the interaction of digoxin and rifampin. *The Journal of clinical investigation*, 104(2):147–53, 1999.

[98] M Hirano, K Maeda, Y Shitara, and Y Sugiyama. Drug-drug interaction between pitavastatin and various drugs via OATP1B1. *Drug Metabolism and Disposition*, 34(7):1229–1236, 2006.

[99] ML Reitman, X Chu, X Cai, J Yabut, R Venkatasubramanian, S Zajic, J A Stone, Y Ding, R Witter, C Gibson, K Roupe, R Evers, JA Wagner, and A Stoch. Rifampin's acute inhibitory and chronic inductive drug interactions: experimental and model-based approaches to drug-drug interaction trial design. *Clinical pharmacology and therapeutics*, 89(2):234–42, 2011.

[100] T Rodgers, D Leahy, and M Rowland. Physiologically based pharmacokinetic modeling 1: predicting the tissue distribution of moderate-to-strong bases. *Journal of pharmaceutical sciences*, 94(6):1259–76, 2005.

[101] MJ Taylor, S Tanna, and T Sahota. In vivo study of a polymeric glucose-sensitive insulin delivery system using a rat model. *Journal of pharmaceutical sciences*, 99(10):4215–27, 2010.

[102] R Kawai, M Lemaire, J L Steimer, A Bruelisauer, W Niederberger, and M Rowland. Physiologically based pharmacokinetic study on a cyclosporin derivative, SDZ IMM 125. *Journal of pharmacokinetics and biopharmaceutics*, 22(5):327–65, 1994.

[103] EJ Guest, L Aarons, JB Houston, A Rostami-Hodjegan, and A Galetin. Critique of the two-fold measure of prediction success for ratios: application for the assessment of drug-drug interactions. *Drug metabolism and disposition: the biological fate of chemicals*, 39(2):170–3, 2011.

[104] S Kenwright and A J Levi. Impairment of hepatic uptake of rifamycin antibiotics by probenecid, and its therapeutic implications. *Lancet (London, England)*, 2(7843):1401–5, 1973.

[105] D Scotcher, S Billington, J Brown, CR Jones, CDA Brown, A Rostami-Hodjegan, and A Galetin. Microsomal and cytosolic scaling factors in dog and human kidney cortex and application for in vitro-in vivo extrapolation of renal metabolic clearance. *Drug Metabolism and Disposition*, 45(5):556–568, 2017.

[106] B Prasad, R Evers, A Gupta, CECA Hop, L Salphati, S Shukla, SV Ambudkar, and JD Unadkat. Interindividual variability in hepatic organic anion - transporting polypeptides and P-glycoprotein (ABCB1) protein expression: quantification by liquid chromatography tandem mass spectroscopy and influence of genotype, age, and sex. *Drug metabolism and disposition: the biological fate of chemicals*, 42(1):78–88, 2014.

[107] M Nishimura and S Naito. Tissue-specific mRNA expression profiles of human phase I metabolizing enzymes except for cytochrome P450 and phase II metabolizing enzymes. *Drug metabolism and pharmacokinetics*, 21(5):357–74, 2006.

[108] G Margaillan, M Rouleau, J K Fallon, P Caron, L Villeneuve, V Turcotte, P C Smith, M S Joy, and C Guillemette. Quantitative profiling of human renal UDP-glucuronosyltransferases and glucuronidation activity: A comparison of normal and tumoral kidney tissues. *Drug Metabolism and Disposition*, 43(4):611–19, 2015.

[109] B Prasad, K Johnson, S Billington, C Lee, G W Chung, C D A Brown, E J Kelly, J Himmelfarb, and J D Unadkat. Abundance of drug transporters in the human kidney cortex as quantified by quantitative targeted proteomics. *Drug Metabolism and Disposition*, 44(12):1920–1924, 2016.

[110] M Nishimura and S Naito. Tissue-specific mRNA expression profiles of human ATP-binding cassette and solute carrier transporter superfamilies. *Drug metabolism and pharmacokinetics*, 20(6):452–77, 2005.
